# Decoupling HIV-1 antiretroviral drug inhibition from plasma antibody activity to evaluate broadly neutralizing antibody therapeutics and vaccines

**DOI:** 10.1016/j.xcrm.2024.101702

**Published:** 2024-08-30

**Authors:** Magdalena Schwarzmüller, Cristina Lozano, Merle Schanz, Irene A. Abela, Silvan Grosse-Holz, Selina Epp, Martina Curcio, Jule Greshake, Peter Rusert, Michael Huber, Roger D. Kouyos, Huldrych F. Günthard, Alexandra Trkola, the Swiss HIV Cohort Study

**Affiliations:** 1 Institute of Medical Virology, University of Zurich, 8057 Zurich, Switzerland; 2 Department of Infectious Diseases and Hospital Epidemiology, University Hospital Zurich, 8091 Zurich, Switzerland

**Keywords:** HIV-1, neutralizing antibody, antiretroviral therapy

## Abstract

The development of broadly neutralizing antibody (bnAb)-based therapeutic HIV-1 vaccines and cure concepts depends on monitoring bnAb plasma activity in people with HIV (PWH) on suppressive antiretroviral therapy (ART). To enable this, analytical strategies must be defined to reliably distinguish antibody-based neutralization from drug inhibition. Here, we explore strategies that either utilize drug-resistant viruses or remove drugs from plasma. We develop ART-DEX (ART dissociation and size exclusion), an approach which quantitatively separates drugs from plasma proteins following pH-triggered release allowing accurate definition of antibody-based neutralization. We demonstrate that ART-DEX, alone or combined with ART-resistant viruses, provides a highly effective and scalable means of assessing antibody neutralization during ART. Implementation of ART-DEX in standard neutralization protocols should be considered to enhance the analytical capabilities of studies evaluating bnAb therapeutics and therapeutic vaccines, furthering the development of advanced ART and HIV-1 cure strategies.

## Highlights

• ART-DEX is a high-throughput strategy to separate HIV drugs from plasma proteins
• ART-DEX is compatible with standard HIV-1 neutralization protocols
• ART-DEX advances monitoring of neutralizing antibody activity in people with HIV
• Implementing ART-DEX aids therapeutic evaluation of bnAbs and HIV cure strategies

## Graphical abstract

**Figure:**
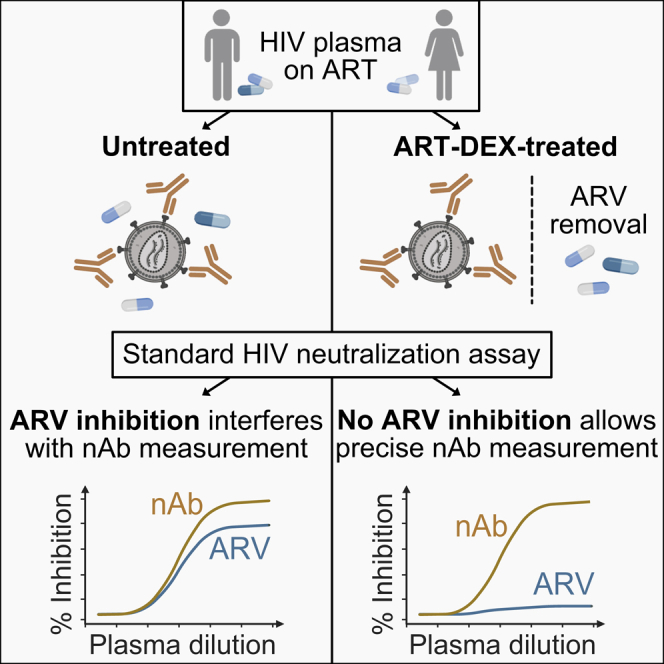

## Teaser

Schwarzmüller et al. report the development of the ART-DEX, a high-throughput method for removing antiretrovirals from the plasma of people with HIV allowing reliable monitoring of antibody-mediated neutralization with conventional neutralization assays. ART-DEX, used alone or with ART-resistant viruses, enhances the ability to evaluate bnAb activity in therapeutic and cure strategies.

## Introduction

Current antiretroviral therapy (ART) is highly effective in suppressing HIV-1 replication to undetectable levels,^1^^,^^2^ reversing CD4^+^ T cell decline,^3^^,^^4^ and reducing disease-related morbidity and mortality.^5^^,^^6^^,^^7^ As a result, the life expectancy of people with HIV (PWH) treated early in the infection has extended to nearly that of people without HIV.^8^^,^^9^^,^^10^ Typically, ART is provided as a combination therapy of drugs targeting one or more HIV-1 enzymes, with nucleoside reverse transcriptase inhibitors (NRTIs), non-nucleoside reverse transcriptase inhibitors (NNRTIs), integrase strand transfer inhibitors (INSTIs), and protease inhibitors (PIs) being widely used.^2^ Over the past decades, a broad portfolio of therapeutics has become available for ART, most recently, potent entry and maturation inhibitors.^11^^,^^12^ These versatile antiretrovirals (ARVs) remain essential to ensure uninterrupted, lifelong treatment options for HIV-1, providing the ability to switch between regimens as needed due to drug resistance, side effects, and drug-drug interactions.^13^^,^^14^ The success of ART has also brought new perspectives to the development of HIV therapeutics. More potent drugs have allowed the reduction of combination therapy from triple therapy to double therapy and in controlled settings even to monotherapy.^15^^,^^16^^,^^17^^,^^18^^,^^19^ The development of drugs with longer half-lives has enabled a shift to once-daily dosing,^20^ with more recent developments toward long-acting injectables creating options for dosing currently every four to eight weeks.^21^^,^^22^ Extending long-acting therapeutic options further, broadly neutralizing antibodies (bnAbs) are considered as the component of pre- and post-exposure prophylaxis, long-term ART, and HIV-1 cure strategies.^23^^,^^24^^,^^25^ In this context, next to passive application of bnAbs, therapeutic bnAb-inducing vaccines are in development.^26^ Combining bnAbs with conventional ART to ascertain full virus suppression is probed for the treatment of infants born to HIV-positive mothers (PedMAb1 trial, Pan African Clinical Trials Registry: PACTR202205715278722).^27^ In PWH on ART, clinical trials are underway for preventive and therapeutic bnAb vaccines (clinicaltrials.gov: NCT05208125, NCT04985760, NCT06006546, and NCT04985760).^28^

While diverse bnAb treatment approaches move to advanced clinical testing in ART-treated PWH, test concepts still need to be established to allow accurate monitoring of bnAb levels and activity side-by-side with drug level monitoring^29^ to enable efficacy evaluation across studies. Specific detection of distinct, passively administered bnAbs is possible, e.g., through binding to anti-idiotypic antibodies.^30^ However, measuring bnAb neutralization activity in the therapeutic setting remains challenging because conventional ART efficiently blocks HIV in neutralization assays. Thus, standardizable neutralization assay systems are needed that exclude interference of co-delivered drugs.

Standardization and quality assessment of HIV-1 plasma neutralization activity has been successfully implemented for HIV-1 vaccine trials using the TZM-bl-based neutralization assay in combination with envelope (Env) HIV-1 reporter pseudoviruses.^31^^,^^32^ Clinical monitoring and efficacy evaluation depend on neutralization assay formats that provide high throughput, high accuracy, low cost, ease of use, and reproducibility. A neutralization assay format combining all these features and distinguishing between drug and antibody inhibition is therefore a critical need for the development of therapeutic vaccines and bnAb therapeutics. To fill this gap, we here explored modifications of the standard TZM-bl neutralization assay to devise a system that reliably records antibody-based neutralization in the presence of antiretrovirals.

## Results

### ART-resistant HIV-1 vectors for distinguishing plasma antibody from antiretroviral drug inhibition

Our study aimed to develop a modified TZM-bl neutralization assay that is not confounded by the presence of ARVs in plasma and provides high comparability to the standard TZM-bl assay.^32^ We refer to this as ART-free neutralization methods. A logical approach to minimize the inhibitory activity of ARVs is to use ART-resistant HIV-1.^33^^,^^34^^,^^35^ Starting with the wild-type (WT) pseudotyping vector (pv), the reporter virus backbone HIV-1 NLlucAM based on HXB2 (referred to here as HIV^pv^-WT),^36^ we created different vector versions carrying known resistance mutations to reverse transcriptase (RT) and integrase (IN) inhibitors.^37^ The effects of PIs were not considered in our vector design because they inhibit the formation of mature virus and thus successive rounds of infection. The single round of infection that pseudoviruses complete during a neutralization assay is therefore unaffected.

**Figure 1:**
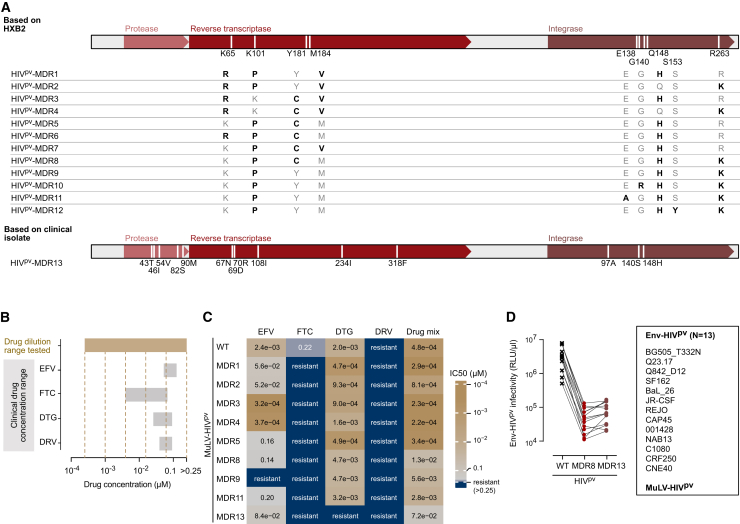
Creating ART-resistant HIV pseudovirus vectors (A) Schematic of the HIV-1 *pol* gene and the combinations of mutations (bold) inserted into the HIV^pv^ backbone to achieve multidrug-resistant (MDR) variants. (B) Schematic overview of plasma drug concentrations tested in (C) and published reference values (see Table S4). Values are adapted to a 1:100 dilution of plasma as used in the neutralization assay. (C) Inhibition of MuLV-pseudotyped HIV^pv^-WT and HIV^pv^-MDR viruses by different ARVs at concentrations depicted in (B). Heatmap depicts mean inhibitory concentration 50 (IC50) values from two independent experiments. Drug mix: efavirenz (EFV), emtricitabine (FTC), dolutegravir (DTG), and darunavir (DRV) were combined and titrated. (D) Infection of TZM-bl cells with serial dilutions of HIV^pv^-WT, HIV^pv^-MDR8, or HIV^pv^-MDR13 pseudotyped with HIV-1 Env (*N* = 13) or MuLV. Infectivity is recorded as RLU and normalized to input of each virus stock. Mean RLU/μL titers from two independent experiments are shown. See also Figure S1.

A general difficulty in creating drug-resistant HIV-1 is that mutations in the targeted enzymes commonly impact viral fitness.^38^^,^^39^^,^^40^^,^^41^ This required us to monitor both the efficacy of the introduced mutations in conferring resistance to the drugs and the infectivity of the mutant viruses to ensure that sufficient infection capacity was maintained to allow their use in neutralization assays. We first focused on selected known key drug resistance mutations that were introduced in different combinations to HIV^pv^-WT to create the multidrug-resistant (MDR) variants HIV^pv^-MDR1 to MDR12 (Figure 1A). To reduce the impact of NRTIs, we included mutations K65R and M184V.^42^^,^^43^^,^^44^ For NNRTI resistance, mutations K101P and Y181C^45^ were added. Resistance to INSTIs often results in highly decreased integrase function.^46^^,^^47^ We therefore tested different combinations of mutations to obtain resistance to INSTIs while maintaining sufficient integrase activity, including R263K, a mutation conferring low-level resistance to the second-generation INSTI dolutegravir (DTG),^48^ and/or Q148H, a mutation associated with high resistance to first-generation INSTIs.^49^^,^^50^ Mutations E138A, G140R, or S153Y were combined with the two major drug resistance mutations R263K and Q148H to achieve resistance against second-generation INSTIs including DTG and cabotegravir.^51^^,^^52^ Next to these designed mutants, we cloned the *pol* gene of an HIV-1 isolate identified in routine clinical diagnostics with clinically documented resistance to four drug classes (NRTI, NNRTI, PI, and INSTI) into HIV^pv^ (HIV^pv^-MDR13; Figure 1A).

As expected, there was a decrease in infectivity on TZM-bl cells compared to HIV^pv^-WT for all MDR viruses tested (1.2–2.9 log fold loss in infectivity; Figures S1A and S1B). Constructs HIV^pv^-MDR6, -MDR7, -MDR10, and -MDR12 proved not vital (Figures S1A and S1B). The remaining nine HIV^pv^-MDR viruses showed sufficient infectivity and were compared to HIV^pv^-WT for resistance to ARVs (Figures 1B and 1C). In this screen, the different pv backbones were examined as MuLV pseudotypes to analyze the inhibitory activity of ARVs. As only the Env molecule is derived from MuLV, all steps of the replication cycle, except for entry, are the same as with HIV-Env pseudoviruses. Therefore, the MuLV screening detects inhibition by most ARVs except HIV entry inhibitors. As representative drugs for the different inhibitor classes, the following ARVs were included in the screening: efavirenz (EFV) for NNRTI,^53^^,^^54^ emtricitabine (FTC) for NRTI,^55^^,^^56^ DTG as INSTI,^57^^,^^58^ and darunavir (DRV) as PI.^59^^,^^60^ Inhibitor dosing in these *in vitro* tests was adjusted to reflect the range of clinical drug levels reported in the literature (Figure 1B).^61^^,^^62^^,^^63^^,^^64^

The PI darunavir was included as non-active control in the HIV^pv^ system. It showed the expected lack of inhibitory activity against HIV^pv^-WT over the full concentration range tested (Figures 1C, S1C, and S1D). Emtricitabine did not affect HIV^pv^ infectivity in dose ranges that are relevant for plasma neutralization assays (Figures 1C and S1C) and reached >50% inhibition of HIV^pv^-WT only at concentrations >0.25 μM (Figure S1D). Resistance to EFV was achieved through mutations in the RT with K101P alone or in combination with Y181C. As described for *in vitro* DTG susceptibility,^65^ R263K paired with Q148H partially reduced the inhibitory activity of DTG at clinically relevant doses (Figures 1C and S1C).

To mimic combination ART, we generated a drug cocktail containing all four drugs and probed it against the MDR viruses (Figures 1C and S1C). Among the designed HIV^pv^-MDRs, HIV^pv^-MDR8, carrying two RT mutations (K101P and Y181C) and two IN mutations (R263K and Q184H), was the best in conferring resistance to the cocktail. Notably, the clinical isolate-derived HIV^pv^-MDR13 reached complete resistance against DTG and the highest overall resistance to the drug mix. Based on this, HIV^pv^-MDR8 and -MDR13 were rated as the most promising candidates to follow up as ART-resistant pseudotyping vectors.

To probe the utility of HIV^pv^-MDR8 and MDR13 for neutralization assays, we verified if their infectivity is sufficient for TZM-bl-based neutralization tests. For this, we generated pseudotypes carrying Env of MuLV and diverse HIV-1 strains (*N* = 13) and compared their infectivity on TZM-bl cells with HIV^pv^-WT (Figure 1D; Table S1). Across all viruses, HIV^pv^-MDR8 and -MDR13 pseudoviruses showed 1.8 log (SD 0.22) and 1.6 log (SD 0.15) reduced infectivity, respectively. Although low, all pseudoviruses except one, the CAP45 pseudovirus with the HIV^pv^-MDR8 backbone, resulted in luciferase reporter production greater than 10-fold above background as recommended by the standard TZM-bl neutralization protocol (Table S1).^32^ CAP45 pseudovirus achieved luciferase levels only 9-fold above background but provided consistent NT50 data across duplicate measurements and was therefore included in the analysis.

Overall, utilizing ART-resistant HIV pseudovectors for neutralization assays proved feasible with some limitations, as fitness losses, in particular for multidrug resistance, can be substantial and none of the constructs reached full resistance.

### Limitations of assessing HIV-1 antibody neutralization with the VSV pseudovirus system

**Figure 2:**
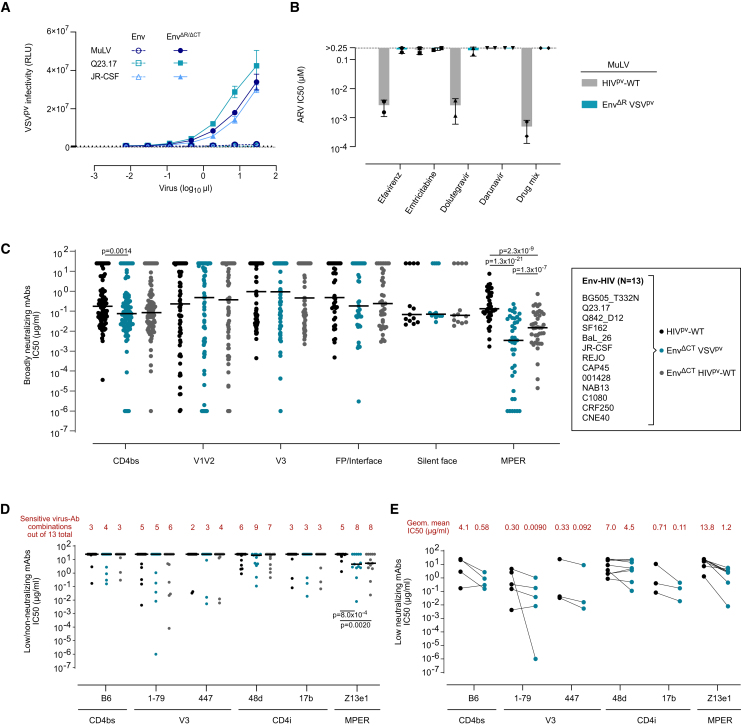
Differential sensitivity of VSV-based pseudoviruses to antibody neutralization (A) Infectivity of VSV^pv^ viruses pseudotyped with full-length (open symbols) or CT-truncated (ΔCT) HIV-1 Env/R peptide-deleted (ΔR) MuLV (closed symbols). Infectivity of virus titrations was measured on TZM-bl cells and recorded as RLUs. Error bars indicate the standard deviation. (B) Sensitivity of MuLV-pseudotyped VSV^pv^ viruses (blue) to ARVs with ARV concentrations as shown in Figure 1B. IC50 values of two independent experiments in a TZM-bl-based neutralization assay are shown. Error bars indicate the standard deviation. Data for HIV^pv^-WT (black) from Figure 1C are depicted for comparison. (C) Neutralization of a 13-virus panel in the context of HIV^pv^-WT (black), Env^ΔCT^ VSV^pv^ (blue), and Env^ΔCT^ HIV^pv^-WT (gray) by a panel of 30 bnAbs. Mean IC50 values of two independent experiments are shown. Significance thresholds are adjusted for multiple testing and indicated as follows: ∗*p* < 0.05/18, ∗∗*p* < 0.01/18, ∗∗∗*p* < 0.001/18. (D) Sensitivity of the different pseudoviruses to six low/non-neutralizing mAbs. Mean IC50 values of two independent experiments are shown. Significance thresholds are adjusted on multiple testing and indicated as follows: ∗*p* < 0.05/12, ∗∗*p* < 0.01/12, ∗∗∗*p* < 0.001/12. (E) IC50 comparison of VSV^pv^-sensitive low/non-neutralizing mAb/virus combinations with HIV^pv^-WT as depicted in (D). See also Figure S2 and Table S3.

We next considered pseudoviruses based on vesicular stomatitis virus (VSV) as a tool to prevent interference of ART in neutralization assays. VSV-based pseudoviruses have been used to study the entry and neutralization of various viruses, most recently severe acute respiratory syndrome coronavirus 2.^66^^,^^67^^,^^68^^,^^69^^,^^70^ VSV, an enveloped single-stranded RNA virus of the *Rhabdoviridae* family, is completely distinct from HIV, both genetically and in terms of its replication cycle, rendering VSV intrinsically resistant to HIV-1 ARVs. As described previously, to generate entry-competent HIV-1 Env VSV pseudoviruses (VSV^pv^), the cytoplasmic tail (CT) of Env must be deleted to allow Env incorporation into VSV particles (Figure 2A).^71^ In analogy, the R peptide of MuLV Env^72^^,^^73^ was deleted to produce infectious viral particles. Probing a panel of Env^ΔCT^ VSV^pv^ (HIV-1 Env^ΔCT^ (*N* = 13) and MuLV Env^ΔR^; Table S1), we confirmed that VSV pseudoviruses show no sensitivity against the tested ARVs (Figures 2B and S2A).

Considering that incorporation into a different virus particle and/or CT truncation may affect HIV-1 Env trimer conformation^74^^,^^75^^,^^76^ and presentation of neutralizing epitopes, we next examined the sensitivity of the VSV^pv^ panel (*N* = 13) against a range of neutralizing monoclonal antibodies (mAbs; *N* = 30) and compared the sensitivity to matched HIV^pv^-WT data (Figures 2C and S2B–S2D; Tables S2 and S3). For most mAbs, neutralization activity was similar in both virus systems, but some mAb-VSV^pv^ combinations stood out as more sensitive. We noted a generally higher sensitivity of VSV^pv^ for mAbs targeting the CD4-binding site (CD4bs) and the membrane proximal external region (MPER) (Figures 2C and S2B–S2D). The most pronounced increase in sensitivity was observed for the tested MPER bnAbs 10E8, 4E10, and DH511.11P with 10E8 reaching a median 1,698-fold lower IC50 against the VSV^pv^ panel (Figure S2C). CD4bs bnAbs VRC01, b12, and CH235.12 but not N6, 1–18, PG04, and N49P7 were also highly affected by an increased sensitivity to VSV^pv^ (Figure S2D).

An increased neutralization sensitivity inflicted by the assay system is evidently of concern. We thus investigated if probing neutralization activity with VSV^pv^ would extend to classify low or non-neutralizing mAbs falsely as neutralizing. Comparing HIV^pv^-WT with VSV^pv^ for six low/non-neutralizing mAbs (Table S2), we noted indeed some shifts toward sensitivity (Figure 2D). Overall, 8/54 virus-antibody combinations shifted from resistance to detectable neutralization when tested as HIV^pv^-WT (IC50 > 25 μg/mL; Table S3). Additionally, some virus-antibody combinations that were already sensitive as HIV^pv^-WT showed increased sensitivity in the context of VSV^pv^ (Figure 2E). Except for mAb 1–79 in combination with virus NAB13, the gain in potency was the highest for the MPER mAb Z13e1, mirroring what we observed for bnAbs and suggesting that MPER mAbs benefit from improved access to their epitope on Env^ΔCT^ VSV^pv^. To evaluate if the increased sensitivity was caused by the truncation of the CT or the VSV-based vector itself, we probed Env^ΔCT^^-^pseudotyped viruses in the context of HIV^pv^-WT (Figures 2C, 2D, and S2B–S2D). Overall, Env^ΔCT^ HIV^pv^-WT showed the same trend toward increased neutralization sensitivity confirming the CT as a critical component in steering neutralization sensitivity for certain epitopes.^74^^,^^75^^,^^76^ Taken together, our analysis suggests that VSV pseudoviruses may overestimate the neutralizing activity of certain antibody types and should be used with caution.

### Low recovery of immunoglobulins after protein A/G bead separation

**Figure 3:**
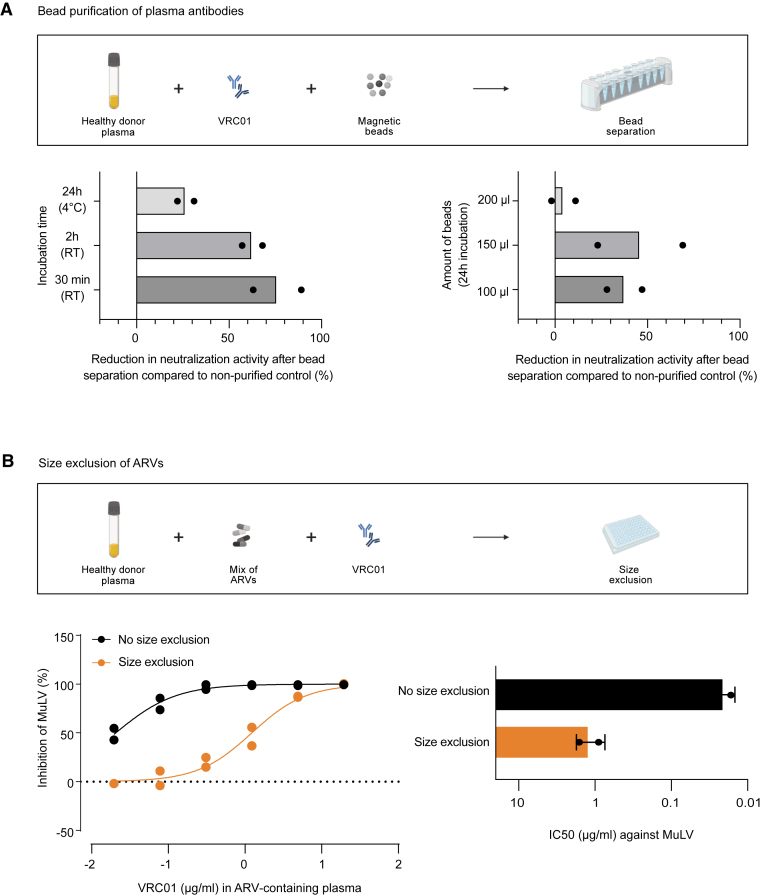
Strategies to separate ARVs from plasma antibodies (A) Antibody separation by protein A/G beads: VRC01 (250 μg/mL) spiked into 10 μL healthy donor plasma was incubated with protein A/G magnetic beads before antibody elution. Neutralization activity against Q23.17 of the antibody-containing fraction was assessed in a HIV^pv^-WT neutralization assay, and reduction of neutralization activity compared to untreated VRC01-spiked plasma was calculated. A fixed amount of beads (100 μL corresponding to 3,000 μg) was incubated at different times and temperatures (left) or varying amounts of beads were incubated for 24 h at 4°C before antibody elution (right). (B) Size exclusion to separate ARVs from plasma: VRC01 (500 μg/mL) and the drug mix (EFV/FTC/DTG/DRV) were spiked into healthy donor plasma and size exclusion was performed. Inhibition of MuLV-pseudotyped HIV^pv^-WT to reflect inhibition by ARVs was analyzed before and after size exclusion. MuLV inhibition curve (left) and corresponding IC50 values relating to VRC01 content (right) from two independent experiments are depicted. Error bars indicate the standard deviation. See also Figure S3.

We next evaluated the purification of plasma immunoglobulins (Ig) using protein A/G^77^^,^^78^^,^^79^ to separate antibodies from ARVs. For this, antibodies need to be separated with minimal loss to allow an accurate assessment of plasma neutralization activity. We therefore first assessed and optimized the Ig isolation procedure (Figures 3A and S3A–S3C). To this end, we prepared healthy donor plasma spiked with the CD4bs bnAb VRC01 (250 μg/mL in plasma) and tested its neutralization activity against Q23.17 HIV^pv^-WT before and after Ig purification with protein A/G magnetic beads. For this, plasma and purified polyclonal antibodies were reconstituted to the same volume to allow direct comparison. Using a bead dose of 100 μL beads (50 μL protein A and 50 μL protein G) per 10 μL plasma and an incubation time of 30 min at room temperature, we observed a drastic loss in VRC01 neutralization activity (76.0% loss in neutralization activity [SD 18.4%] compared to untreated sample; Figure 3A). By elongating the incubation time to 24 h and increasing the amount of magnetic beads to 200 μL beads per 10 μL plasma (Figure 3A), the loss of neutralization activity was reduced from 76.0% (SD 18.4%) to 4.5% (SD 9.2%). However, while we found that protein A/G purification completely removes ARVs (Figure S3D), we consider the approach challenging for most high-throughput screens due to the hands-on time required, the large amount of beads, and their significant cost. Additionally, application of the method for clinical monitoring of antibody activity may require measuring of Ig yields as recovery across Ig subtypes may differ.

### ART-DEX efficiently removes antiretrovirals from plasma

We next sought to implement a strategy that removes ARVs from plasma, rather than purifying antibodies. Current routinely used ARVs are small molecules with molecular weight (MW) of around 200–800 Da. Considering the larger size of Ig (MW∼150 kDa), a straightforward separation by size exclusion would be in principle possible for ARVs except for antibody- or peptide-based inhibitors (e.g., ibalizumab or enfuvirtide as entry inhibitors).^11^^,^^80^ However, most ARVs, in particular NNRTIs and PIs, are highly bound to plasma proteins and thus may not separate well by size exclusion (Table S4).^81^^,^^82^^,^^83^ We probed this by spiking ARVs (EFV, FTC, DTG, and DRV) and VRC01 (individually or in combination with ARVs) into healthy donor plasma and performing size exclusion using commercial 96-well spin plates with a MW cutoff of 40 kDa. To monitor solely the effects of ARV inhibition, MuLV HIV^pv^-WT was investigated, and to monitor effects on antibodies, VRC01 activity against Q23.17 HIV^pv^-WT was measured. Although ARV activity in the spiked plasma against MuLV was reduced by 1.76 log (SD 0.043) after size exclusion, the residual drug activity was still substantial (Figure 3B).

**Figure 4:**
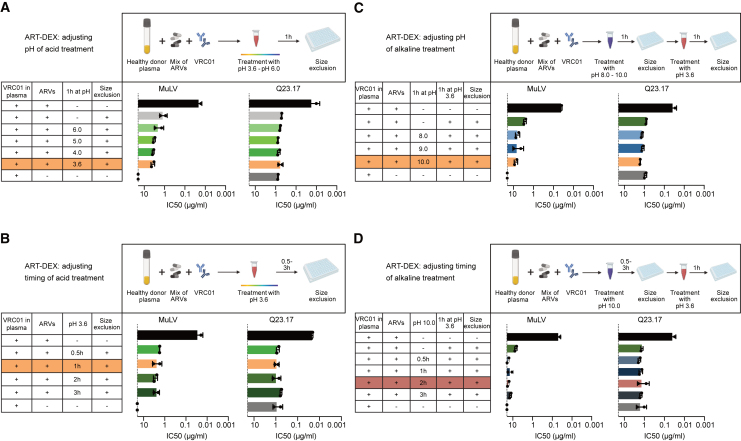
Dissociation and size exclusion to release and separate ARVs from plasma proteins (A–D) Optimizing conditions for pH-dependent ARV dissociation from plasma proteins prior to size exclusion. Healthy donor plasma spiked with VRC01 (500 μg/mL) and the drug mix (EFV/FTC/DTG/DRV) was subjected to acidic (A and B) or combined alkaline/acidic (C and D) treatment before proceeding with size exclusion using spin plates. Optimal pH (A and C) and incubation times (B and D) were defined by testing separated antibody-containing plasma fractions for inhibition of MuLV and Q23.17-pseudotyped HIV^pv^-WT. IC50 titers relate to VRC01 content. Data are from two independent experiments for each condition. Error bars indicate the standard deviation. In each optimization round shown in (A–C), the condition that was selected to proceed is highlighted in orange. The red shaded box in (D) indicates the final conditions of dissociation and size exclusion (ART-DEX) that were used thereafter (pH 10 for 2 h/size exclusion/pH 3.6 for 1 h/size exclusion). See also Figure S3.

We next made use of the fact that protein binding of drugs can be pH dependent.^84^ We first investigated the effect of acidic pH in releasing ARVs from plasma proteins. Plasma samples were incubated in buffer solutions with pH ranging from 3.6 to 6.0 for 1 h followed by size-exclusion separation (Figure 4A). Incubation at pH to 3.6 for 1 h reduced ARV activity by an additional 0.51 log (SD 0.27) compared to size exclusion alone. Notably, the inhibitory activity of VRC01 was not affected by the pH treatment as the plasma spiked solely with VRC01 yielded similar neutralization activity against Q23.17 as after acid treatment and size exclusion (Figure 4A). Longer incubation at pH 3.6 did not further reduce the inhibitory activity of ARVs (Figure 4B).

To combat the residual ART inhibition after acidic treatment and size exclusion, we next considered an additional alkaline treatment as pH dependency may vary dependent on the plasma protein bound. Initial experiments showed that an alkaline step prior to the addition of plasma to TZM-bl cells should be avoided, as it may, at least at high doses of the buffer, impact neutralization assays and cell viability (Figures S3E and S3F). We therefore opted to first incubate plasma in alkaline pH followed by a size-exclusion step and then proceed with incubation at pH 3.6, again followed by size exclusion. Adding an alkaline treatment for 2 h at pH 10.0 was the most effective and led to a further decrease of ARV activity by 0.45 log (SD 0.018) compared to the acid treatment and size exclusion (Figure 4C). Neutralization capacity of VRC01 against Q23.17 was not affected. Incubation for more than 2 h at pH 10.0 did not further reduce the inhibitory activity of ARVs (Figure 4D).

We refer to this combined approach as ART dissociation and size exclusion (ART-DEX). We found that the final ART-DEX protocol—with combined alkaline (2 h at pH 10) and acid (1 h at pH 3.6) treatment with size-exclusion steps after each pH treatment—reduced the inhibitory activity of the tested mix of ARVs by 2.54 log (SD 0.17) resulting only in low-level residual ART inhibition of MuLV (IC50 = 17.22 μg/mL [SD 2.14 μg/mL], cutoff = 25 μg/mL). Considering that we probed comparatively high levels of drugs that represent maximal plasma concentrations in clinical use, the majority of ARV influence in plasma samples can be eliminated with this approach. As the procedure requires comparatively little handling and allows quantitative retrieval of antibody activity at lower costs than Ig purification, it also holds promise for high-throughput applications.

### Combining ART-DEX with ART-resistant HIV pseudovirus

**Figure 5:**
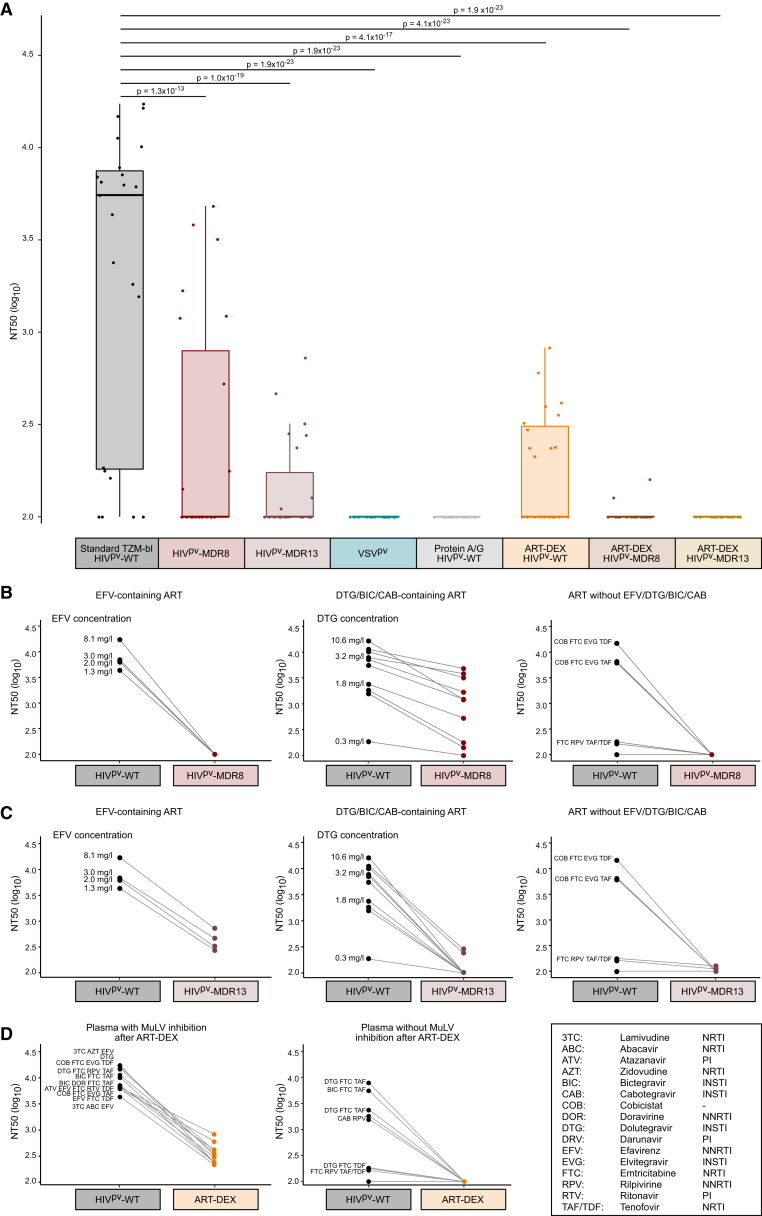
Verifying the capacity of ARV removal strategies in plasma of ART-treated PWH (A) Plasma samples from PWH (*N* = 23) with different ART combination regimen were tested for MuLV inhibition using the standard TZM-bl assay or the indicated ART-free strategies. The effective primary plasma dilution during culture was 1:100 (after combination of plasma, pseudovirus, and TZM-bl cells). NT50 titer against MuLV derived from two independent experiments is shown. Boxplots represent median with the middle line, upper and lower quartiles with the box limits, and 1.5 x interquartile ranges with the whiskers. Significance thresholds are adjusted for multiple testing and indicated as follows: ∗*p* < 0.05/7, ∗∗*p* < 0.01/7, ∗∗∗*p* < 0.001/7. (B and C) Dissecting resistance of HIV^pv^-MDR8 (B) and HIV^pv^-MDR13 (C) to different ART regimen in NT50 data depicted in (A). Clinically determined drug concentrations for efavirenz and dolutegravir are indicated. (D) Comparison of drug regimens with and without residual ARV inhibition following ART-DEX as depicted in (A). See also Figure S4, Table S5.

In total, we established four different ART-free neutralization protocols to eliminate the influence of ARVs on antibody neutralization, two protocols based on resistant pseudovectors and two methods that attempt to separate ARVs from antibodies. We next compared the four approaches on plasma of PWH (*N* = 23) who were treated with different ARV combinations and for whom plasma drug level measurements were available through the biobank of the Zurich Primary HIV Infection study (ZPHI; Table S5).^85^ In selecting these plasma samples, we aimed to investigate common drug combinations and sought to include also those that contained a second-generation INSTI in the drug regimen. The initial plasma dilution was set to 1:100. Neutralization titers against MuLV above 100 were defined as residual inhibitory activity of ARVs. Without treatment, 82.6% (19/23) of the samples inhibited MuLV in the standard TZM-bl/HIV^pv^-WT assay at a median NT50 titer of 6,291 (Figure 5A). Both, protein A/G purification and VSV-based pseudoviruses, completely abolished ARV activity against MuLV as expected. HIV^pv^-MDR8 pseudoviruses reduced activity against MuLV, but 39.1% (9/23) of the samples still showed residual MuLV inhibition. Analysis of the drug combinations with and without activity against MuLV HIV^pv^-MDR8 indicated a full resistance to EFV (Figure 5B, left panel). Activity of ARV combinations containing second-generation INSTIs, DTG, bictegravir, or long-acting cabotegravir showed reduced activity compared to HIV^pv^-WT, but still retained considerable residual inhibitory activity with a median NT50 titer of 1,209 (Figure 5B, middle panel). None of the other tested drug combinations inhibited MuLV HIV^pv^-MDR8 (Figure 5B, right panel) consistent with the results obtained with single drugs spiked into healthy donor plasma (Figure 1C). HIV^pv^-MDR13 outperformed -MDR8, with residual MuLV inhibition in 34.8% (8/23) samples with a median NT50 of 280 (Figure 5A). HIV^pv^-MDR13 substantially reduced NT50 titers for ARV combinations containing EFV (median NT50 = 393) but also for second-generation INSTIs (Figure 5C). Using ART-DEX, 47.8% (11/23) of samples retained residual inhibition of MuLV, albeit at low NT50 titers with a median of 322 (Figure 5A). Analysis of drug combinations with residual MuLV inhibition after ART-DEX (Figure 5D) suggested that EFV, DTG, bictegravir, and elvitegravir were not fully removed.

To resolve this residual ARV inhibition, we next combined ART-DEX with HIV^pv^-MDR8 and HIV^pv^-MDR13. The combination with HIV^pv^-MDR13 completely eliminated the inhibitory activity of ARVs in all tested ZPHI plasma samples (*N* = 23) including those that contained high concentrations of second-generation INSTIs (Figure 5A). For an in-depth evaluation of the ability of the ART-DEX method to remove ARVs, we expanded the analysis to a total of 563 plasma samples from PWH on ART (Figure S4A; Table S5). We typically see a range of activity against MuLV in the standard TZM-bl assay with HIV^pv^-WT, reaching from no inhibition at a plasma dilution of 1:100 to relatively high titers (>10,000). Among the probed plasma samples, 359/563 (63.8%) recorded with MuLV HIV^pv^-WT inhibition. We subjected 108 of these samples with MuLV inhibition to ART-DEX in combination with HIV^pv^-MDR13 (ART-DEX/HIV^pv^-MDR13; Figure S4B). For 91.7% (99/108), ARV removal by ART-DEX with HIV^pv^-MDR13 was successful. Neutralization activity measured against SF162 for plasma samples without MuLV inhibition after ART-DEX/HIV^pv^-MDR13 (*N* = 80) showed activity in the range expected for this tier 1A HIV-1 strain (median NT50 = 321; Figure S4C).

Of note, during our study, the manufacturer released an updated version of the size-exclusion plates that required some protocol adaptations. We therefore verified the ART-DEX protocol for the updated plate version and confirmed the performance of ART removal from the ZPHI plasmas (Figure S4D). Results with the updated plate version yielded essentially the same results as the original plates, with 30.4% (7/23) of samples showing residual MuLV inhibition after ART-DEX at a median NT50 titer of 794 and the combination of ART-DEX and HIV^pv^-MDR13 resulting in complete elimination of ARV inhibitory activity. In conclusion, we rate the combination of ART-DEX with HIV^pv^-MDR13 as the most effective approach to distinguish antibody-based neutralization from drug inhibition.

### ART-DEX combined with HIV^pv^-MDR13 accurately captures plasma antibody neutralization

**Figure 6:**
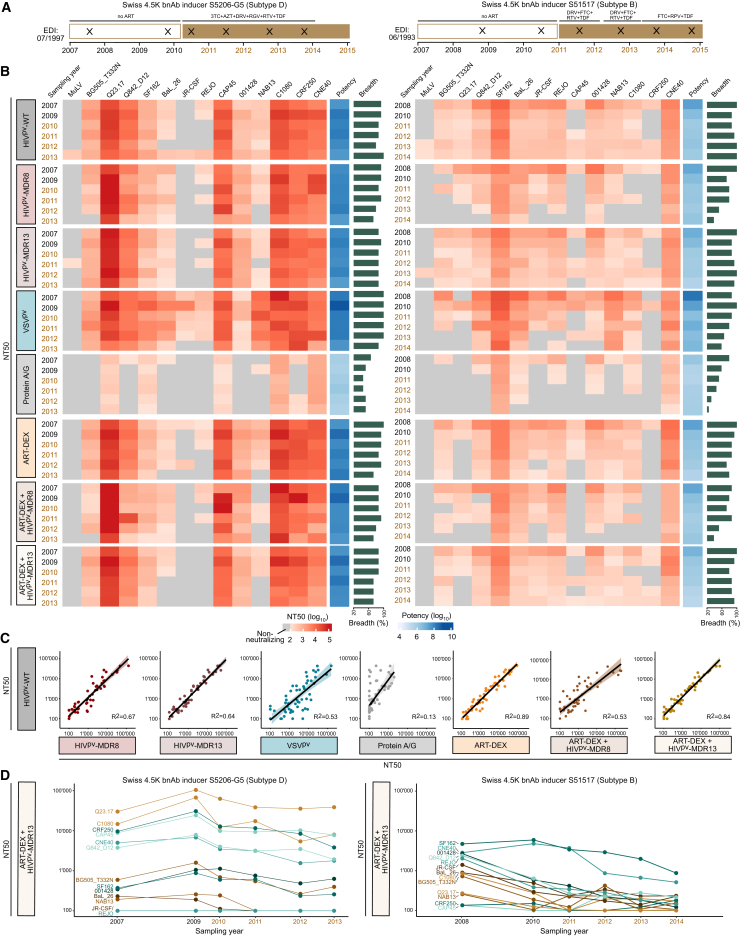
ART-DEX combined with HIV^pv^-MDR13 ranks best in monitoring *in vivo* neutralization capacity (A) Two bnAb inducers were assessed for neutralization activity over several years of follow-up, each encompassing two time points without ART (clear boxes) and four time points with ART (brown boxes). EDIs (estimated date of infection) and specific ART combinations at sampling time points (crosses) are listed. (B) Plasma neutralization activity of bnAb inducer S5206-G5 (left) and S51517 (right) was assessed with a 13-virus panel and MuLV as control using the different ART-free neutralization procedures and the standard TZM-bl assay. The effective primary plasma dilution during culture was 1:100 (after combination of plasma, pseudovirus, and TZM-bl cells). Heatmaps of the respective NT50 titers for each virus/plasma/method combination with potency (geometric mean NT50) and breadth (%) across HIV pseudoviruses are depicted. (C) Correlation analysis of NT50 titers based on HIV^pv^-WT and the ART-free neutralization methods using solely data from the two sampling time points before ART initiation. (D) NT50 titers against the 13-virus panel for ART-DEX/HIV^pv^-MDR13 (see B) are depicted as line plots over time. See also Figure S5 and Table S6.

We next explored which of the ART-free neutralization methods most accurately reflects plasma neutralization activity measured in the standard TZM-bl/HIV^pv^-WT assay. To this end, we utilized longitudinal plasma samples from two bnAb inducers, S5206-G5 and S51517, identified in the Swiss 4.5K screen^86^^,^^87^ (Figure 6A) and assessed their neutralization capacity against a 14-virus panel (Table S1) with the different ART-free neutralization methods using the original version of the spin plates (Figure 6B; Table S6). For each bnAb inducer, six plasma samples were tested, two from off-ART and four from on-ART time points. Off-ART samples from S5206-G5 were from an ART interruption phase. Having first established the ability of the different methods to remove ARVs from plasma (Figure 5), we next focused on examining the accuracy of the methods in capturing plasma neutralization capacity equivalent to the standard TZM-bl assay. For this purpose, we selected plasma samples in which the influence of ARVs on the standard TZM-bl neutralization assay was low: MuLV HIV^pv^-WT was inhibited by only a fraction of the longitudinal on-ART time points tested for donor S5206-G5 (1/4) and donor S51517 (2/4), and then at low titers. This overall low effect of ARVs over the longitudinal plasma time course allowed direct comparison of the ART-free neutralization methods with the neutralization titers obtained by the standard TZM-bl neutralization assay, providing a means to assess the quality of the evaluated ART-free techniques for accurately recording bnAb activity. All seven strategies (the five individual methods and the combined ART-DEX with HIV^pv^-MDR8 or HIV^pv^-MDR13) eliminated the ART influence. Despite using the optimized bead protocol, protein A/G purification was surprisingly poor and led to a massive loss in neutralization activity. All other methods captured the neutralization activity well (Figure 6B; Table S6). Notably, VSV^pv^ showed an enhanced neutralization sensitivity, which was particularly striking for JR-CSF (Figure S5A). Modest increases in neutralization sensitivity were also notable for HIV^pv^-MDR8 alone or in combination with ART-DEX, but these were not uniform and restricted to few plasma-virus combinations. Overall, the variation in neutralization titers compared to the standard TZM-bl assay was within the expected range of assay variation and only showed notable differences for protein A/G purification and VSV^pv^-based assays (Figure S5B).

Linear regression analysis considering only samples off-ART underlined the differential performance of the ART-free methods and signified ART-DEX as the method most accurately capturing neutralization activity as recorded by the standard method reaching R^2^ = 0.89 (Figures 6C and S5C). Both, protein A/G purification (R^2^ = 0.13) due to the massive loss in activity and VSV^pv^ (R^2^ = 0.53) due to the potential shifts in neutralization for certain antibody types, need to be used with caution. Approaches with HIV^pv^-MDR8 or HIV^pv^-MDR13 alone (R^2^ = 0.67 and R^2^ = 0.64, respectively) or ART-DEX/HIV^pv^-MDR8 (R^2^ = 0.53) performed well but did not reach the level of agreement seen with ART-DEX alone or ART-DEX/HIV^pv^-MDR13 (R^2^ = 0.84). We again verified that the updated version of the spin plates showed the same performance (Figures S5D and S5E).

Due to the high correlation with the standard TZM-bl assay (Figures 6C and S5C) as well as the complete removal of inhibitory activity of ARVs (Figure 5A), we ranked ART-DEX/HIV^pv^-MDR13 as the best-performing ART-free method that allows accurate longitudinal assessment of antibody-based neutralization in PWH on ART (Figure 6D).

## Discussion

Technical solutions that permit monitoring of antibody neutralization in ART-containing plasma will be a critical component to move bnAb therapeutics forward and establish their clinical use beyond carefully controlled experimental studies with analytical treatment interruption (ATI).^88^^,^^89^^,^^90^ Measuring bnAb activity in HIV cure applications will be particularly important to ensure that only individuals who have responded sufficiently to bnAb treatment or vaccination undergo ATI.

Here, we aimed to develop strategies to monitor antibody-based inhibition in plasma with HIV enzyme-targeting ARVs. We sought methods that reliably quantify HIV antibody neutralization, are scalable for higher throughput, and cost-effective. We pursued four strategies, focusing on ART-resistant viral vectors or separating ARVs from plasma.

Establishing ARV-resistant viruses for neutralizing assays is attractive because, if successful, this approach will allow direct testing of ARV-containing plasma for neutralizing antibody activity using the conventional TZM-bl assay with minimal modification and no additional plasma processing. However, both pseudovirus systems we studied, the ART-resistant HIV^pv^ and VSV^pv^, showed limitations. Achieving full resistance to HIV enzyme-targeting ARVs while maintaining sufficient infection capacity of *pol*-mutated HIV^pv^ proved to be challenging. In creating suitable multidrug-resistant HIV^pv^, we thus aimed for a compromise between sufficient infectivity at the expense of residual ART sensitivity. This balance was best achieved for two constructs, one engineered to harbor selected drug resistance mutations and the other containing the entire *pol* gene of a multidrug-resistant HIV isolate discovered in routine resistance testing. Both showed good but not complete resistance to all categories of ARVs tested. Therefore, their use should be determined based on the specific ART context of the study population. With increasing options of more potent drugs arising, multi-resistant HIV pseudovectors will need continuous adaptation. Realistically, given the decrease in infectivity observed with each added resistance mutation, the chances of creating a single multi-resistant HIV backbone that confers resistance to all major ARVs must be considered limited.

While VSV^pv^ is inherently resistant to HIV-specific ARVs, the drawback of this system lies in alterations in HIV-1 Env conformation in the context of VSV particles that induced shifts in antibody-based neutralization particularly noticeable for MPER- and CD4bs-directed mAbs. These shifts were linked to truncation of the Env CT needed for sufficient HIV Env incorporation into VSV. This truncation can reduce trimer stability, alternate epitope exposure, and/or induce changes in fusion kinetics,^91^^,^^92^^,^^93^ which may be particularly advantageous for epitopes such as the MPER domain, which has known low accessibility.^94^^,^^95^ Unilateral shifts in neutralization activity, as we observed for VSV pseudovirus neutralization with plasma samples from bnAb inducers, are particularly problematic to control for when assessing polyclonal responses. Therefore, VSV-based systems should be used with caution.

To separate ARVs from antibodies in plasma, we first assessed Ig purification using protein A/G separation, a method that has been widely used.^77^^,^^78^^,^^79^ As others, we found protein A/G purification highly efficient in separating antibodies from ARVs.^77^^,^^96^^,^^97^ In our setup, this was however accompanied by a marked loss in antibody recovery. As we showed, increasing protein A/G bead concentration can improve antibody recovery from plasma but will increase costs substantially. Protein A/G protocols with higher antibody binding capacity than we observed here can probably be developed. However, given the results of the present study, the potential for low recovery needs to be controlled. This requires that Ig concentrations before and after the purification be measured, and fixed antibody doses be used for neutralization assays.^96^ Overall, we consider antibody purification as highly effective in separating antibodies from ARVs but challenging for high-throughput testing and clinical monitoring due to sample handling and the need to control for Ig recovery.

When purifying Ig from plasma, it is important to consider that the recovery of antibody subtypes may vary. Purified Ig may have a different composition than unprocessed plasma, which could affect the measurement of neutralization activity. In the final approach, ART-DEX, we therefore developed a strategy to remove ARVs from plasma while leaving the plasma antibodies untouched. This approach required the dissociation of ARVs from plasma proteins to allow the removal of the free drugs by size-exclusion separation. ARVs, especially NNRTIs and PIs, are highly bound to plasma proteins such as albumin or α1-acid glycoprotein.^81^^,^^98^^,^^99^ Notably, drug-protein binding has been shown to be pH dependent^84^ and may be caused by differential ionization of the drugs affecting binding,^100^ pH-dependent changes in affinity,^101^^,^^102^ or pH-dependent conformational changes of the plasma protein affecting the accessibility of binding sites for the drugs.^103^^,^^104^^,^^105^ We exploited this pH dependence to release drugs from plasma proteins by subjecting plasma samples to alkaline and acidic pH. Size-exclusion separation following pH treatment successfully reduced the ARV burden, with only the most potent drugs showing residual inhibition. Given the effect of both alkaline and acidic pH treatment on ARV release from plasma proteins observed during ART-DEX, a systematic evaluation of which drugs are preferentially released at low or high pH would be of interest for future ART-DEX studies. Acid treatment, which is part of ART-DEX, is a common strategy to dissociate immune complexes.^106^^,^^107^^,^^108^ We envision the utility of ART-DEX primarily for specimen on ART where, in the absence of viral replication, immune complex formation with Env antigens will not occur. However, HIV antibodies are known to cross-react with a number of autoantigens, and the effect of ART-DEX on these immune complexes must be considered. Importantly, ART-DEX processing did not affect neutralizing antibody activity. This was demonstrated for neutralization by VRC01 and four polyclonal plasma samples from bnAb inducers off-ART analyzed with and without the ART-DEX protocol. Therefore, while we cannot rule out the possibility that some neutralization enhancing or reducing effects of ART-DEX may be seen when larger numbers of samples or mAbs are analyzed, the overall change in antibody neutralization would be expected to be small. Comparing all methods on the same set of plasma samples collected at time points without ART, ART-DEX treatment yielded the highest agreement with the standard TZM-bl neutralization assay, underlining the validity of using ART-DEX for monitoring neutralization activity. We rate ART-DEX as the most straightforward approach we tested, as neither antibody loss, antibody inactivation, nor distortion of neutralization patterns occur (Table S7).

Collectively, all ART-free strategies we explored in the present study can be used to eliminate ARV influence in HIV neutralization tests when tightly controlled. Their utility will however differ depending on sample size, ARV combination, dosing, and research question studied. Overall, we consider the combined use of drug-resistant HIV pseudoviruses, such as HIV^pv^-MDR13, with ART-DEX of the highest utility for monitoring of HIV antibody neutralization under ART (Table S7). As we demonstrate, the residual ARV activity that may occur in both systems individually is low and can be eliminated when the two approaches are combined (Figure 5; Figure S4). We noted a low-level residual inhibitory activity in some EFV-containing samples after ART-DEX/HIV^pv^-MDR13 (Figure S4B). As this occurred in samples with high ARV inhibition before ART-DEX clean-up, additional clean-up rounds or use of the EFV-resistant HIV^pv^-MDR8 can be considered to resolve this. Overall, ART-DEX/HIV^pv^-MDR13 has a high potential to support clinical monitoring needed to advance therapeutic bnAb applications and vaccines from experimental studies to clinical use. More specifically, ART-DEX protocols can be used not only to screen for bnAb activity in natural HIV infection but also to monitor bnAb activity during therapeutic interventions, including bnAb therapy, cure approaches, or vaccination while on ART. Using the ART-free strategy, bnAb activity can be analyzed more easily prior to ATI and help guide further interventions.

While our study focused on the application of ART-DEX in the context of HIV, ART-DEX is not an HIV-specific approach and can be applied in the assessment of antibody responses concurrent with antiviral treatment in other viral diseases or for other biological tests that require the removal of small drugs from biological fluids.

### Limitations of the study

Our study presents ART-DEX as an effective strategy to overcome antiretroviral interference in measuring HIV-neutralizing antibody activity in the plasma of PWH, but ART-DEX also has limitations that need to be considered. A primary challenge remains that ART-DEX does not fully remove ARVs. Some highly potent ARVs can therefore show residual activity, requiring the combined use of ART-DEX with ART-resistant virus backbones. Future studies should aim to resolve this limitation by installing a second ART-DEX clean-up round. Although we did not observe changes in antibody neutralization after treatment with ART-DEX, it is possible that neutralization enhancing or neutralization reducing effects may only become evident when analyzing larger numbers of samples.

## Consortia

Members of the Swiss HIV Cohort Study are: Irene Alma Abela, Karoline Aebi-Popp, Alexia Anagnostopoulos, Manuel Battegay, Enos Bernasconi, Dominique Laurent Braun, Heiner Bucher, Alexandra Calmy, Matthias Cavassini, Angela Ciuffi, Günter Dollenmaier, Matthias Egger, Luisa Elzi, Jan Fehr, Jacques Fellay, Hansjakob Furrer, Christoph Fux, Günthard Huldrych Fritz, Anna Hachfeld, David Haerry, Barbara Hasse, Hans Hirsch, Matthias Hoffmann, Irene Hösli, Michael Huber, David Jackson-Perry, Christian Kahlert, Laurent Kaiser, Olivia Keiser, Thomas Klimkait, Roger Dimitri Kouyos, Helen Kovari, Katharina Kusejko, Niklaus Labhardt, Karoline Leuzinger, Begogna Martinez de Tejada Catja Marzolini, Karin Jutta Metzner, Nicolas Müller, Johannes Nemeth, Dunja Nicca, Julia Notter, Paolo Paioni, Giuseppe Pantaleo, Matthieu Perreau, Andri Rauch, Luisa Salazar-Vizcaya, Patrick Schmid, Roberto Speck, Marcel Stöckle, Philip Tarr, Alexandra Trkola, Gilles Wandeler, Maja Weisser, and Sabine Yerly.

## STAR★Methods

### Key resources table

**Table:** 

REAGENT or RESOURCE	SOURCE	IDENTIFIER
**Antibodies**		

Monoclonal antibodies, see Table S2	N/A	N/A

**Bacterial and virus strains**		

VSVΔG∗/rLUC	Kerafast	EH1020-PM

**Biological samples**		

Human plasma samples	Swiss HIV Cohort Study, Zurich Primary HIV Infection Study	N/A

**Chemicals, peptides, and recombinant proteins**		

Efavirenz	MedChemExpress	HY-10572
Emtricitabine	MedChemExpress	HY-17427
Dolutegravir	MedChemExpress	HY-13238
Darunavir	MedChemExpress	HY-17040
Dynabeads Protein G for Immunoprecipitation	ThermoFisher Scientific	10003D
Dynabeads Protein A for Immunoprecipitation	ThermoFisher Scientific	10001D

**Critical commercial assays**		

Zeba Spin Desalting Plates, 40K MWCO	ThermoFisher Scientific	A57767
Bright-Glo Luciferase Assay System	Promega	E2650
CellTiter-Glo Luminescent Cell Viability Assay	Promega	G7570
QuikChange II Site-directed mutagenesis kit	Agilent	200523
QuikChange Lightning multi-site directed mutagenesis kit	Agilent	210513
In-Fusion Snap Assembly Master Mix	Takara Bio Inc.	638949

**Experimental models: Cell lines**		

HEK 293-T cells	ATCC	CRL-3216, RRID: CVCL_0063
TZM-bl cells	NIH AIDS Reagent Program	ARP-8129, RRID: CVCL_B478
Expi293F cells	Thermo Fisher Scientific Inc.	A14528

**Oligonucleotides**		

Primers for site-directed mutagenesis, see Table S1	This paper	N/A

**Recombinant DNA**		

pCMV-rev	NIH AIDS Reagent Program	ARP-1443
pNLluc-AM	Provided by A. Marozsan and J. P. Moore (Pugach P. et al.^36^)	N/A
HIV^pv^-MDR13	This paper	N/A
Env plasmids for pseudotyping, see Table S1	N/A	N/A

**Software and algorithms**		

GraphPad Prism version 10.1.0	GraphPad Software	N/A
R version 4.1.0	www.r-project.org	N/A

**Other**		

EnVision 2104 Multilabel Reader	Perkin Elmer	N/A

### Resource availability

#### Lead contact

Further information and requests for resources and reagents should be directed to and will be fulfilled by the lead contact, Alexandra Trkola (trkola.alexandra@virology.uzh.ch).

#### Materials availability

The HIV^pv^-MDR13 vector is available through a standard MTA from the lead contact upon request. The MDR13 *pol* gene is accessible in GenBank (GenBank: PQ066382).

#### Data and code availability

• All data reported in this paper will be shared by the lead contact upon request.
• This paper does not report original code.
• Any additional information required to reanalyze the data reported in this paper is available from the lead contact upon request.

### Experimental model and study participant details

#### Clinical specimen

Plasma samples from people with HIV (PWH) analyzed in this study were provided by the biobanks of the Swiss HIV Cohort study (SHCS)^1^^,^^109^ and the Zurich Primary HIV Infection Study (ZPHI).^85^ The SHCS is registered under the Swiss National Science longitudinal platform (http://www.snf.ch/en/funding/programmes/longitudinal-studies/Pages/default.aspx#Currently%20supported%20longitudinal%20studies). The ZPHI is an ongoing, observational, non-randomized, single center cohort founded in 2002 that specifically enrolls patients with documented acute or recent primary HIV-1 infection (clinicaltrials.gov: NCT00537966). The SHCS and the ZPHI were approved by the ethics committees of the participating institutions (Kantonale Ethikkommission Bern, Ethikkommission des Kantons St. Gallen, Comité Departemental d’Éthique des Spécialités Médicales et de Médicine Communautaire et de Premier Recours, Kantonale Ethikkommission Zürich, Repubblica et Cantone Ticino–Comitato Ethico Cantonale, Commission Cantonale d’Éthique de la Recherche sur l’Être Humain, Ethikkommission beider Basel for the SHCS and Kantonale Ethikkommission Zürich for the ZPHI) and written informed consent was obtained from all participants. Plasma drug concentration data for ZPHI plasma probed in Figure 5 and reference values were available from clinical routine measurements conducted by the clinical chemistry laboratory at the University Hospital Zurich.

#### Cell lines

HEK 293-T cells were obtained from the American Type Culture Collection (ATCC) and TZM-bl reporter cells through the NIH AIDS Reagent Program. Both cell lines were cultured in DMEM containing 10% FCS and antibiotics (Penicillin and Streptomycin), and regularly checked for the absence of mycoplasma. Expi293F cells (Thermo Fisher Scientific Inc., Waltham, USA) were cultured in Expi293 Expression Media (Thermo Fisher Scientific Inc., Waltham, USA).

### Method details

#### Antiretrovirals

Efavirenz, emtricitabine, dolutegravir, and darunavir were obtained from MedChemExpress, Monmouth Junction, USA and working stocks were diluted in PBS. To create the drug mix, 0.25 μM of each, efavirenz (EFV), emtricitabine (FTC), dolutegravir (DTG) and darunavir (DRV) were combined.

#### Antibodies

A full list of mAb sources is provided in Table S2. mAbs 2G12, 4E10, and 447 were obtained from Polymun Scientific GmbH, Austria. All other mAbs were expressed in Expi293F cells by transient transfection using TransIT-PRO transfection reagent (Mirus Bio LLC, Madison, USA) according to the manufacturer’s instructions. Seven days after transfection, supernatants were harvested, centrifuged for 45 min at 2,500 x *g* and sterile filtered. Monoclonal antibodies were purified from supernatants using AmMag Protein A Magnetic Beads (GenScript, Piscataway, USA) according to the manufacturer’s instructions and eluted using glycine (0.1 M, pH 2.7). For buffer exchange, samples were loaded on a Slide-A-Lyzer Dialysis Cassette G2 10K MWCO (Thermo Fisher Scientific Inc., Waltham, USA) according to the manufacturer’s instructions and incubated for 12 h in the desired buffer (10% (w/v) Maltose, 10 mM Tris, 2 mM Acetic acid, pH 4.0 or PBS, pH 7.0). After repetition of the buffer exchange, antibodies were sterile filtered and stored at 4°C.

#### Virus envelope proteins

A full list of wildtype Env proteins used for pseudotyping and their corresponding Genebank entry, clade, and neutralization tier information is provided in Table S1. Cytoplasmic tail-deleted (ΔCT) Env mutants^71^ and MuLV ΔR^72^^,^^73^ were generated by site-directed mutagenesis (QuikChange II Site-directed mutagenesis kit, Agilent, Santa Clara, USA) according to the manufacturer’s instructions using primers listed in Table S1.

#### HIV-1 ART-resistant pseudovector

We routinely use the luciferase reporter HIV vector pNLluc-AM^36^ in TZM-bl-based neutralization assays.^86^ We refer to this vector in the present study as HIV^pv^-WT. Drug-resistant vectors HIV^pv^-MDR1 to 12 (Figure 1A) carrying mutations associated with resistance to NRTIs (K65R, M184V), NNRTIs (K101P, Y181C), and INSTIs (Q148H, R263K, G140R, E138A, S153Y) were generated by cloning the *pol* gene of HXB2 into pUC19 vector (Takara Bio Inc., Japan) using the In-Fusion system (Takara Bio Inc., Japan) according to the manufacturer’s instructions. Mutations were introduced by site-directed mutagenesis (QuikChange Lightning multi-site directed mutagenesis kit, Agilent, Santa Clara, USA) according to the manufacturer’s instructions. Constructs were digested with ApaI and EcoRI (Thermo Fisher Scientific Inc., Waltham, USA) and ligated into NLluc-AM using T4 ligase (Thermo Fisher Scientific Inc., Waltham, USA). All mutations were confirmed by Sanger sequencing (Microsynth AG, Switzerland).

For the clinical isolate-based pseudovirus vector HIV^pv^-MDR13, a plasma sample of a participant of the SHCS^1^ with chronic subtype B HIV infection (>28 years infected) who experienced several drug failures, drug intolerance, and drug toxicities requiring multiple treatment changes over nearly three decades was used. The treatment history comprised the following drugs: nucleos(t)ide treatments (zidovudine, lamivudine, didanosine, zalcitabine, abacavir, tenofovir disoproxil fumarate, tenofovir alafenamide), protease inhibitors (indinavir, nelfinavir, lopinavir/r, atazanavir/r, darunavir/r), non-nucleoside reverse transcriptase inhibitors (delavirdine, efavirenz, rilpivirine, doravirine) and integrase inhibitors (raltegravir, dolutegravir, elvitegravir/c, bictegravir). At the latest treatment failure under bictegravir and doravirine, extensive drug resistance in the protease, reverse transcriptase, and integrase gene was detected by routine genetic resistance testing done by full-length *pol* sequencing. Resistance mutations were detected based on the MinVar pipeline^110^ that uses next-generation sequencing data and information from the Stanford HIV database^111^ to detect drug resistance mutations. RNA was extracted from plasma samples using EMAG (bioMérieux SA, France) and reverse transcribed using the PrimeScript OneStep RT-PCR Kit (Takara Bio Inc., Japan) according to the manufacturer’s instructions. The *pol* gene was amplified using PrimeScript OneStep RT-PCR Kit (Takara Bio Inc., Japan) and the following primers: GCTACAYTAGAAGAAATGATGACAGCAT (forward), GGGGCTTGTTCCATCTATCCTCT/GGGGCTTGTTCCATCTGTCTTCT (reverse). To further amplify the target gene, a nested PCR was performed using Phusion HotStart II DNA Polymerase (Thermo Fisher Scientific Inc., Waltham, USA) and the following primers: GAAGAAATGATGACAGCATGTCAGGGAGT (forward), CCTACCTTGTTATGTCCTGCTTGATA (reverse). The *pol* gene was cloned into HIV^pv^-WT using the In-Fusion system (Takara Bio Inc., Japan) according to the manufacturer’s instructions. Successful cloning was confirmed by Sanger sequencing.

#### HIV-based Env-pseudotype viruses

HIV^pv^-WT and HIV^pv^-MDR Env pseudoviruses were generated by co-transfection of HEK 293-T cells with plasmids encoding the corresponding Env protein and vector at a ratio of 1:3 using 60 μg polyethyleneimine MAX (Polysciences Inc., Warrington, USA). Fresh DMEM containing 10% FCS was added after 8 h of incubation at 37°C. Viral supernatants were harvested 72 h after transfection and the infectivity of the pseudotyped viruses was assessed on TZM-bl cells by measuring the relative light units (RLU) luminescence evoked by the luciferase reporter. If higher titers were needed, virus concentration using sucrose gradient was performed. For this, virus supernatant was sterile filtered and 5 mL sucrose (32% (w/v) in PBS) was added to 25 mL supernatant. Supernatants were centrifuged for 2 h at 28,000 rpm at 4°C and virus pellet was dissolved in 500 μL PBS for 1 h on ice.

#### VSV-based Env-pseudotype viruses

To produce VSV-based Env-pseudotyped viruses (VSV^pv^), 4.5x10^6^ HEK 293-T cells were seeded in Poly-L-Lysine solution (Sigma-Aldrich, St. Louis, USA) treated T-75 flasks. The next day, HEK 293-T cells were transfected with 10 μg of plasmid encoding the corresponding Env protein and 10 μg pCMV-rev (NIH AIDS Reagent Program, Division of AIDS, NIAID, NIH) using Lipofectamine 3000 Transfection Reagent (Thermo Fisher Scientific Inc., Waltham, USA) according to the manufacturer’s instructions. In brief, 750 μL Opti-MEM (Thermo Fisher Scientific Inc., Waltham, USA), plasmid DNA, and 40 μL P3000 Reagent were mixed and added to 28 μL Lipofectamine 3000 Reagent in 750 μL Opti-MEM. After 20 min of incubation, the transfection mix was added to HEK 293-T cells and fresh DMEM containing 5% FCS was added after 4 h of incubation at 37°C. 24 h post transfection, cells were washed with PBS and infected with a recombinant VSV devoid of the G protein and encoding firefly luciferase (VSVΔG∗/rLUC Kerafast, Boston, USA) at a MOI of 3–5.^112^ After 90 min of incubation at 37°C, cells were washed twice with PBS and fresh DMEM containing 5% FCS was added. Viral supernatants were harvested 24 h after infection, centrifuged at 500 x *g* for 10 min and supernatant was stored at −80°C. Infectivity of the VSV^pv^ was assessed on TZM-bl cells by measuring the relative light units (RLU) luminescence evoked by the luciferase reporter.

#### Neutralization assay

The neutralizing capacity of plasma, mAbs, and ARVs efavirenz, emtricitabine, dolutegravir, and darunavir against a panel of 14 viral strains was evaluated on TZM-bl cells using the indicated type of Env pseudotyped viruses in a 384-well format.^86^ In brief, heat-inactivated plasma, mAbs, or ARVs were pre-incubated with the respective virus for 1 h before infection of TZM-bl cells. The input of pseudoviruses was adapted to correspond to 10,000 RLU per well as measured on a Dynex MLX luminescence reader (96-well plates). If infectivity of pseudoviruses was too low to reach this level, undiluted virus was used. Infectivity measured as relative light units (RLU) luminescence evoked by the luciferase reporter was measured on an EnVision Multilabel Reader (PerkinElmer LAS, Germany). Neutralization activity was calculated as the reduction of infectivity compared to infectivity in the absence of plasma, mAbs, or ARVs. Plasma dilutions, mAb or ARV concentrations causing a 50% reduction in infectivity were calculated by fitting a sigmoid dose-response curve (variable slope) to the data using GraphPad Prism version 10 (GraphPad Software, San Diego, USA). If 50% inhibition was not achieved at the highest inhibitor/plasma concentration, a ‘greater than’ value was recorded.

#### Purification of antibodies from plasma

Dynabeads Protein A and Protein G magnetic beads (Thermo Fisher Scientific Inc., Waltham, USA) were resuspended and washed twice with binding buffer (0.1 M sodium phosphate, pH 8.0). In the initial setup, 50 μL of each, Protein A and G beads were incubated with 10 μL of plasma diluted 1:10 in binding buffer at 4°C for 30 min to allow antibody binding. Beads were washed three times with binding buffer and bound antibodies were eluted using 25 μL glycine (0.2 M, pH 2.5). After repetition of the elution step, eluates were neutralized using 10 μL Tris-HCl (1 M, pH 9.0) and the resulting 1:6 dilution of the plasma sample was considered in starting dilutions of downstream experiments. Increased bead concentrations and incubation times were probed as indicated and adjusted in the final protocol to 100 μL of each, Protein A and G beads per 10 μL of plasma diluted 1:10 in binding buffer at 4°C for 24 h. Buffer volumes were adjusted for higher plasma input.

#### ART-DEX: Dissociation and size EXclusion

Samples were purified using Zeba 96-well Spin Desalting Plates, 40K molecular weight cut-off (MWCO) (Thermo Fisher Scientific Inc., Waltham, USA) according to the manufacturer’s instructions. For Figures 4, 5, and 6, plasma samples were purified using Zeba 96-well Spin Desalting Plates (Catalog number: 87774).

Plasma samples were either directly added to the spin plates, following acid treatment alone or after alkaline treatment followed by acid treatment, as indicated. The conditions in the individual treatments and the combined alkaline/acid treatment were identical. The combined alkaline/acid treatment was selected for the ART-DEX protocol. For the alkaline treatment, plasma samples were diluted 1:2.5 in phosphate buffer (0.1 M, pH 10) and incubated for 2 h at room temperature. Zeba 96-well Spin Desalting Plates, 40K MWCO were washed with phosphate buffer (0.1 M, pH 10). Samples were applied to the washed spin plates, centrifuged for 2 min at 1000 x *g*, and the eluted plasma was diluted 1:2 in acetate buffer (0.1 M, pH 3.6). The acidified plasma was then incubated for 1 h at room temperature. Zeba 96-well Spin Desalting Plates, 40K MWCO were washed with acetate buffer (0.1 M, pH 3.6) before a second spin serration of the acidified plasma samples (2 min at 1000 x *g*). The eluted plasma was adjusted to a final dilution of 1:25 in DMEM containing 10% FCS, 25 mM HEPES, and antibiotics.

Zeba 96-well Spin Desalting Plates (Catalog number: 87774) were discontinued close to finalization of our study and replaced by a follow-up product Zeba 96-well Spin Desalting Plates (Catalog number: A57767). To verify that the updated plates perform equally well, we repeated key experiments, namely the MuLV screening data (Figure S4D) and neutralization activity off ART (Figures S5D and S5E) with the updated plates. We adopted the ART-DEX protocol for this slightly according to the manufacturer’s instructions and found it to perform equally well as the protocol with the prior version of the plates. The final ART-DEX protocol based on the Zeba 96-well Spin Desalting Plates (Catalog number: A57767) is as follows:

For the alkaline treatment, plasma samples were diluted 1:2.5 in phosphate buffer (0.1 M, pH 10) and incubated for 2 h at room temperature. Zeba 96-well Spin Desalting Plates, 40K MWCO were washed three times with phosphate buffer (0.1 M, pH 10). Samples were applied to the washed spin plates, centrifuged for 3 min at 700 x *g*, and the eluted plasma was diluted 1:2 in acetate buffer (0.1 M, pH 3.6). The acidified plasma was then incubated for 1 h at room temperature. Zeba 96-well Spin Desalting Plates, 40K MWCO were washed three times with acetate buffer (0.1 M, pH 3.6) before a second spin serration of the acidified plasma samples (3 min at 700 x *g*). The eluted plasma was adjusted to a final dilution of 1:25 in DMEM containing 10% FCS, 25 mM HEPES, and antibiotics.

#### Cell viability assay

Cell viability was assessed using the CellTiter-Glo Luminescent Cell Viability Assay (Promega Corporation, Madison, USA) according to the manufacturer’s instructions. In brief, samples were serially diluted on TZM-bl cells in a 384-well format. After 72 h, CellTiter-Glo reagent was added to the wells and plates were shaken for 2 min. After incubation for 10 min at room temperature, light emission was measured on an EnVision Multilabel Reader (PerkinElmer LAS, Germany).

### Quantification and statistical analysis

#### Statistical analyses

Statistical analyses were performed in R (Version 4.1.0). Linear mixed-effects models were fitted using the lme4 package in R.^113^ Linear models were fitted using the stats package in R. Multiple testing was adjusted using Bonferroni correction for multiple comparisons. Statistical details of each experiment can be found in the corresponding figure legends.

#### Programs

Figures 1, 2, 3, and 4 were generated with GraphPad Prism version 9 (GraphPad Software, San Diego, USA). Figures 5 and 6 were produced using the ggplot2^114^ and ComplexHeatmap^115^ packages in R. Figures 3 and 4 were in part created using BioRender.com (Paid subscription, BioRender, Canada). Figures were assembled and finalized in Affinity Designer (Serif Europe Ltd, United Kingdom).

## Supplemental information

Document S1. Figures S1‒S5 and Tables S1‒S7

Document S2. Article plus supplemental information
