# Supplementary material for: Decoupling HIV-1 antiretroviral drug inhibition from plasma antibody activity to evaluate broadly neutralizing antibody therapeutics and vaccines
Source: Cell Rep Med. 2024 Aug 30;5(9):101702. doi: 10.1016/j.xcrm.2024.101702 (PMC11524982; doi:10.1016/j.xcrm.2024.101702)
Supplement: Document S1. Figures S1‒S5 and Tables S1‒S7 [file mmc1.pdf]

Cell Reports Medicine, Volume *5*

Supplemental information

Decoupling HIV-1 antiretroviral drug inhibition from plasma antibody activity to evaluate broadly neutralizing antibody therapeutics and vaccines

Magdalena Schwarzmüller, Cristina Lozano, Merle Schanz, Irene A. Abela, Silvan Grosse-Holz, Selina Epp, Martina Curcio, Jule Greshake, Peter Rusert, Michael Huber, Roger D. Kouyos, Huldrych F. Günthard, Alexandra Trkola, and the Swiss HIV Cohort Study

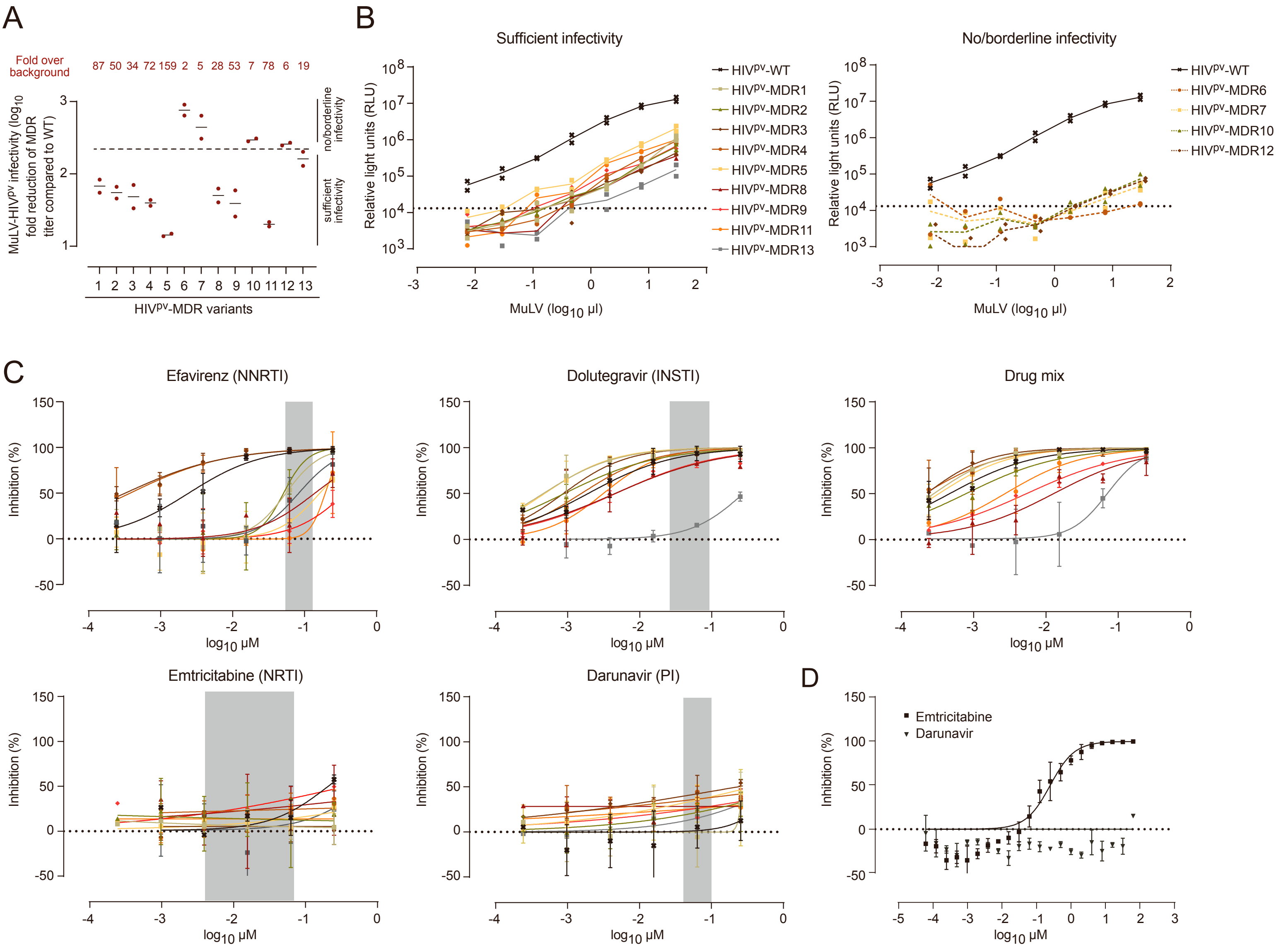

Figure S1. Screening combinations of ART-resistance mutations, related to Figure 1. (A) Infectivity assessment of HIV^{pv}-MDR1-13 pseudotyped with MuLV envelope. Infection of TZM-bl cells with serial dilutions of virus. Infectivity was recorded as relative light units (RLU) luminescence. Fold reduction in infectivity of variants in relation to HIV^{pv}-WT is depicted. Numbers indicate average fold over background values with undiluted virus input. HIV^{pv}-MDR6, 7, 10, and 12 recorded no/borderline infectivity and were not followed further. Data from two independent experiments are shown. (B) Infection of TZM-bl cells with serial dilutions of MuLV-pseudotyped viruses with the indicated resistance mutations. Infectivity was recorded as RLU luminescence and compared to infectivity of HIV^{pv}-WT pseudovirus. Data from two independent experiments are shown. Dotted line indicates mean background signal. Pseudotyped viruses with sufficient infectivity are shown on the left, pseudotyped viruses with no or borderline infectivity are shown on the right. (C) Inhibition of MuLV-pseudotyped viruses with sufficient infectivity by serially diluted antiretroviral drugs was compared to inhibition of HIV^{pv}-WT pseudoviruses on TZM-bl cells. Mean and standard deviation from two independent experiments are shown. Grey boxes indicate drug concentrations expected in plasma of PWH (see Table S4). Drug mix: 25 µM of each, efavirenz (EFV), emtricitabine (FTC), dolutegravir (DTG), and darunavir (DRV) were combined and titrated. (D) Inhibition of MuLV-pseudotyped HIV^{pv}-WT pseudoviruses by serial dilutions of emtricitabine and darunavir. Mean and standard deviation from two independent experiments are shown.

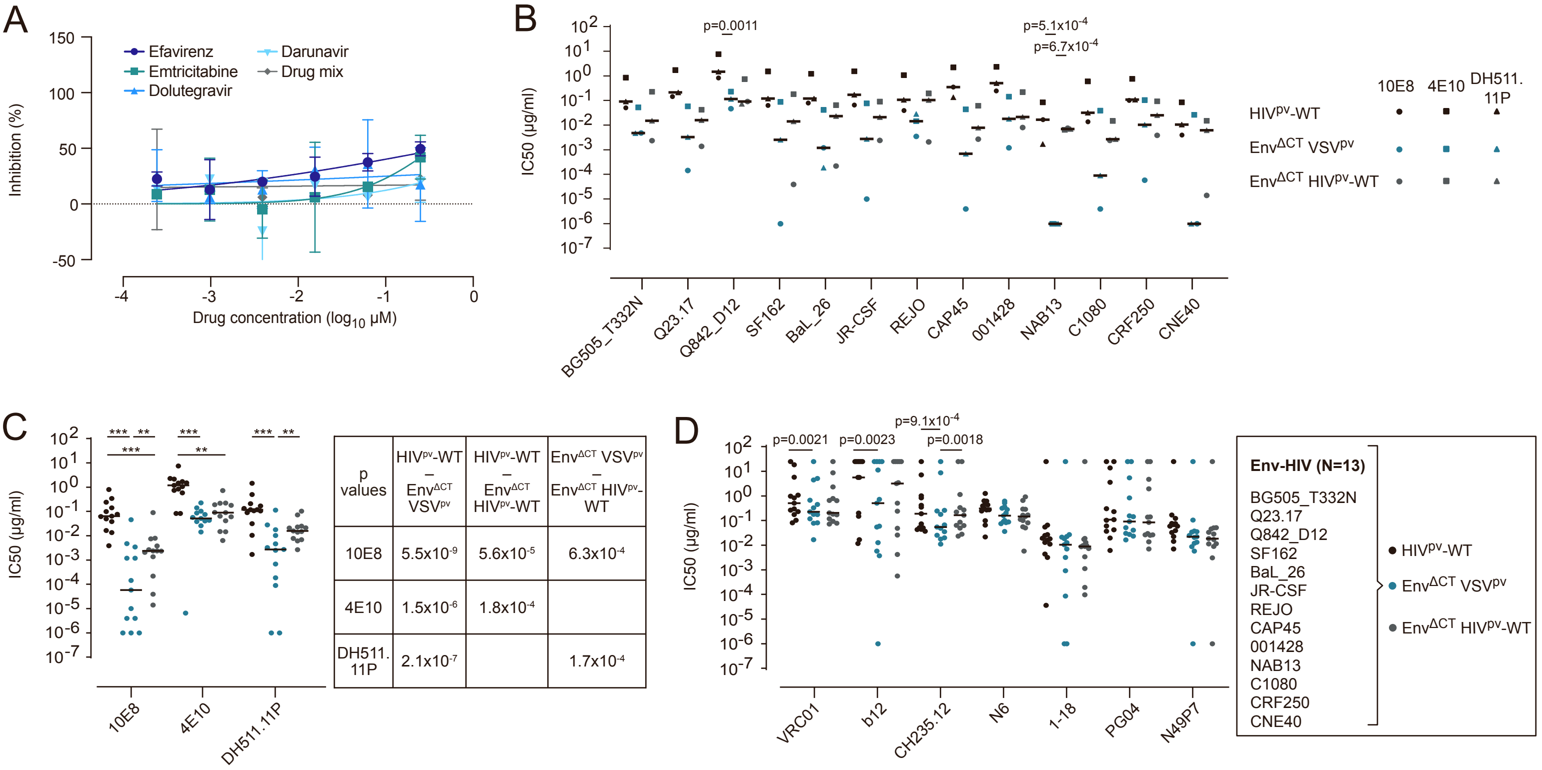

p values	HIV^{pv}-WT – $Env^{\Delta CT}$ VSV^{pv}	HIV^{pv}-WT – $Env^{\Delta CT}$ HIV^{pv}-WT	$Env^{\Delta CT}$ VSV^{pv} – $Env^{\Delta CT}$ HIV^{pv}-WT
10E8	$5.5x10^{-9}$	$5.6x10^{-5}$	$6.3x10^{-4}$
4E10	$1.5x10^{-6}$	$1.8x10^{-4}$	
DH511.11P	$2.1x10^{-7}$		$1.7x10^{-4}$

Figure S2. Complete ARV resistance but partially increased sensitivity to Env-directed mAbs of VSV-based pseudoviruses, related to Figure 2. (A) Inhibition of TZM-bl cell infection of MuLV-pseudotyped VSV^{pv} viruses by serially diluted antiretroviral drugs. Inhibition was assessed as the reduction of infectivity compared to untreated control. Mean and standard deviation of two independent experiments are shown. Drug mix: 25 µM of each, efavirenz, emtricitabine, dolutegravir, and darunavir were combined and titrated. (B)-(C) Comparison of MPER bnAbs for neutralization of 13 Env viruses in the context of HIV^{pv}-WT (black), $Env^{\Delta CT}$ VSV^{pv} (blue), and $Env^{\Delta CT}$ HIV^{pv}-WT (grey) pseudoviruses. Mean IC50 values of two independent experiments are shown. (B) Data depicted per individual Env virus. Significance thresholds between backbones are adjusted on multiple testing using Bonferroni correction and indicated as followed: * p<0.05/39, ** p<0.01/39, *** p<0.001/39. (C) Data are depicted per method across 13 Env virus panel. Significance thresholds between backbones are adjusted on multiple testing using Bonferroni correction and indicated as followed: * p<0.05/9, ** p<0.01/9, *** p<0.001/9. (D) Neutralization of the 13 virus panel by 7 CD4bs-directed mAbs as in (C). Data are depicted per method across 13 Env virus panel. Significance thresholds between backbones are adjusted on multiple testing using Bonferroni correction and indicated as followed: * p<0.05/21, ** p<0.01/21, *** p<0.001/21.

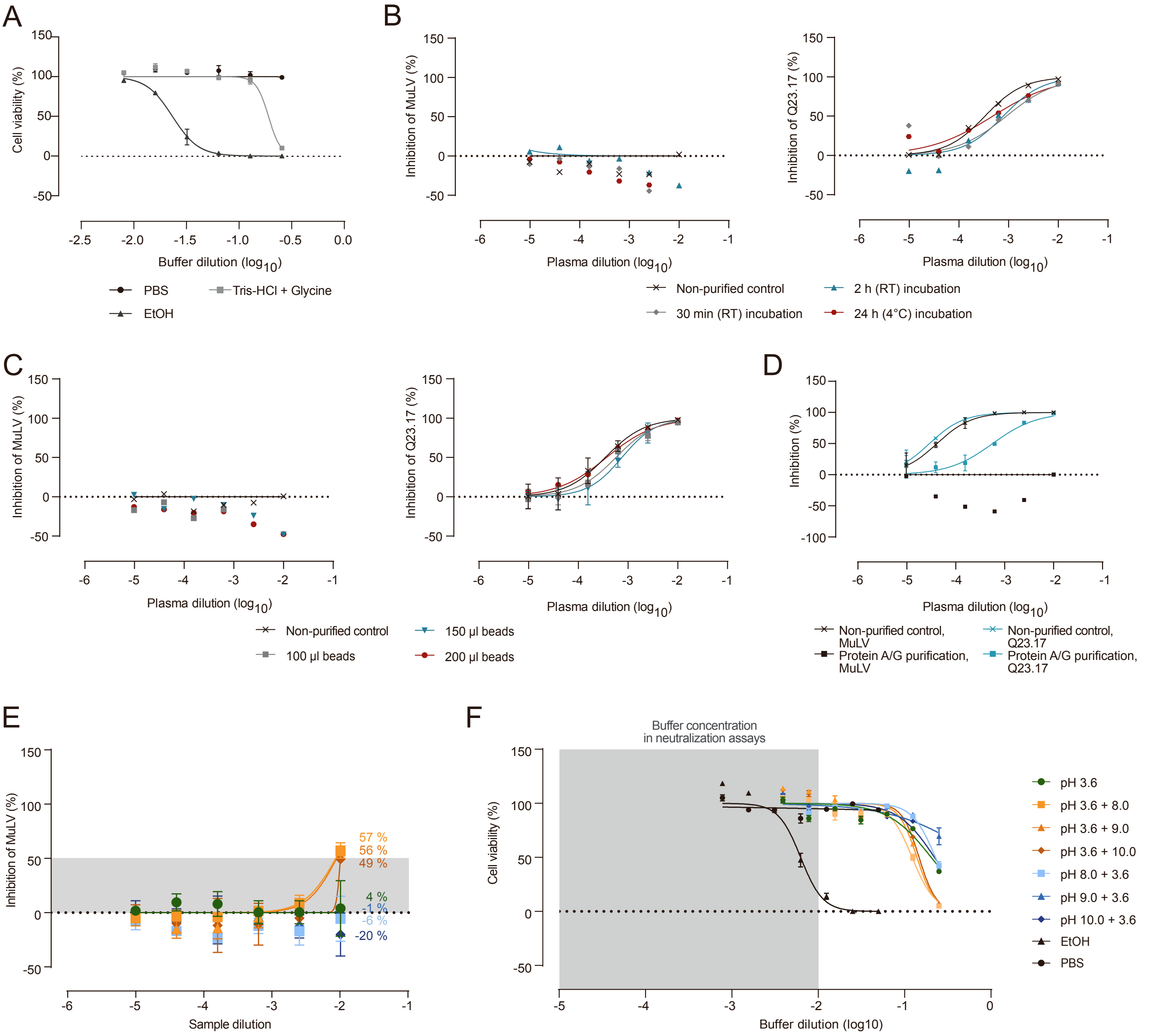

Figure S3. Strategies to separate ARVs from plasma antibodies, related to Figure 3 and 4. (A) Cytotoxicity of the bead elution buffer (100 µl Glycine (0.2 M, pH 2.5) and 20 µl Tris-HCl (1 M, pH 9.0)) compared to treatment with PBS or Ethanol (EtOH) was analyzed on TZM-bl cells. Cell viability was assessed in duplicates and normalized to untreated cells. (B)-(C): Data related to Figure 3. (B) VRC01 (250 µg/ml) spiked into 10 µl healthy donor plasma was added to 100 µl Protein A and G magnetic beads and incubated for the indicated time before antibodies were eluted from the magnetic beads. Inhibition of MuLV (left) and Q23.17 (right) by purified antibody preparations was analyzed in an HIV^{pv}-WT neutralization assay. (C) VRC01 (250 µg/ml) spiked into 10 µl healthy donor plasma was added to indicated amounts of Protein A and G magnetic beads and incubated for 24 h at 4°C before antibodies were eluted from the magnetic beads. Inhibition of MuLV (left) and Q23.17 (right) by purified antibodies was analyzed in an HIV^{pv}-WT neutralization assay. (D) VRC01 (500 µg/ml) and ARVs (25 µM) spiked into 10 µl healthy donor plasma were added to 200 µl Protein A and G magnetic beads and incubated for 24 h at 4°C before antibodies were eluted from the magnetic beads. Inhibition of MuLV and Q23.17 was analyzed in an HIV^{pv}-WT neutralization assay. (E) TZM-bl culture media (without ARVs, mAbs, or plasma) was subjected to the indicated pH and was then used directly in an inhibition assay using MuLV (HIV^{pv}-WT) on TZM-bl cells without prior size-exclusion step. Conditions with acid pH treatment (1 h) are depicted in green, acid pH treatment (2 h) followed by alkaline pH (1 h) in yellow and alkaline pH (2 h) followed by acid pH (1 h) in blue. Percent inhibition at the lowest dilution (= highest concentration) is indicated. Data from two independent experiments are shown. (F) Cytotoxicity of the buffers used for ART-DEX compared to treatment with PBS or Ethanol (EtOH) was analyzed on TZM-bl cells. Cell viability was assessed in duplicates and normalized to untreated cells. Conditions with acid pH treatment (1 h) are depicted in green, acid pH treatment (2 h) followed by alkaline pH (1 h) in yellow and alkaline pH (2 h) followed by acid pH (1 h) in blue. The concentration range of ART-DEX buffers as used in the protocol is indicated with a grey shaded box.

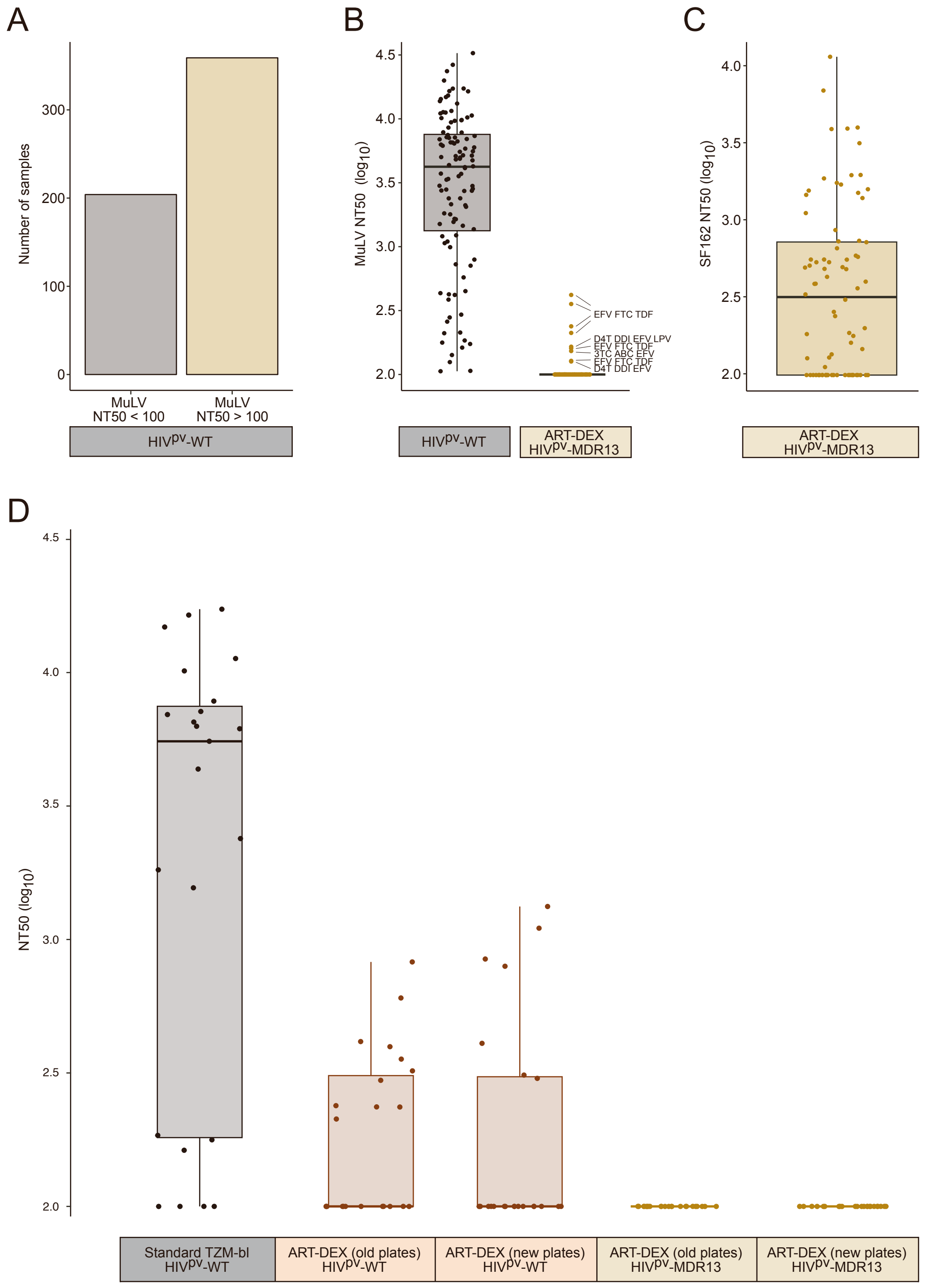

Figure S4. Verifying the capacity of ARV removal strategies in plasma of ART-treated PWH, related to Figure 5. (A) Plasma samples (N=563) from PWH with different ART combination regimen were tested for inhibitory activity against MuLV using the standard TZM-bl assay. Number of samples with (NT50>100) and without (NT50<100) MuLV inhibition are shown. (B) 108 plasma samples with MuLV inhibition in (A) were tested for the inhibitory activity against MuLV either using the standard TZM-bl assay or ART-DEX in combination with HIV^{pv}-MDR13. NT50 titers against MuLV are shown. Drug combinations with residual inhibitory activity are indicated. (C) 80 plasma samples without MuLV inhibition after ART-DEX/HIV^{pv}-MDR13 were tested for their neutralization activity against SF162. NT50 titers against SF162 are shown. (D) Zeba 96-well Spin Desalting Plates (Catalogue number: 87774) were discontinued close to finalization of our study and replaced by a follow-up product Zeba 96-well Spin Desalting Plates (Catalogue number: A57767). To verify that the new plates perform equally well we conducted a control experiment: Plasma samples from PWH (N = 23) with different ART combination regimen were tested for MuLV inhibition using the standard TZM-bl assay (untreated control) or ART-DEX alone or in combination with HIV^{pv}-MDR13. MuLV inhibition after ART-DEX was compared between old and updated spin plates. NT50 titers from two independent experiments are shown. Boxplots represent median with the middle line, upper and lower quartiles with the box limits, and 1.5 x interquartile ranges with the whiskers.

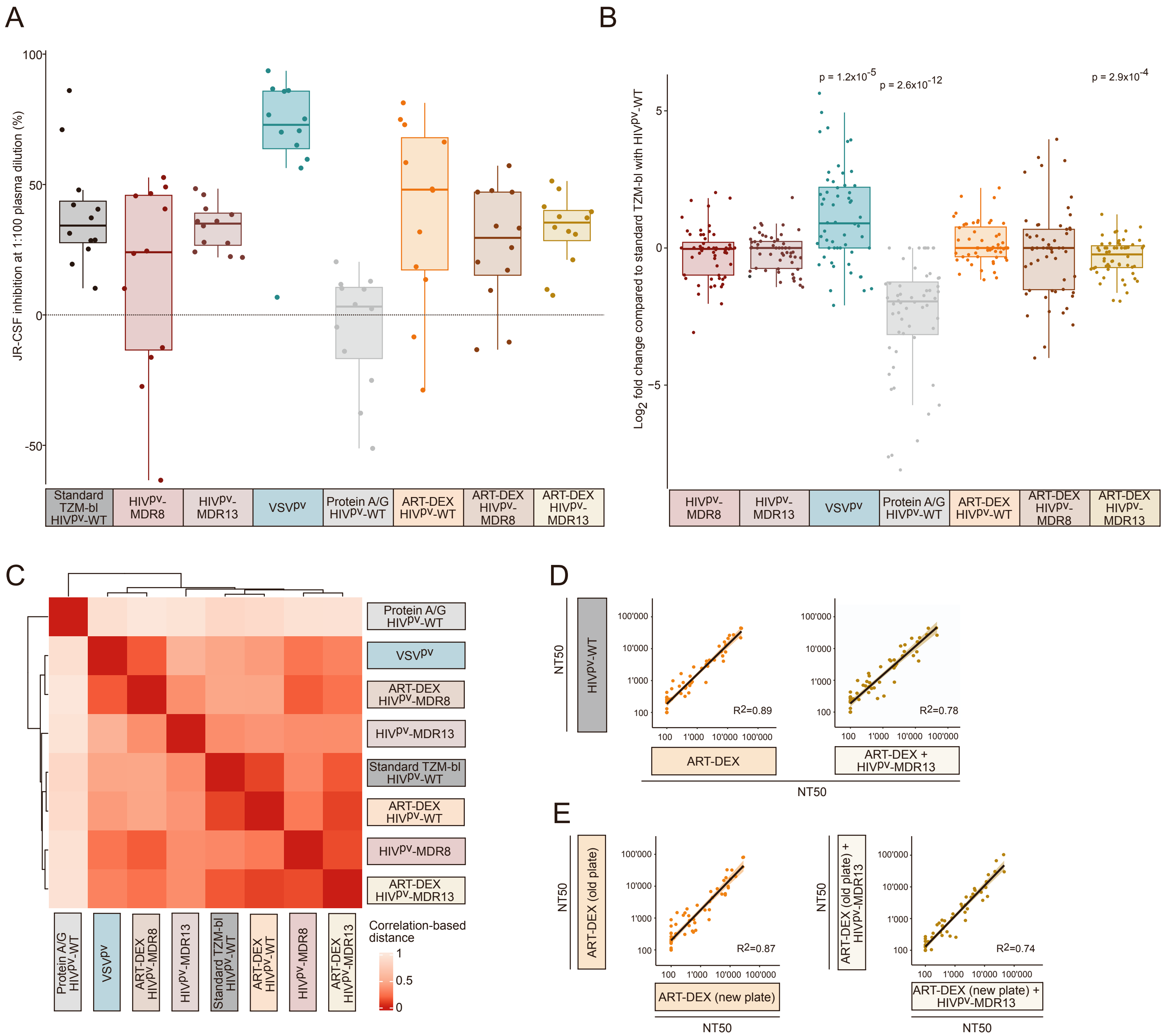

Figure S5. Assessing the accuracy of ART-free neutralization methods in monitoring *in vivo* neutralization capacity, related to Figure 6. Plasma samples of bnAb inducers S5206-G5 and S51517 before the initiation of ART were analyzed for their neutralization activity. (A) Plasma neutralization activity of bnAb inducer S5206-G5 against JR-CSF was compared across all probed ART-free methods. Percent inhibition of JR-CSF at the 1:100 plasma dilution from two independent experiments is shown. Boxplots represent median with the middle line, upper and lower quartiles with the box limits, and 1.5 x interquartile ranges with the whiskers. (B) Fold changes of NT50 titers compared to the standard TZM-bl assay with HIV^{pv}-WT are shown. Significance in variation was tested using one-sided t-test and significance thresholds are adjusted for multiple testing using Bonferroni correction and indicated as followed: * p<0.05/7, ** p<0.01/7, *** p<0.001/7. (C) Heatmap showing the correlation-based distance between the different ART-free neutralization methods and the standard TZM-bl-based neutralization assay. (D) Correlation analysis of NT50 titers based on HIV^{pv}-WT and the ART-DEX (performed with newly released 96-well Spin Desalting Plates (Catalogue number: A57767)) alone or in combination with HIV^{pv}-MDR13. (E) Correlation analysis of NT50 titers based on prior version of 96-well spin plates (Catalogue number: 87774) and updated 96-well spin plates (Catalogue number: A57767) either with ART-DEX alone or in combination with HIV^{pv}-MDR13 confirms equal performance of both plate versions.

Table S1: Virus screening panel, related to Figure 1, 2, 6, and STAR Methods. All viruses were generated as HIV^{pv}-WT, HIV^{pv}-MDR8, HIV^{pv}-MDR13, and VSV^{pv} pseudoviruses with full-length Env or $Env^{\Delta CT}$. Virus titers (RLU/µl) for HIV^{pv}-MDR8 and HIV^{pv}-MDR13 as well as average fold over background with undiluted virus input are listed.

**Virus (full name)**	**Virus (name used in study)**	**Clade**	**Neutralization tier**	**Genebank entry code**	**Infectivity (RLU/µl) with MDR8 (Fold over background)**	**Infectivity (RLU/µl) with MDR13 (Fold over background)**	**Forward primer ΔCT/ΔR**
BG505_W6M_ENV_A5_T332N	BG505_T332N	A	2	DQ208456	11113 (11)	20115 (32)	GTAATACATAGAGTTAGGTAGGGATACTCACCTTTGTCG
Q23.17	Q23.17	A	1B	AF004885	132862 (106)	155205 (153)	CTGTAATAAATAGAGTTAGGTAGGGATACTCACCTTTGTC
Q842_d12_PNS70d	Q842_D12	A	2	AF407160	22289 (18)	32105 (35)	CTGTAATAAATAGAGTTAGGTAGGGATACTCACCTTTGTC
SF162	SF162	B	1A	EU123924	36338 (50)	67276 (54)	GTGAATAGAGTTAGGTAGGGATACTCACCATTATC
BaL_26	BaL_26	B	1B	DQ318211	110905 (109)	92817 (142)	GTGAATAGAGTTAGGTAGGGATACTCACCATTATC
JR-CSF	JR-CSF	B	2	AY669726	54638 (56)	169097 (166)	GTGAATAGAGTTAGGTAGGGATACTCACCCTTATC
REJO4541 clone 67	REJO	B	2	AY835449	73298 (79)	151816 (130)	CTATAATAAATAGAGTTAGGTAGGGATACTCACCATTATCG
CAP45_2_00_G3	CAP45	C	2	DQ435682	15564 (9)	30374 (43)	GTGAAAAGAGTTAGGTAGGGATACTCACCTTTGTC
HIV_001428_2_42	001428	C	2	EF117266	72372 (86)	147854 (170)	GTGAATAGAGTTAGGTAGGGATACTCACCTTTGTC
NAB13pre_cl_9	NAB13	G	2	EU023937	95684 (98)	111330 (127)	CTATAATAAATAGAGTTAGGTAGGGATACTCACCCTTGTC
C1080_c03	C1080	AE	2	JN944660	43610 (55)	68127 (70)	GTAAATAGAGTTAGGTAGGGATACTCACCTTTGTC
CRF02_cl_250	CRF250	AG	2	EU513189	49107 (46)	66399 (95)	GTAATAAATAGAGTTAGGTAGGGATACTCACCTTTGTC
CNE40	CNE40	BC	1B	HM215414	27660 (25)	21322 (34)	GTAAATAGAGTTAGGTAGGGATACTCACCTTTGTC
MuLV	MuLV				12212 (10)	57963 (19)	CAGTGGTCCAGGCTCTATGATTGACTCAACAATATCACC

Table S2: List of monoclonal antibodies used in this study, related to Figure 2.

Monoclonal antibody	Epitope region	Reference
1-18	CD4bs	Schommers 2020 [S1]
B6	CD4bs	Burton 1991 [S2]
b12	CD4bs	Burton 1991 [S2]
CH235.12	CD4bs	Bonsignori 2016 [S3]
N49P7	CD4bs	Sajadi 2018 [S4]
N6	CD4bs	Huang 2016 [S5]
VRC01	CD4bs	Wu 2010 [S6]
PG04	CD4bs	Wu 2011 [S7]
17b	CD4i	Thali 1993 [S8]
48d	CD4i	Thali 1993 [S8]
CH01	V2 glycan	Bonsignori 2011 [S9]
PG16	V2 glycan	Walker 2009 [S10]
PGDM1400	V2 glycan	Sok 2014 [S11]
PGT145	V2 glycan	Walker 2011 [S12]
VRC26.25	V2 glycan	Doria-Rose 2016 [S13]
1-79	V3 glycan	Scheid 2009 [S14]
2G12	V3 glycan	Buchacher 1994 [S15]
447	V3 glycan	Buchbinder 1992 [S16]
PGDM12	V3 glycan	Sok 2016 [S17]
PGDM21	V3 glycan	Sok 2016 [S17]
PGT121	V3 glycan	Walker 2011 [S12]
PGT128	V3 glycan	Walker 2011 [S12]
ACS202	Fusion peptide/ gp41-gp120 interface	Van Gils 2016 [S18]
PGT151	Fusion peptide/ gp41-gp120 interface	Falkowska 2014 [S19]
VRC34.01	Fusion peptide/ gp41-gp120 interface	Kong 2016 [S20]
SF12	Silent face	Schoofs 2019 [S21]
4E10	MPER	Buchacher 1994 [S15]
10E8	MPER	Huang 2012 [S22]
DH511.11P	MPER	Williams 2017 [S23]
Z13e1	MPER	Nelson 2007 [S24]

Table S3: High sensitivity of VSV-based pseudoviruses against certain mAbs, related to Figure 2. Neutralization of a 13 virus panel and MuLV as control by the indicated mAbs was compared between HIV^{pv}-WT, Env$^{\Delta CT}$ VSV^{pv} and Env$^{\Delta CT}$ HIV^{pv}-WT pseudoviruses. Mean IC50 values of two independent experiments are listed.

		IC50 (µg/ml)																													
		bnAbs																									Low/non-neutralizing mAbs					
		CD4bs							V1V2							V3			Fusion peptide/interface			Silent face	MPER			CD4bs	V3		CD4i		MPER
Backbone	Env	VRC01	b12	CH235.12	N6	1-18	PG04	N49P7	PG16	PGDM1400	PGT145	CH01	VRC26.25	PGT121	PGT128	2G12	PGDM12	PGDM21	PGT151	VRC34.01	ACS202	SF12	10E8	4E10	DH511.11P	B6	1-79	447	48d	17b	Z13e1
HIV^{pv}-WT	MuLV	25.0	25.0	25.0	25.0	25.0	25.0	25.0	25.0	25.0	25.0	25.0	25.0	25.0	25.0	25.0	25.0	25.0	25.0	25.0	25.0	25.0	25.0	25.0	25.0	25.0	25.0	25.0	25.0	25.0	25.0
	Bal26	0.17	0.02	0.04	0.25	0.00	0.30	0.03	9.44	6.05	5.66	25.0	25.0	0.03	0.04	0.72	0.04	0.13	0.04	7.99	25.0	0.18	0.08	1.19	0.12	21.5	0.15	0.04	25.0	10.9	25.0
	BG505_T332N	0.27	25.0	0.05	0.39	0.02	0.04	0.08	0.01	0.06	0.06	0.37	0.00	0.03	0.01	0.95	0.03	0.13	0.00	0.14	25.0	0.02	0.05	0.83	0.09	25.0	25.0	25.0	25.0	25.0	16.7
	C1080	5.78	5.65	1.03	1.26	0.07	3.61	0.17	0.00	0.01	0.06	1.76	0.16	25.0	0.24	25.0	19.2	25.0	13.8	3.51	7.82	0.03	0.01	0.59	0.03	25.0	25.0	25.0	20.4	25.0	7.52
	CAP45	18.6	0.20	0.35	0.49	25.0	13.7	0.12	0.00	0.01	0.00	0.06	0.00	5.12	25.0	25.0	25.0	25.0	0.00	0.09	25.0	25.0	0.34	2.15	0.13	25.0	25.0	25.0	2.07	25.0	25.0
	CNE40	1.07	5.64	0.07	0.31	0.03	0.23	0.07	25.0	6.71	0.73	25.0	0.60	0.94	25.0	25.0	23.6	25.0	2.76	0.17	25.0	25.0	0.00	0.08	0.01	0.17	0.33	25.0	25.0	0.08	25.0
	CRF250	25.0	25.0	25.0	0.27	0.02	25.0	25.0	0.00	0.00	0.00	0.11	0.00	0.00	0.01	13.3	0.01	25.0	0.00	0.48	25.0	25.0	0.10	0.75	0.11	25.0	25.0	25.0	8.89	25.0	22.0
	001428	0.08	25.0	0.04	0.07	0.01	0.01	0.01	0.00	1.02	0.15	21.9	3.95	0.05	0.06	25.0	0.11	25.0	0.01	0.16	25.0	0.07	0.24	2.28	0.50	25.0	25.0	25.0	25.0	25.0	25.0
	JRCSF	0.97	0.15	0.19	0.63	0.02	0.11	0.06	0.00	0.03	0.00	25.0	25.0	0.04	0.01	0.62	0.06	0.08	0.02	12.8	0.42	0.04	0.06	1.52	0.17	25.0	25.0	25.0	1.96	25.0	25.0
	NAB13	0.30	25.0	7.24	0.02	0.00	25.0	0.01	7.98	2.67	0.03	25.0	25.0	25.0	25.0	25.0	2.90	25.0	0.02	25.0	25.0	25.0	0.02	0.08	0.00	25.0	2.56	25.0	25.0	25.0	1.30
	Q23.17	0.23	25.0	0.05	0.43	0.02	0.04	0.07	0.00	0.02	1.75	0.01	0.00	0.00	0.03	25.0	0.02	0.02	0.01	0.25	25.0	0.04	0.14	1.70	0.21	25.0	25.0	25.0	25.0	25.0	25.0
	Q842_D12	0.10	25.0	0.04	0.10	0.00	0.02	0.02	0.01	0.01	0.32	9.26	0.21	0.02	0.07	25.0	25.0	25.0	0.00	0.29	25.0	0.03	0.80	7.41	1.45	25.0	25.0	25.0	25.0	25.0	25.0
	REJO	0.51	5.41	0.84	0.28	0.05	0.06	0.04	2.37	0.23	0.00	25.0	25.0	25.0	25.0	25.0	25.0	25.0	0.16	8.31	25.0	0.02	0.04	1.07	0.11	25.0	4.74	25.0	0.88	25.0	23.1
	SF162	0.83	0.01	0.42	0.62	0.01	0.10	0.06	25.0	25.0	25.0	25.0	25.0	0.01	0.02	3.00	0.02	0.01	0.05	0.59	0.14	0.13	0.06	1.49	0.12	2.91	0.00	0.03	3.78	0.40	25.0
Env$^{\Delta CT}$ VSV^{pv}	MuLV	25.0	25.0	25.0	25.0	25.0	25.0	25.0	25.0	25.0	25.0	25.0	25.0	25.0	25.0	25.0	25.0	25.0	25.0	25.0	25.0	25.0	25.0	25.0	25.0	25.0	25.0	25.0	25.0	25.0	25.0
	Bal26	0.08	0.00	0.02	0.10	0.00	0.15	0.02	25.0	23.6	18.2	25.0	25.0	0.00	0.00	0.67	0.08	0.07	0.00	5.13	25.0	0.08	0.00	0.04	0.00	2.70	0.04	0.02	14.3	0.45	2.79
	BG505_T332N	0.18	25.0	0.04	0.32	0.02	0.04	0.04	0.00	0.02	0.01	0.24	0.00	0.02	0.01	0.46	0.13	0.04	0.00	0.06	0.00	0.06	0.00	0.05	0.00	25.0	25.0	25.0	22.3	25.0	2.47
	C1080	5.11	0.72	0.18	0.52	0.02	0.83	0.10	0.00	0.00	0.00	3.80	5.62	25.0	0.06	25.0	25.0	25.0	25.0	3.16	25.0	0.05	0.00	0.04	0.00	25.0	25.0	25.0	20.7	25.0	4.54
	CAP45	4.51	0.06	0.26	0.09	25.0	5.39	0.04	0.00	0.00	0.00	0.02	0.00	2.90	25.0	25.0	25.0	25.0	0.00	0.12	25.0	25.0	0.00	0.04	0.00	25.0	25.0	25.0	3.63	25.0	25.0
	CNE40	0.23	0.01	0.02	0.04	0.00	0.14	0.01	15.2	10.9	0.58	25.0	0.48	0.10	25.0	25.0	25.0	25.0	0.05	0.06	25.0	25.0	0.00	0.03	0.00	0.30	0.18	8.94	25.0	0.02	25.0
	CRF250	25.0	25.0	8.79	0.20	0.01	25.0	25.0	0.00	0.00	0.00	0.12	0.00	0.00	0.00	9.43	0.05	25.0	0.00	0.05	25.0	25.0	0.00	0.10	0.01	25.0	25.0	25.0	5.32	25.0	5.59
	001428	0.08	0.51	0.05	0.07	0.01	0.02	0.01	0.00	3.33	0.48	25.0	9.81	0.00	0.10	25.0	0.30	25.0	0.31	0.10	25.0	0.08	0.00	0.14	0.02	25.0	25.0	25.0	22.0	25.0	25.0
	JRCSF	0.67	0.05	0.07	0.62	0.03	0.02	0.02	0.00	0.06	0.00	5.99	25.0	0.03	0.01	1.02	0.31	0.06	0.02	6.49	0.02	0.06	0.00	0.08	0.00	25.0	25.0	25.0	4.87	25.0	3.07
	NAB13	0.02	25.0	25.0	0.16	0.00	25.0	0.00	25.0	25.0	0.16	25.0	25.0	25.0	25.0	0.95	25.0	25.0	25.0	25.0	25.0	25.0	0.00	0.00	0.00	0.89	0.00	25.0	25.0	25.0	0.01
	Q23.17	0.12	25.0	0.01	0.08	0.01	0.05	0.01	0.00	0.03	0.78	0.00	0.00	0.00	0.02	25.0	0.28	0.01	0.06	0.18	25.0	0.07	0.00	0.06	0.00	25.0	25.0	25.0	25.0	25.09	25.0
	Q842_D12	0.18	11.4	0.03	0.09	0.00	0.03	0.03	0.00	0.00	0.28	1.88	0.00	0.00	0.02	25.0	25.0	25.0	0.02	0.11	25.0	0.06	0.05	0.23	0.11	25.0	25.0	25.0	25.0	25.0	25.0
	REJO	0.33	0.00	0.25	0.28	0.02	0.03	0.01	0.03	0.07	0.00	2.38	25.0	25.0	25.0	25.0	25.0	25.0	0.05	3.01	25.0	0.03	0.00	0.01	0.03	25.0	1.05	25.0	0.49	25.0	25.0
	SF162	0.46	0.01	0.05	0.36	0.00	0.09	0.02	25.0	25.0	13.5	25.0	25.0	0.00	0.01	1.27	0.21	0.01	0.02	0.28	0.08	0.06	0.00	0.09	0.00	0.16	0.01	0.01	0.11	0.17	0.45
Env$^{\Delta CT}$ HIV^{pv}-WT	MuLV	25.0	25.0	25.0	25.0	25.0	25.0	25.0	25.0	25.0	25.0	25.0	25.0	25.0	25.0	25.0	25.0	25.0	25.0	25.0	25.0	25.0	25.0	25.0	25.0	25.0	25.0	25.0	25.0	25.0	25.0
	Bal26	0.08	0.00	0.02	0.11	0.00	0.14	0.01	25.0	3.22	6.32	25.0	25.0	0.01	0.02	0.33	0.08	0.04	0.01	3.47	25.0	0.11	0.00	0.06	0.02	13.5	0.02	0.01	11.8	2.39	2.47
	BG505_T332N	0.18	25.0	0.09	0.29	0.01	0.03	0.05	0.01	0.02	0.03	0.47	0.00	0.02	0.01	0.21	0.09	0.08	0.00	0.07	0.65	0.04	0.00	0.22	0.01	25.0	25.0	25.0	25.0	25.0	5.39
	C1080	2.33	3.47	1.15	0.66	0.02	1.96	0.05	0.00	0.00	0.08	2.49	2.21	25.0	0.10	25.0	25.0	25.0	11.1	0.60	2.73	0.03	0.00	0.01	0.00	25.0	25.0	25.0	15.7	25.0	0.24
	CAP45	8.67	0.03	0.22	0.15	25.0	4.78	0.05	0.00	0.00	0.00	0.03	0.00	2.95	25.0	25.0	25.0	25.0	0.00	0.07	25.0	25.0	0.00	0.06	0.01	25.0	25.0	25.0	2.08	25.0	25.0
	CNE40	0.65	0.24	0.06	0.10	0.01	0.14	0.02	25.0	9.44	0.37	25.0	0.39	0.34	25.0	25.0	25.0	25.0	0.01	0.07	25.0	25.0	0.00	0.01	0.01	0.30	0.21	15.8	25.0	0.07	25.0
	CRF250	25.0	25.0	25.0	0.05	0.02	25.0	25.0	0.00	0.00	0.01	0.10	0.00	0.00	0.01	4.75	0.08	25.0	0.00	0.20	25.0	25.0	0.00	0.09	0.02	25.0	4.93	25.0	23.2	25.0	3.80
	001428	0.07	3.19	0.05	0.06	0.01	0.01	0.00	0.00	4.83	0.35	25.0	25.0	0.06	0.11	25.0	0.46	25.0	0.28	0.22	25.0	0.06	0.01	0.22	0.02	25.0	25.0	25.0	25.0	25.0	25.0
	JRCSF	0.79	0.05	0.16	0.93	0.01	0.09	0.03	0.00	0.02	0.00	25.0	25.0	0.06	0.01	0.46	0.21	0.04	0.02	4.75	0.04	0.03	0.00	0.09	0.02	25.0	25.0	13.0	4.23	25.0	5.26
	NAB13	0.12	25.0	25.0	0.01	0.00	25.0	0.00	25.0	21.5	0.13	25.0	25.0	25.0	25.0	6.09	25.0	25.0	0.24	8.08	13.7	25.0	0.01	0.01	0.01	25.0	0.03	25.0	25.0	25.0	0.02
	Q23.17	0.21	25.0	0.06	0.17	0.01	0.03	0.03	0.00	0.03	1.95	0.00	0.29	0.01	0.03	25.0	0.12	0.01	0.02	0.13	25.0	0.06	0.00	0.04	0.02	25.0	25.0	25.0	25.0	25.0	25.0
	Q842_D12	0.09	25.0	0.03	0.08	0.00	0.01	0.01	0.01	0.00	0.40	10.0	0.15	0.01	0.05	25.0	25.0	25.0	0.02	0.11	25.0	0.02	0.09	0.73	0.07	25.0	25.0	25.0	25.0	25.0	25.0
	REJO	0.17	0.51	0.27	0.17	0.01	0.03	0.01	2.54	0.07	0.00	13.0	25.0	24.7	25.0	25.0	25.0	25.0	0.10	1.95	25.0	0.02	0.00	0.19	0.10	25.0	4.26	25.0	1.11	25.0	9.67
	SF162	0.66	0.00	0.34	0.30	0.00	0.03	0.02	25.0	25.0	25.0	25.0	25.0	0.00	0.01	1.64	0.10	0.01	0.03	0.32	0.01	0.07	0.00	0.18	0.01	1.13	0.00	0.01	0.46	0.22	2.09

Table S4: Plasma concentrations, protein binding and molecular weight of selected antiretroviral drugs, related to Figure 1 and 4. NRTI: Nucleoside reverse transcriptase inhibitor, NNRTI: Non-nucleoside reverse transcriptase inhibitor, PI: Protease inhibitor, INSTI: Integrase strand transfer inhibitor.

Antiretroviral drug	Abbreviation	Drug class	Peak level (mg/l)	Trough level (mg/l)	Protein binding (%)	Molecular weight (g/mol) [S25]
Abacavir	ABC	NRTI	3.1 [S26]	0.006 [S27]	50 [S28]	286.3
Emtricitabine	FTC	NRTI	1.9 [S29]	0.098 [S29]	<5 [S28]	247.3
Lamivudine	3TC	NRTI	1.7 [S26]	0.09 [S30]	<35 [S28]	229.3
Tenofovir	TAF/TDF	NRTI	0.31 [S29]	0.05 [S31]	<10 [S32]	287.2
Zidovudine	AZT	NRTI	1.3 [S26]	0.04 [S33]	34-38 [S28]	267.2
Doravirine	DOR	NNRTI	1.2 [S34]	0.38 [S34]	75 [S35]	425.7
Efavirenz	EFV	NNRTI	4.0 [S28]	1.7 [S28]	>99 [S28]	314.7
Rilpivirine	RPV	NNRTI	0.14 [S36]	0.069 [S37]	>99 [S38]	366.4
Atazanavir	ATV	PI	5.0 [S28]	2.0 [S28]	87 [S28]	704.9
Darunavir	DRV	PI	5.2 [S28]	2.2 [S28]	95 [S28]	547.7
Ritonavir	RTV	PI	11.2 [S28]	3.7 [S28]	89-99 [S28]	720.9
Bictegravir	BIC	INSTI	7.3 [S29]	2.6 [S29]	>99 [S39]	449.4
Dolutegravir	DTG	INSTI	3.9 [S40]	1.1 [S41]	>99 [S42]	419.4
Elvitegravir	EVG	INSTI	1.7 [S40]	0.46 [S43]	98-99 [S44]	447.9

Table S5: Drug combinations of PWH included in the screening for inhibitory activity by ARVs, related to Figure 5. 3TC: Lamivudine, ABC: Abacavir, ATV: Atazanavir, AZT: Zidovudine, BIC: Bictegravir, CAB: Cabotegravir, COB: Cobicistat, D4T: Stavudine, DDI: Didanosine, DOR: Doravirine, DRV: Darunavir, DTG: Dolutegravir, EFV: Efavirenz, ETV: Entecavir, EVG: Elvitegravir, FAPV: Fosamprenavir, FTC: Emtricitabine, IDV: Indinavir, LPV: Lopinavir, MK8591: Islatravir, MRV: Maraviroc, NFV: Nelfinavir, NVP: Nevirapine, RGV: Raltegravir, RPV: Rilpivirine, RTV: Ritonavir, SQV: Saquinavir, TAF/TDF/TXF: Tenofovir.

Screen	Participant ID	Drug combination	Plasma samples (N)	Samples with MuLV inhibition in standard TZM-bl assay (N)	Days from estimated date of infection to first ART initiation	Weeks on treatment	Drug concentration in plasma	
ZPHI screen	Z23	FTC + RPV + TAF	1	1	3824	117		
	Z32	DTG + FTC + RPV + TAF	1	1	28	169		
	Z37	COB + FTC + EVG + TAF	1	1	353	106		
	Z41	BIC +FTC + TAF	1	1	56	132		
	Z44	FTC + RPV + TDF	1	1	23	74		
	Z52	DRV + FTC + RTV + TDF	1	0	25	179	DRV:	4.0 mg/l
	Z96	3TC + AZT + EFV	1	1	56	18	EFV:	8.1 mg/l
	Z97	3TC + ABC + EFV	1	1	44	34	EFV:	1.3 mg/l
	Z98	3TC + ABC + DRV + RTV	1	0	81	56	DRV:	6.5 mg/l
	Z99	EFV + FTC + TDF	1	1	47	197	EFV:	2.0 mg/l
	Z100	DRV + FTC + RTV + TDF	1	0	29	54	DRV:	0.9 mg/l
	Z101	ATV + EFV + FTC + RTV + TDF	1	1	35	131	EFV:	3.0 mg/l
	Z102	DRV + FTC + RTV + TDF	1	0	173	107	DRV:	2.5 mg/l
	Z103	DTG + FTC + TDF	1	1	145	29	DTG:	0.3 mg/l
	Z104	COB + FTC + EVG + TDF	1	1	59	73		
	Z105	DTG + FTC + TAF	1	1	29	56	DTG:	1.8 mg/l
	Z106	COB + FTC + EVG + TAF	1	1	34	105		
	Z107	DTG + FTC + TAF	1	1	24	136	DTG:	3.2 mg/l
	Z108	DTG	1	1	184	85	DTG:	10.6 mg/l
	Z109	BIC + FTC + TAF	1	1	47	94		
	Z110	BIC + DOR + FTC + TAF	1	1	34	73		
	Z263	CAB + RPV	1	1	48	14		
	Z264	CAB + RPV	1	1	103	14		
Extended screen		BIC + FTC + TAF	68	65				
		EFV + FTC + TDF	48	45				
		FTC + RPV + TXF	46	43				
		DRV + FTC + RTV + TDF	38	0				
		ATV + FTC + RTV + TDF	37	2				
		3TC + DTG	27	27				
		DTG + FTC + TXF	24	24				
		3TC + ABC + DTG	18	18				
		COB + FTC + EVG + TXF	17	16				
		3TC + AZT + LPV	14	2				

		FTC + LPV + TDF	14	1				
		FTC + NVP + TXF	13	12				
		FTC + RGV + TXF	16	5				
		DTG + FTC + RPV + TAF	10	10				
		3TC + ABC + ATV + RTV	8	0				
		3TC + ABC + EFV	8	8				
		3TC + ABC + RGV	8	4				
		3TC + AZT + LPV + RTV	8	0				
		3TC + AZT + EFV	7	7				
		3TC + AZT + DRV + RGV + RTV + TDF	6	1				
		BIC + DOR + FTC + TAF	6	6				
		3TC + ABC + DRV + RTV	5	0				
		3TC + AZT + NVP	5	3				
		COB + DRV + FTC + TAF	5	1				
		3TC + ABC + NVP	4	4				
		3TC + NVP	4	4				
		COB + DRV + FTC + EVG + TAF	4	4				
		3TC + ABC + LPV	3	0				
		3TC + AZT + NFV	3	0				
		ATV + COB + FTC + TDF	3	0				
		DRV + FTC + RGV + RTV + TXF	3	0				
		3TC + ABC + D4T	2	0				
		3TC + ABC + EFV + LPV	2	2				
		3TC + ABC + LPV + NVP + TDF	2	1				
		3TC + ABC + LPV + RTV	2	0				
		3TC + ATV + RTV + TDF	2	0				
		3TC + AZT	2	0				
		3TC + AZT + EFV + TDF	2	2				
		3TC + DOR + TDF	2	2				
		3TC + NFV + TDF	2	0				
		ATV + DDI + RTV + TDF	2	0				
		AZT + DRV + RGV + RTV	2	1				
		BIC + DRV + FTC + RTV + TAF	2	2				
		CAB + RPV	2	2				
		COB + DRV + DTG	2	2				
		D4T + DDI + EFV + LPV	2	2				
		D4T + DDI + NFV	2	0				
		DOR + DRV + RGV + RTV	2	2				
		DOR + DTG	2	2				
		DRV + ETV + RGV + RTV	2	2				
		DTG + FTC	2	2				
		DTG + FTC + ETV + TAF	2	2				

		DTG + RPV	2	2				
		EFV + LPV + RTV + TDF	2	2				
		ETV + RGV + TDF	2	2				
		FAPV + MRV + RGV + RTV	2	1				
		FTC + FAPV + RTV + TDF	2	0				
		FTC + LPV + RTV + TDF	2	0				
		FTC + MRV + RGV + TAF	2	0				
		FTC + NFV + TDF	2	0				
		3TC + ABC + ATV + RTV + TDF	1	0				
		3TC + ABC + AZT	1	0				
		3TC + ABC + DOR + DTG	1	1				
		3TC + ABC + DRV + RGV + RTV	1	1				
		3TC + AZT + FAPV + RTV + TDF	1	0				
		3TC + DDI + EFV	1	1				
		3TC + DDI + NVP	1	1				
		3TC + DOR + DTG	1	1				
		3TC + LPV + TDF	1	0				
		ABC + ATV + FTC + RTV + TDF	1	0				
		ABC + DDI + EFV	1	1				
		ATV + DDI + FTC + RTV + TDF	1	0				
		ATV + EFV + FTC + RTV + TDF	1	1				
		AZT	1	0				
		AZT + DDI	1	0				
		AZT + FAPV + LPV + TDF	1	0				
		AZT + IDV + NVP + TDF	1	1				
		COB + DRV + DTG + FTC + TAF	1	1				
		D4T + DDI + EFV	1	1				
		D4T + RTV + SQV	1	0				
		DRV + RGV + RTV	1	1				
		DTG	1	1				
		EFV + FAPV + RTV + TDF	1	1				
		FTC + TDF	1	0				
		MK8591	1	1				

Table S6: Neutralization titers (NT50) of longitudinal plasma samples from known bnAb inducers, related to Figure 6.

		Year	MuLV	BG505_T332N	Q23.17	Q842_D12	SF162	Bal26	JRCSF	REJO	CAP45	001428	NAB13	C1080	CRF250	CNE40	Potency (Geometric mean NT50)	Breadth (%)
S5206-G5	HIV^{pv}-WT	2007	100	1374	26238	5973	919	227	100	213	8060	248	293	16305	7893	4050	1944	92
		2009	100	2437	44446	13091	874	265	100	131	21911	870	320	43615	27296	21043	3699	92
		2010	100	859	46941	7559	731	159	100	100	13107	479	418	20554	8682	7452	2903	85
		2011	100	1867	38827	8671	1311	148	100	100	24004	412	284	22113	13672	6121	3368	85
		2012	100	653	20377	3108	436	100	100	100	10321	407	124	20962	7806	6100	2495	77
		2013	294	877	12815	5243	1315	364	322	470	7095	732	380	11188	7221	3866	1833	100
	HIV^{pv}-MDR8	2007	100	1210	33141	5234	859	921	100	211	7207	875	148	18269	6511	7114	2345	92
		2009	100	941	128320	1547	854	203	100	103	8731	1321	312	47060	6659	32367	2655	92
		2010	100	273	171406	2254	470	200	100	100	24817	695	148	18729	6032	27919	2791	85
		2011	100	530	192222	6998	1059	581	100	131	10284	1212	190	25984	10249	4107	2698	92
		2012	100	280	84621	5097	399	194	100	100	37759	539	100	24194	5846	3436	3138	77
		2013	100	100	37826	547	288	101	100	100	1819	1167	100	7466	4242	3198	1718	69
	HIV^{pv}-MDR13	2007	100	636	93764	8369	931	267	100	100	6412	445	150	19331	6770	3452	2502	85
		2009	100	1459	79085	6375	1076	236	100	143	27343	1449	181	34072	10644	12291	3120	92
		2010	100	751	70463	4748	392	137	100	100	7377	1235	185	14289	11447	6083	2487	85
		2011	131	810	69096	5446	244	223	100	132	8856	1053	100	8843	5977	7024	2253	85
		2012	100	816	126008	8045	888	129	100	100	12973	945	117	16559	8469	4949	2832	85
		2013	100	1275	46013	2149	208	106	100	100	5309	1115	102	8181	5670	2345	1605	85
	VSV^{pv}	2007	100	659	17994	7939	4438	1507	351	632	9026	162	8996	87160	13753	5629	3514	100
		2009	100	1175	152845	8211	13414	5000	4990	535	23773	691	6659	28826	31972	7516	7402	100
		2010	100	2008	42290	5944	1724	1199	332	241	10799	726	19805	10026	10872	6501	3433	100
		2011	100	4043	21547	6106	3891	3158	216	201	15493	123	4396	32334	17084	5423	3366	100
		2012	100	1047	37618	10943	3627	1471	125	242	16695	159	6670	10823	26281	24410	3414	100
		2013	100	839	6389	3598	1035	213	100	100	4880	136	1869	7872	33053	1185	1959	85
	Protein A/G	2007	100	100	394	113	262	106	100	100	251	100	100	1311	229	1067	316	62
		2009	100	100	226	100	135	100	100	100	116	100	131	1225	100	1029	289	46
		2010	100	100	264	100	245	100	100	100	113	100	100	570	100	1247	350	38
		2011	100	100	317	100	286	100	100	100	176	100	100	749	100	1338	437	38
		2012	100	100	168	100	156	100	100	100	134	100	100	1057	154	1412	305	46
		2013	100	100	255	100	104	100	100	100	186	100	100	604	142	671	257	46
	ART-DEX	2007	100	899	32051	5831	412	160	167	114	15219	886	560	32642	9870	18490	2023	100
		2009	100	1787	79733	8857	889	260	100	184	40084	1937	1180	81359	20150	16817	4761	92
		2010	100	711	47072	4634	674	150	100	126	13558	997	439	18538	24248	8900	2451	92
		2011	100	523	35894	6560	372	208	100	100	13564	822	304	45063	21820	12295	3223	85
		2012	100	1107	55318	4317	595	148	333	100	26650	531	162	34589	13380	12375	2614	92
		2013	100	402	25857	2213	226	100	100	100	10990	1293	100	13952	10071	7767	3509	69
	ART-DEX + HIV^{pv}-MDR8	2007	100	247	210986	1156	672	398	100	306	2311	2444	100	148197	15697	11204	3410	85
		2009	100	151	693244	1071	797	477	100	210	79321	2014	100	39330	185584	10249	5370	85
		2010	100	166	389749	541	533	285	100	100	132605	592	141	22135	5593	2301	2422	85
		2011	100	388	124297	24679	938	313	100	135	6147	1084	130	17748	8202	6075	2397	92
		2012	100	744	164823	1453	453	110	100	100	19628	472	100	18510	7379	918	2521	77
		2013	100	100	34228	231	117	117	100	100	3578	1526	100	59078	1668	5869	1913	69
	ART-DEX + HIV^{pv}-MDR13	2007	100	592	29927	3763	371	230	100	100	8870	345	193	14643	9585	4993	2025	85
		2009	100	1586	103933	7837	873	191	100	100	24242	1047	257	67017	30654	6768	4600	85
		2010	100	689	62486	3973	597	112	100	100	9854	1122	242	12227	12774	3474	2418	85
		2011	100	547	38224	3109	615	100	100	100	9297	755	100	17283	11550	3146	3827	69
		2012	100	265	35435	3476	236	100	100	100	10160	484	100	5399	8388	1548	2402	69
		2013	100	396	38538	1920	257	100	100	100	7741	627	100	7970	3806	1779	2323	69
S51517	HIV^{pv}-WT	2008	100	666	427	4085	11986	1655	2671	3235	302	4377	984	555	294	4403	1495	100
		2010	100	261	203	699	5898	843	411	577	100	624	393	266	100	2818	645	85
		2011	100	182	132	299	3872	307	295	456	100	408	293	114	100	3048	405	85
		2012	100	180	164	646	7947	349	357	601	101	308	284	185	100	2274	432	92
		2013	142	399	215	529	4297	393	463	495	274	355	344	384	243	1921	494	100
		2014	125	222	173	369	3032	461	449	444	232	370	226	247	249	1739	406	100
	HIV^{pv}-MDR8	2008	100	302	1418	2598	5556	1663	2393	4543	290	6857	931	207	141	2913	1246	100
		2010	100	100	286	318	1834	1833	191	810	100	873	197	100	100	2928	660	69
		2011	100	451	122	429	2753	519	130	411	100	854	175	100	100	1320	452	77
		2012	100	100	123	482	2700	676	289	464	100	432	241	152	100	1822	459	77
		2013	100	100	100	110	2435	809	100	173	100	153	100	100	100	643	393	46
		2014	100	100	100	100	993	232	100	100	100	203	100	100	100	1178	484	31
	HIV^{pv}-MDR13	2008	100	743	572	1860	7053	1455	1448	2822	244	3036	366	718	164	1846	1053	100
		2010	100	306	300	583	4330	758	763	798	100	991	170	283	100	3267	689	85
		2011	100	109	119	398	2488	424	308	645	100	570	196	131	100	2083	391	85
		2012	100	401	125	256	1482	1028	227	369	150	270	138	237	100	1971	357	92
		2013	218	290	204	153	2485	401	276	480	236	280	209	314	256	2189	375	100
		2014	100	155	128	240	1029	296	227	252	100	184	245	121	118	1256	252	92
	VSV^{pv}	2008	100	3240	100	5462	22905	11457	2403	5948	960	1487	7849	2060	100	2342	3943	85
		2010	100	568	1134	2828	19403	2550	777	883	1477	623	2662	1221	387	3437	1527	100
		2011	100	196	104	618	12391	1340	831	353	100	131	2252	158	100	1372	604	85
		2012	100	295	100	4273	4699	3505	227	548	100	171	733	763	100	4757	1016	77

		2013	100	100	100	328	5963	1219	100	100	210	209	2005	142	100	858	632	62
		2014	100	100	100	325	12553	367	100	142	100	100	2331	130	100	2368	764	54
	Protein A/G	2008	100	189	100	690	1982	217	318	973	100	1224	136	334	100	458	462	77
		2010	100	100	100	132	1481	164	100	125	100	194	100	139	100	628	254	54
		2011	100	100	100	100	1649	111	100	111	100	121	100	140	100	372	225	46
		2012	100	100	100	100	1290	183	100	100	100	105	100	215	100	555	312	38
		2013	100	100	100	100	937	106	100	100	100	100	100	100	100	314	315	23
		2014	100	100	100	100	889	100	100	100	100	100	100	100	100	127	336	15
	ART-DEX	2008	100	1222	337	3274	5635	1450	2521	1822	328	4385	1481	633	217	8322	1448	100
		2010	100	239	179	360	5311	711	391	578	200	842	307	322	100	6789	581	92
		2011	100	230	100	317	5189	520	401	459	104	525	455	188	100	3023	502	85
		2012	100	172	100	452	4161	503	182	330	124	366	382	344	100	2737	452	85
		2013	100	159	100	251	1698	267	100	200	100	224	144	247	100	4535	370	69
		2014	100	103	100	168	1493	168	103	248	100	217	113	305	100	1307	253	77
	ART-DEX + HIV^{pv}-MDR8	2008	100	802	684	3133	3545	1604	578	4385	100	2939	345	918	100	883	1315	85
		2010	100	163	282	100	4725	1040	541	1031	100	604	176	289	100	911	568	77
		2011	100	100	224	158	4009	684	163	712	100	351	149	100	100	910	433	69
		2012	100	159	122	182	1577	510	229	401	100	289	170	489	100	852	332	85
		2013	100	134	100	100	2207	418	100	363	100	150	100	100	100	453	381	46
		2014	100	100	100	100	1089	257	100	145	100	100	100	100	100	1043	453	31
	ART-DEX + HIV^{pv}-MDR13	2008	100	724	270	2395	4719	893	1403	2032	134	2682	256	849	135	2843	872	100
		2010	100	187	125	637	5923	491	293	384	100	588	104	279	150	4793	445	92
		2011	100	122	104	270	3411	239	225	342	100	417	236	100	100	3721	389	77
		2012	100	421	100	224	2911	335	174	284	252	220	229	193	100	864	350	85
		2013	100	127	100	278	1985	230	141	183	100	182	107	199	123	666	237	85
		2014	100	106	100	217	874	234	225	143	105	122	154	173	180	513	203	92

Table S7: Summary of tested ART-free neutralization methods, related to Figure 5, 6, and Discussion.

ART-free neutralization method	Advantages	Disadvantages
HIV^{pv}-MDR8	No additional sample preparation, use with standard TZM-bl assay	Low infectivity, residual inhibitory activity (especially for second generation INSTIs)
HIV^{pv}-MDR13	No additional sample preparation, use with standard TZM-bl assay	Low infectivity, residual inhibitory activity (especially for EFV-containing regimen)
VSV^{pv}	Complete resistance against all groups of HIV enzyme-targeting ARVs	Increase in sensitivity compared to standard TZM-bl assay using HIV^{pv}-WT, possibly differences in entry process as based on VSV backbone
Protein A/G + HIV^{pv}-WT	Compatibility with all groups of ARVs except antibody-based entry inhibitors	Additional sample preparation needed, loss of antibodies, need for normalized antibody input
ART-DEX + HIV^{pv}-WT	Use with HIV^{pv}-WT, good correlation with titers obtained with standard TZM-bl assay	Incomplete removal of ARVs (especially EFV and second-generation INSTIs), sample preparation needed
ART-DEX + HIV^{pv}-MDR8	Removal of inhibitory activity of ARVs (especially EFV)	Low infectivity, sample preparation needed
ART-DEX + HIV^{pv}-MDR13	Complete removal of inhibitory activity of ARVs (especially second-generation INSTIs), good correlation with titers obtained with standard TZM-bl assay	Low infectivity, sample preparation needed

References

1. Schommers, P., Gruell, H., Abernathy, M.E., Tran, M.K., Dingens, A.S., Gristick, H.B., Barnes, C.O., Schoofs, T., Schlotz, M., Vanshylla, K., Kreer, C., Weiland, D., Holtick, U., Scheid, C., Valter, M.M., van Gils, M.J., Sanders, R.W., Vehreschild, J.J., Cornely, O.A., Lehmann, C., Fatkenheuer, G., Seaman, M.S., Bloom, J.D., Bjorkman, P.J., and Klein, F. (2020). Restriction of HIV-1 Escape by a Highly Broad and Potent Neutralizing Antibody. Cell *180*, 471-489 e422. 10.1016/j.cell.2020.01.010.
2. Burton, D.R., Barbas, C.F., 3rd, Persson, M.A., Koenig, S., Chanock, R.M., and Lerner, R.A. (1991). A large array of human monoclonal antibodies to type 1 human immunodeficiency virus from combinatorial libraries of asymptomatic seropositive individuals. Proc Natl Acad Sci U S A *88*, 10134-10137. 10.1073/pnas.88.22.10134.
3. Bonsignori, M., Zhou, T., Sheng, Z., Chen, L., Gao, F., Joyce, M.G., Ozorowski, G., Chuang, G.Y., Schramm, C.A., Wiehe, K., Alam, S.M., Bradley, T., Gladden, M.A., Hwang, K.K., Iyengar, S., Kumar, A., Lu, X., Luo, K., Mangiapani, M.C., Parks, R.J., Song, H., Acharya, P., Bailer, R.T., Cao, A., Druz, A., Georgiev, I.S., Kwon, Y.D., Louder, M.K., Zhang, B., Zheng, A., Hill, B.J., Kong, R., Soto, C., Program, N.C.S., Mullikin, J.C., Douek, D.C., Montefiori, D.C., Moody, M.A., Shaw, G.M., Hahn, B.H., Kelsoe, G., Hraber, P.T., Korber, B.T., Boyd, S.D., Fire, A.Z., Kepler, T.B., Shapiro, L., Ward, A.B., Mascola, J.R., Liao, H.X., Kwong, P.D., and Haynes, B.F. (2016). Maturation Pathway from Germline to Broad HIV-1 Neutralizer of a CD4-Mimic Antibody. Cell *165*, 449-463. 10.1016/j.cell.2016.02.022.
4. Sajadi, M.M., Dashti, A., Rikhtegaran Tehrani, Z., Tolbert, W.D., Seaman, M.S., Ouyang, X., Gohain, N., Pazgier, M., Kim, D., Cavet, G., Yared, J., Redfield, R.R., Lewis, G.K., and DeVico, A.L. (2018). Identification of Near-Pan-neutralizing Antibodies against HIV-1 by Deconvolution of Plasma Humoral Responses. Cell *173*, 1783-1795 e1714. 10.1016/j.cell.2018.03.061.
5. Huang, J., Kang, B.H., Ishida, E., Zhou, T., Griesman, T., Sheng, Z., Wu, F., Doria-Rose, N.A., Zhang, B., McKee, K., O'Dell, S., Chuang, G.Y., Druz, A., Georgiev, I.S., Schramm, C.A., Zheng, A., Joyce, M.G., Asokan, M., Ransier, A., Darko, S., Migueles, S.A., Bailer, R.T., Louder, M.K., Alam, S.M., Parks, R., Kelsoe, G., Von Holle, T., Haynes, B.F., Douek, D.C., Hirsch, V., Seaman, M.S., Shapiro, L., Mascola, J.R., Kwong, P.D., and Connors, M. (2016). Identification of a CD4-Binding-Site Antibody to HIV that Evolved Near-Pan Neutralization Breadth. Immunity *45*, 1108-1121. 10.1016/j.immuni.2016.10.027.
6. Wu, X., Yang, Z.Y., Li, Y., Hogerkorp, C.M., Schief, W.R., Seaman, M.S., Zhou, T., Schmidt, S.D., Wu, L., Xu, L., Longo, N.S., McKee, K., O'Dell, S., Louder, M.K., Wycuff, D.L., Feng, Y., Nason, M., Doria-Rose, N., Connors, M., Kwong, P.D., Roederer, M., Wyatt, R.T., Nabel, G.J., and Mascola, J.R. (2010). Rational design of envelope identifies broadly neutralizing human monoclonal antibodies to HIV-1. Science *329*, 856-861. 10.1126/science.1187659.
7. Wu, X., Zhou, T., Zhu, J., Zhang, B., Georgiev, I., Wang, C., Chen, X., Longo, N.S., Louder, M., McKee, K., O'Dell, S., Perfetto, S., Schmidt, S.D., Shi, W., Wu, L., Yang, Y., Yang, Z.Y., Yang, Z., Zhang, Z., Bonsignori, M., Crump, J.A., Kapiga, S.H., Sam, N.E., Haynes, B.F., Simek, M., Burton, D.R., Koff, W.C., Doria-Rose, N.A., Connors, M., Program, N.C.S., Mullikin, J.C., Nabel, G.J., Roederer, M., Shapiro, L., Kwong, P.D., and Mascola, J.R. (2011). Focused evolution of HIV-1 neutralizing antibodies revealed by structures and deep sequencing. Science *333*, 1593-1602. 10.1126/science.1207532.
8. Thali, M., Moore, J.P., Furman, C., Charles, M., Ho, D.D., Robinson, J., and Sodroski, J. (1993). Characterization of conserved human immunodeficiency virus type 1 gp120 neutralization epitopes exposed upon gp120-CD4 binding. J Virol *67*, 3978-3988. 10.1128/JVI.67.7.3978-3988.1993.
9. Bonsignori, M., Hwang, K.K., Chen, X., Tsao, C.Y., Morris, L., Gray, E., Marshall, D.J., Crump, J.A., Kapiga, S.H., Sam, N.E., Sinangil, F., Pancera, M., Yongping, Y., Zhang, B., Zhu, J., Kwong, P.D., O'Dell, S., Mascola, J.R., Wu, L., Nabel, G.J., Phogat, S., Seaman, M.S., Whitesides, J.F., Moody, M.A., Kelsoe, G., Yang, X., Sodroski, J., Shaw, G.M., Montefiori, D.C., Kepler, T.B., Tomaras, G.D., Alam, S.M., Liao, H.X., and Haynes, B.F. (2011). Analysis of a clonal lineage of HIV-1 envelope V2/V3 conformational epitope-specific broadly neutralizing antibodies and their inferred unmutated common ancestors. J Virol *85*, 9998-10009. 10.1128/JVI.05045-11.

10. Walker, L.M., Phogat, S.K., Chan-Hui, P.Y., Wagner, D., Phung, P., Goss, J.L., Wrin, T., Simek, M.D., Fling, S., Mitcham, J.L., Lehrman, J.K., Priddy, F.H., Olsen, O.A., Frey, S.M., Hammond, P.W., Protocol, G.P.I., Kaminsky, S., Zamb, T., Moyle, M., Koff, W.C., Poignard, P., and Burton, D.R. (2009). Broad and potent neutralizing antibodies from an African donor reveal a new HIV-1 vaccine target. Science *326*, 285-289. 10.1126/science.1178746.
11. Sok, D., van Gils, M.J., Pauthner, M., Julien, J.P., Saye-Francisco, K.L., Hsueh, J., Briney, B., Lee, J.H., Le, K.M., Lee, P.S., Hua, Y., Seaman, M.S., Moore, J.P., Ward, A.B., Wilson, I.A., Sanders, R.W., and Burton, D.R. (2014). Recombinant HIV envelope trimer selects for quaternary-dependent antibodies targeting the trimer apex. Proc Natl Acad Sci U S A *111*, 17624-17629. 10.1073/pnas.1415789111.
12. Walker, L.M., Huber, M., Doores, K.J., Falkowska, E., Pejchal, R., Julien, J.P., Wang, S.K., Ramos, A., Chan-Hui, P.Y., Moyle, M., Mitcham, J.L., Hammond, P.W., Olsen, O.A., Phung, P., Fling, S., Wong, C.H., Phogat, S., Wrin, T., Simek, M.D., Protocol, G.P.I., Koff, W.C., Wilson, I.A., Burton, D.R., and Poignard, P. (2011). Broad neutralization coverage of HIV by multiple highly potent antibodies. Nature *477*, 466-470. 10.1038/nature10373.
13. Doria-Rose, N.A., Bhiman, J.N., Roark, R.S., Schramm, C.A., Gorman, J., Chuang, G.Y., Pancera, M., Cale, E.M., Ernandes, M.J., Louder, M.K., Asokan, M., Bailer, R.T., Druz, A., Fraschilla, I.R., Garrett, N.J., Jarosinski, M., Lynch, R.M., McKee, K., O'Dell, S., Pegu, A., Schmidt, S.D., Staupe, R.P., Sutton, M.S., Wang, K., Wibmer, C.K., Haynes, B.F., Abdool-Karim, S., Shapiro, L., Kwong, P.D., Moore, P.L., Morris, L., and Mascola, J.R. (2016). New Member of the V1V2-Directed CAP256-VRC26 Lineage That Shows Increased Breadth and Exceptional Potency. J Virol *90*, 76-91. 10.1128/JVI.01791-15.
14. Scheid, J.F., Mouquet, H., Feldhahn, N., Seaman, M.S., Velinzon, K., Pietzsch, J., Ott, R.G., Anthony, R.M., Zebroski, H., Hurley, A., Phogat, A., Chakrabarti, B., Li, Y., Connors, M., Pereyra, F., Walker, B.D., Wardemann, H., Ho, D., Wyatt, R.T., Mascola, J.R., Ravetch, J.V., and Nussenzweig, M.C. (2009). Broad diversity of neutralizing antibodies isolated from memory B cells in HIV-infected individuals. Nature *458*, 636-640. 10.1038/nature07930.
15. Buchacher, A., Predl, R., Strutzenberger, K., Steinfellner, W., Trkola, A., Purtscher, M., Gruber, G., Tauer, C., Steindl, F., Jungbauer, A., and et al. (1994). Generation of human monoclonal antibodies against HIV-1 proteins; electrofusion and Epstein-Barr virus transformation for peripheral blood lymphocyte immortalization. AIDS Res Hum Retroviruses *10*, 359-369. 10.1089/aid.1994.10.359.
16. Buchbinder, A., Zolla-Pazner, S., Karwowska, S., Gorny, M.K., and Burda, S.T. (1992). Synergy between human monoclonal antibodies to HIV extends their effective biologic activity against homologous and divergent strains. AIDS Res Hum Retroviruses *8*, 1395. 10.1089/aid.1992.8.1395.
17. Sok, D., Pauthner, M., Briney, B., Lee, J.H., Saye-Francisco, K.L., Hsueh, J., Ramos, A., Le, K.M., Jones, M., Jardine, J.G., Bastidas, R., Sarkar, A., Liang, C.H., Shivatare, S.S., Wu, C.Y., Schief, W.R., Wong, C.H., Wilson, I.A., Ward, A.B., Zhu, J., Poignard, P., and Burton, D.R. (2016). A Prominent Site of Antibody Vulnerability on HIV Envelope Incorporates a Motif Associated with CCR5 Binding and Its Camouflaging Glycans. Immunity *45*, 31-45. 10.1016/j.immuni.2016.06.026.
18. van Gils, M.J., van den Kerkhof, T.L., Ozorowski, G., Cottrell, C.A., Sok, D., Pauthner, M., Pallesen, J., de Val, N., Yasmeen, A., de Taeye, S.W., Schorcht, A., Gumbs, S., Johanna, I., Saye-Francisco, K., Liang, C.H., Landais, E., Nie, X., Pritchard, L.K., Crispin, M., Kelsoe, G., Wilson, I.A., Schuitemaker, H., Klasse, P.J., Moore, J.P., Burton, D.R., Ward, A.B., and Sanders, R.W. (2016). An HIV-1 antibody from an elite neutralizer implicates the fusion peptide as a site of vulnerability. Nat Microbiol *2*, 16199. 10.1038/nmicrobiol.2016.199.
19. Falkowska, E., Le, K.M., Ramos, A., Doores, K.J., Lee, J.H., Blattner, C., Ramirez, A., Derking, R., van Gils, M.J., Liang, C.H., McBride, R., von Bredow, B., Shivatare, S.S., Wu, C.Y., Chan-Hui, P.Y., Liu, Y., Feizi, T., Zwick, M.B., Koff, W.C., Seaman, M.S., Swiderek, K., Moore, J.P., Evans, D., Paulson, J.C., Wong, C.H., Ward, A.B., Wilson, I.A., Sanders, R.W., Poignard, P., and Burton, D.R. (2014). Broadly neutralizing HIV antibodies define a glycan-dependent epitope on the prefusion conformation of gp41 on cleaved envelope trimers. Immunity *40*, 657-668. 10.1016/j.immuni.2014.04.009.

20. Kong, R., Xu, K., Zhou, T., Acharya, P., Lemmin, T., Liu, K., Ozorowski, G., Soto, C., Taft, J.D., Bailer, R.T., Cale, E.M., Chen, L., Choi, C.W., Chuang, G.Y., Doria-Rose, N.A., Druz, A., Georgiev, I.S., Gorman, J., Huang, J., Joyce, M.G., Louder, M.K., Ma, X., McKee, K., O'Dell, S., Pancera, M., Yang, Y., Blanchard, S.C., Mothes, W., Burton, D.R., Koff, W.C., Connors, M., Ward, A.B., Kwong, P.D., and Mascola, J.R. (2016). Fusion peptide of HIV-1 as a site of vulnerability to neutralizing antibody. Science *352*, 828-833. 10.1126/science.aae0474.
21. Schoofs, T., Barnes, C.O., Suh-Toma, N., Golijanin, J., Schommers, P., Gruell, H., West, A.P., Jr., Bach, F., Lee, Y.E., Nogueira, L., Georgiev, I.S., Bailer, R.T., Czartoski, J., Mascola, J.R., Seaman, M.S., McElrath, M.J., Doria-Rose, N.A., Klein, F., Nussenzweig, M.C., and Bjorkman, P.J. (2019). Broad and Potent Neutralizing Antibodies Recognize the Silent Face of the HIV Envelope. Immunity *50*, 1513-1529. 10.1016/j.immuni.2019.04.014.
22. Huang, J., Ofek, G., Laub, L., Louder, M.K., Doria-Rose, N.A., Longo, N.S., Imamichi, H., Bailer, R.T., Chakrabarti, B., Sharma, S.K., Alam, S.M., Wang, T., Yang, Y., Zhang, B., Migueles, S.A., Wyatt, R., Haynes, B.F., Kwong, P.D., Mascola, J.R., and Connors, M. (2012). Broad and potent neutralization of HIV-1 by a gp41-specific human antibody. Nature *491*, 406-412. 10.1038/nature11544.
23. Williams, L.D., Ofek, G., Schatzle, S., McDaniel, J.R., Lu, X., Nicely, N.I., Wu, L., Lougheed, C.S., Bradley, T., Louder, M.K., McKee, K., Bailer, R.T., O'Dell, S., Georgiev, I.S., Seaman, M.S., Parks, R.J., Marshall, D.J., Anasti, K., Yang, G., Nie, X., Tumba, N.L., Wiehe, K., Wagh, K., Korber, B., Kepler, T.B., Munir Alam, S., Morris, L., Kamanga, G., Cohen, M.S., Bonsignori, M., Xia, S.M., Montefiori, D.C., Kelsoe, G., Gao, F., Mascola, J.R., Moody, M.A., Saunders, K.O., Liao, H.X., Tomaras, G.D., Georgiou, G., and Haynes, B.F. (2017). Potent and broad HIV-neutralizing antibodies in memory B cells and plasma. Sci Immunol *2*. 10.1126/sciimmunol.aal2200.
24. Nelson, J.D., Brunel, F.M., Jensen, R., Crooks, E.T., Cardoso, R.M., Wang, M., Hessell, A., Wilson, I.A., Binley, J.M., Dawson, P.E., Burton, D.R., and Zwick, M.B. (2007). An affinity-enhanced neutralizing antibody against the membrane-proximal external region of human immunodeficiency virus type 1 gp41 recognizes an epitope between those of 2F5 and 4E10. J Virol *81*, 4033-4043. 10.1128/JVI.02588-06.
25. Kim, S., Chen, J., Cheng, T., Gindulyte, A., He, J., He, S., Li, Q., Shoemaker, B.A., Thiessen, P.A., and Yu, B. (2021). PubChem in 2021: new data content and improved web interfaces. Nucleic acids research *49*, D1388-D1395.
26. Yuen, G.J., Lou, Y., Thompson, N.F., Otto, V.R., Allsup, T.L., Mahony, W.B., and Hutman, H.W. (2001). Abacavir/Lamivudine/Zidovudin as a combined formulation tablet: bioequivalence compared with each component administered concurrently and the effect of food on absorption. The Journal of Clinical Pharmacology *41*, 277-288.
27. DiCenzo, R., Forrest, A., Squires, K.E., Hammer, S.M., Fischl, M.A., Wu, H., Cha, R., Morse, G.D., and Adult, A.C.T.G.P.S.T. (2003). Indinavir, efavirenz, and abacavir pharmacokinetics in human immunodeficiency virus-infected subjects. Antimicrob Agents Chemother *47*, 1929-1935. 10.1128/AAC.47.6.1929-1935.2003.
28. Pretorius, E., Klinker, H., and Rosenkranz, B. (2011). The role of therapeutic drug monitoring in the management of patients with human immunodeficiency virus infection. Therapeutic drug monitoring *33*, 265-274.
29. Sax, P.E., Pozniak, A., Montes, M.L., Koenig, E., DeJesus, E., Stellbrink, H.-J., Antinori, A., Workowski, K., Slim, J., and Reynes, J. (2017). Coformulated bictegravir, emtricitabine, and tenofovir alafenamide versus dolutegravir with emtricitabine and tenofovir alafenamide, for initial treatment of HIV-1 infection (GS-US-380–1490): a randomised, double-blind, multicentre, phase 3, non-inferiority trial. Lancet *390*, 2073-2082.
30. Moore, K.H., Yuen, G.J., Raasch, R.H., Eron, J.J., Martin, D., Mydlow, P.K., and Hussey, E.K. (1996). Pharmacokinetics of lamivudine administered alone and with trimethoprim-sulfamethoxazole. Clin Pharmacol Ther *59*, 550-558. 10.1016/S0009-9236(96)90183-6.
31. Calcagno, A., Gonzalez de Requena, D., Simiele, M., D'Avolio, A., Tettoni, M.C., Salassa, B., Orofino, G., Bramato, C., Libanore, V., Motta, I., Bigliano, P., Orsucci, E., Di Perri, G., and Bonora, S. (2013). Tenofovir plasma concentrations according to companion drugs: a cross-sectional study of HIV-positive patients with normal renal function. Antimicrob Agents Chemother *57*, 1840-1843. 10.1128/AAC.02434-12.

32. Boffito, M., Back, D.J., Blaschke, T.F., Rowland, M., Bertz, R.J., Gerber, J.G., and Miller, V. (2003). Protein binding in antiretroviral therapies. AIDS Res Hum Retroviruses *19*, 825-835. 10.1089/088922203769232629.
33. Ramachandran, G., Hemanthkumar, A.K., Kumaraswami, V., and Swaminathan, S. (2006). A simple and rapid liquid chromatography method for simultaneous determination of zidovudine and nevirapine in plasma. J Chromatogr B Analyt Technol Biomed Life Sci *843*, 339-344. 10.1016/j.jchromb.2006.06.014.
34. Yee, K.L., Sanchez, R.I., Auger, P., Liu, R., Fan, L., Triantafyllou, I., Lai, M.T., Di Spirito, M., Iwamoto, M., and Khalilieh, S.G. (2017). Evaluation of Doravirine Pharmacokinetics When Switching from Efavirenz to Doravirine in Healthy Subjects. Antimicrob Agents Chemother *61*. 10.1128/AAC.01757-16.
35. Khalilieh, S., Yee, K.L., Liu, R., Fan, L., Sanchez, R.I., Auger, P., Triantafyllou, I., Stypinski, D., Lasseter, K.C., Marbury, T., and Iwamoto, M. (2017). Moderate Hepatic Impairment Does Not Affect Doravirine Pharmacokinetics. J Clin Pharmacol *57*, 777-783. 10.1002/jcph.857.
36. Foca, M., Yogev, R., Wiznia, A., Hazra, R., Jean-Philippe, P., Graham, B., Britto, P., Carey, V.J., King, J., Acosta, E.P., Cressey, T.R., and Team, I.P. (2016). Rilpivirine Pharmacokinetics Without and With Darunavir/Ritonavir Once Daily in Adolescents and Young Adults. Pediatr Infect Dis J *35*, e271-274. 10.1097/INF.0000000000001214.
37. Aouri, M., Barcelo, C., Guidi, M., Rotger, M., Cavassini, M., Hizrel, C., Buclin, T., Decosterd, L.A., Csajka, C., and Swiss, H.I.V.C.S. (2017). Population Pharmacokinetics and Pharmacogenetics Analysis of Rilpivirine in HIV-1-Infected Individuals. Antimicrob Agents Chemother *61*. 10.1128/AAC.00899-16.
38. Janssen, P.A., Lewi, P.J., Arnold, E., Daeyaert, F., De Jonge, M., Heeres, J., Koymans, L., Vinkers, M., Guillemont, J., and Pasquier, E. (2005). In search of a novel anti-HIV drug: multidisciplinary coordination in the discovery of 4-[[4-[[4-[(1 E)-2-cyanoethenyl]-2, 6-dimethylphenyl] amino]-2-pyrimidinyl] amino] benzonitrile (R278474, rilpivirine). J Med Chem *48*, 1901-1909.
39. Gallant, J.E., Thompson, M., DeJesus, E., Voskuhl, G.W., Wei, X., Zhang, H., White, K., Cheng, A., Quirk, E., and Martin, H. (2017). Antiviral Activity, Safety, and Pharmacokinetics of Bictegravir as 10-Day Monotherapy in HIV-1-Infected Adults. J Acquir Immune Defic Syndr *75*, 61-66. 10.1097/QAI.0000000000001306.
40. Elliot, E., Amara, A., Jackson, A., Moyle, G., Else, L., Khoo, S., Back, D., Owen, A., and Boffito, M. (2016). Dolutegravir and elvitegravir plasma concentrations following cessation of drug intake. J Antimicrob Chemother *71*, 1031-1036. 10.1093/jac/dkv425.
41. Cattaneo, D., Minisci, D., Cozzi, V., Riva, A., Meraviglia, P., Clementi, E., Galli, M., and Gervasoni, C. (2017). Dolutegravir plasma concentrations according to companion antiretroviral drug: unwanted drug interaction or desirable boosting effect? Antivir Ther *22*, 353-356. 10.3851/IMP3119.
42. Cottrell, M.L., Hadzic, T., and Kashuba, A.D. (2013). Clinical pharmacokinetic, pharmacodynamic and drug-interaction profile of the integrase inhibitor dolutegravir. Clin Pharmacokinet *52*, 981-994. 10.1007/s40262-013-0093-2.
43. Huhn, G.D., Tebas, P., Gallant, J., Wilkin, T., Cheng, A., Yan, M., Zhong, L., Callebaut, C., Custodio, J.M., Fordyce, M.W., Das, M., and McCallister, S. (2017). A Randomized, Open-Label Trial to Evaluate Switching to Elvitegravir/Cobicistat/Emtricitabine/Tenofovir Alafenamide Plus Darunavir in Treatment-Experienced HIV-1-Infected Adults. J Acquir Immune Defic Syndr *74*, 193-200. 10.1097/QAI.0000000000001193.
44. Calcagno, A., Simiele, M., Motta, I., Mornese Pinna, S., Bertucci, R., D'Avolio, A., Di Perri, G., and Bonora, S. (2016). Elvitegravir/Cobicistat/Tenofovir/Emtricitabine Penetration in the Cerebrospinal Fluid of Three HIV-Positive Patients. AIDS Res Hum Retroviruses *32*, 409-411. 10.1089/aid.2015.0337.
