# Supplementary material for: Decoupling HIV-1 antiretroviral drug inhibition from plasma antibody activity to evaluate broadly neutralizing antibody therapeutics and vaccines
Source: Cell Rep Med. 2024 Aug 30;5(9):101702. doi: 10.1016/j.xcrm.2024.101702 (PMC11524982; doi:10.1016/j.xcrm.2024.101702)
Supplement: Document S2. Article plus supplemental information [file mmc2.pdf]

Cell Reports Medicine

Article

Decoupling HIV-1 antiretroviral drug inhibition from plasma antibody activity to evaluate broadly neutralizing antibody therapeutics and vaccines

Graphical abstract

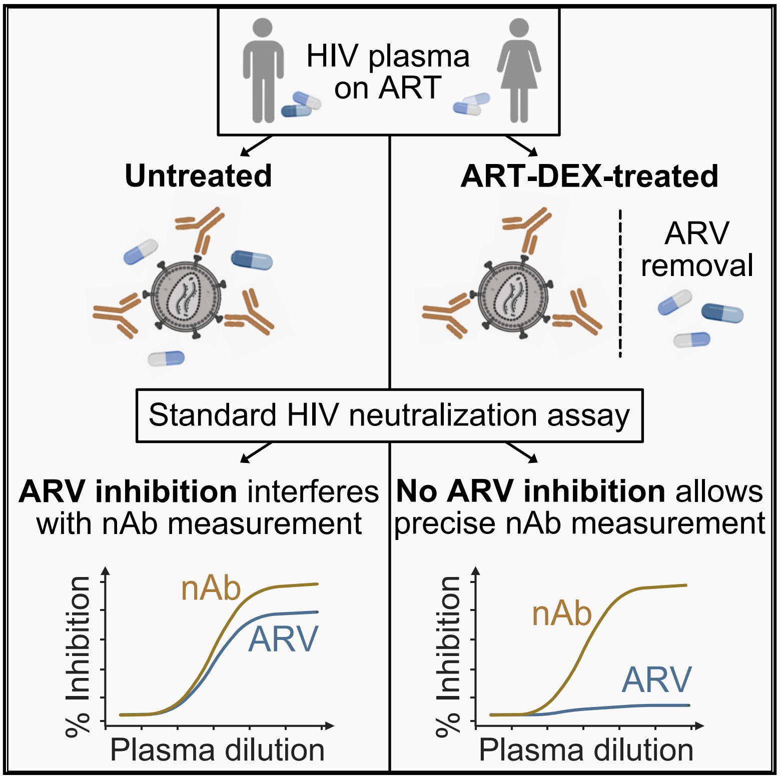

Authors

Magdalena Schwarzmüller, Cristina Lozano, Merle Schanz, ..., Huldrych F. Günthard, Alexandra Trkola, the Swiss HIV Cohort Study

Correspondence

trkola.alexandra@virology.uzh.ch

In brief

Schwarzmüller et al. report the development of the ART-DEX, a high-throughput method for removing antiretrovirals from the plasma of people with HIV allowing reliable monitoring of antibody-mediated neutralization with conventional neutralization assays. ART-DEX, used alone or with ART-resistant viruses, enhances the ability to evaluate bnAb activity in therapeutic and cure strategies.

Highlights

- ART-DEX is a high-throughput strategy to separate HIV drugs from plasma proteins
- ART-DEX is compatible with standard HIV-1 neutralization protocols
- ART-DEX advances monitoring of neutralizing antibody activity in people with HIV
- Implementing ART-DEX aids therapeutic evaluation of bnAbs and HIV cure strategies

Schwarzmüller et al., 2024, Cell Reports Medicine 5, 101702
September 17, 2024 © 2024 The Author(s). Published by Elsevier Inc.
https://doi.org/10.1016/j.xcrm.2024.101702

Cell Reports Medicine

CellPress
OPEN ACCESS

Article

Decoupling HIV-1 antiretroviral drug inhibition from plasma antibody activity to evaluate broadly neutralizing antibody therapeutics and vaccines

Magdalena Schwarzmüller,[1] Cristina Lozano,[1] Merle Schanz,[1] Irene A. Abela,[1,2] Silvan Grosse-Holz,[1] Selina Epp,[1,3] Martina Curcio,[1] Jule Greshake,[1] Peter Rusert,[1] Michael Huber,[1] Roger D. Kouyos,[1,2] Huldrych F. Günthard,[1,2] Alexandra Trkola,[1,4,*] and the Swiss HIV Cohort Study

[1]Institute of Medical Virology, University of Zurich, 8057 Zurich, Switzerland
[2]Department of Infectious Diseases and Hospital Epidemiology, University Hospital Zurich, 8091 Zurich, Switzerland
[3]Present address: Medical Microbiology, Cantonal Hospital Lucerne, 6000 Lucerne, Switzerland
[4]Lead contact
*Correspondence: trkola.alexandra@virology.uzh.ch
https://doi.org/10.1016/j.xcrm.2024.101702

SUMMARY

The development of broadly neutralizing antibody (bnAb)-based therapeutic HIV-1 vaccines and cure concepts depends on monitoring bnAb plasma activity in people with HIV (PWH) on suppressive antiretroviral therapy (ART). To enable this, analytical strategies must be defined to reliably distinguish antibody-based neutralization from drug inhibition. Here, we explore strategies that either utilize drug-resistant viruses or remove drugs from plasma. We develop ART-DEX (ART dissociation and size exclusion), an approach which quantitatively separates drugs from plasma proteins following pH-triggered release allowing accurate definition of antibody-based neutralization. We demonstrate that ART-DEX, alone or combined with ART-resistant viruses, provides a highly effective and scalable means of assessing antibody neutralization during ART. Implementation of ART-DEX in standard neutralization protocols should be considered to enhance the analytical capabilities of studies evaluating bnAb therapeutics and therapeutic vaccines, furthering the development of advanced ART and HIV-1 cure strategies.

INTRODUCTION

Current antiretroviral therapy (ART) is highly effective in suppressing HIV-1 replication to undetectable levels,[1,2] reversing $CD4^+$ T cell decline,[3,4] and reducing disease-related morbidity and mortality.[5–7] As a result, the life expectancy of people with HIV (PWH) treated early in the infection has extended to nearly that of people without HIV.[8–10] Typically, ART is provided as a combination therapy of drugs targeting one or more HIV-1 enzymes, with nucleoside reverse transcriptase inhibitors (NRTIs), non-nucleoside reverse transcriptase inhibitors (NNRTIs), integrase strand transfer inhibitors (INSTIs), and protease inhibitors (PIs) being widely used.[2] Over the past decades, a broad portfolio of therapeutics has become available for ART, most recently, potent entry and maturation inhibitors.[11,12] These versatile antiretrovirals (ARVs) remain essential to ensure uninterrupted, lifelong treatment options for HIV-1, providing the ability to switch between regimens as needed due to drug resistance, side effects, and drug-drug interactions.[13,14] The success of ART has also brought new perspectives to the development of HIV therapeutics. More potent drugs have allowed the reduction of combination therapy from triple therapy to double therapy and in controlled settings even to monotherapy.[15–19] The development of drugs with longer half-lives has enabled a shift to once-daily dosing,[20] with more recent developments toward long-acting injectables creating options for dosing currently every four to eight weeks.[21,22] Extending long-acting therapeutic options further, broadly neutralizing antibodies (bnAbs) are considered as the component of pre- and post-exposure prophylaxis, long-term ART, and HIV-1 cure strategies.[23–25] In this context, next to passive application of bnAbs, therapeutic bnAb-inducing vaccines are in development.[26] Combining bnAbs with conventional ART to ascertain full virus suppression is probed for the treatment of infants born to HIV-positive mothers (PedMAb1 trial, Pan African Clinical Trials Registry: PACTR202205715278722).[27] In PWH on ART, clinical trials are underway for preventive and therapeutic bnAb vaccines (clinicaltrials.gov: NCT05208125, NCT04985760, NCT06006546, and NCT04985760).[28]

While diverse bnAb treatment approaches move to advanced clinical testing in ART-treated PWH, test concepts still need to be established to allow accurate monitoring of bnAb levels and activity side-by-side with drug level monitoring[29] to enable efficacy evaluation across studies. Specific detection of distinct, passively administered bnAbs is possible, e.g., through binding to anti-idiotypic antibodies.[30] However, measuring bnAb neutralization activity in the therapeutic setting remains challenging because conventional ART efficiently blocks HIV in neutralization assays. Thus, standardizable neutralization assay

© 2024 The Author(s). Published by Elsevier Inc.
This is an open access article under the CC BY license (http://creativecommons.org/licenses/by/4.0/).

systems are needed that exclude interference of co-delivered drugs.

Standardization and quality assessment of HIV-1 plasma neutralization activity has been successfully implemented for HIV-1 vaccine trials using the TZM-bl-based neutralization assay in combination with envelope (Env) HIV-1 reporter pseudoviruses.[31,32] Clinical monitoring and efficacy evaluation depend on neutralization assay formats that provide high throughput, high accuracy, low cost, ease of use, and reproducibility. A neutralization assay format combining all these features and distinguishing between drug and antibody inhibition is therefore a critical need for the development of therapeutic vaccines and bnAb therapeutics. To fill this gap, we here explored modifications of the standard TZM-bl neutralization assay to devise a system that reliably records antibody-based neutralization in the presence of antiretrovirals.

RESULTS

ART-resistant HIV-1 vectors for distinguishing plasma antibody from antiretroviral drug inhibition

Our study aimed to develop a modified TZM-bl neutralization assay that is not confounded by the presence of ARVs in plasma and provides high comparability to the standard TZM-bl assay.[32] We refer to this as ART-free neutralization methods. A logical approach to minimize the inhibitory activity of ARVs is to use ART-resistant HIV-1.[33–35] Starting with the wild-type (WT) pseudotyping vector (pv), the reporter virus backbone HIV-1 NLlucAM based on HXB2 (referred to here as HIV^{pv}-WT),[36] we created different vector versions carrying known resistance mutations to reverse transcriptase (RT) and integrase (IN) inhibitors.[37] The effects of PIs were not considered in our vector design because they inhibit the formation of mature virus and thus successive rounds of infection. The single round of infection that pseudoviruses complete during a neutralization assay is therefore unaffected.

A general difficulty in creating drug-resistant HIV-1 is that mutations in the targeted enzymes commonly impact viral fitness.[38–41] This required us to monitor both the efficacy of the introduced mutations in conferring resistance to the drugs and the infectivity of the mutant viruses to ensure that sufficient infection capacity was maintained to allow their use in neutralization assays. We first focused on selected known key drug resistance mutations that were introduced in different combinations to HIV^{pv}-WT to create the multidrug-resistant (MDR) variants HIV^{pv}-MDR1 to MDR12 (Figure 1A). To reduce the impact of NRTIs, we included mutations K65R and M184V.[42–44] For NNRTI resistance, mutations K101P and Y181C[45] were added. Resistance to INSTIs often results in highly decreased integrase function.[46,47] We therefore tested different combinations of mutations to obtain resistance to INSTIs while maintaining sufficient integrase activity, including R263K, a mutation conferring low-level resistance to the second-generation INSTI dolutegravir (DTG),[48] and/or Q148H, a mutation associated with high resistance to first-generation INSTIs.[49,50] Mutations E138A, G140R, or S153Y were combined with the two major drug resistance mutations R263K and Q148H to achieve resistance against second-generation INSTIs including DTG and cabotegravir.[51,52] Next to these designed mutants, we cloned the *pol* gene of an HIV-1 isolate identified in routine clinical diagnostics with clinically documented resistance to four drug classes (NRTI, NNRTI, PI, and INSTI) into HIV^{pv} (HIV^{pv}-MDR13; Figure 1A).

As expected, there was a decrease in infectivity on TZM-bl cells compared to HIV^{pv}-WT for all MDR viruses tested (1.2–2.9 log fold loss in infectivity; Figures S1A and S1B). Constructs HIV^{pv}-MDR6, -MDR7, -MDR10, and -MDR12 proved not vital (Figures S1A and S1B). The remaining nine HIV^{pv}-MDR viruses showed sufficient infectivity and were compared to HIV^{pv}-WT for resistance to ARVs (Figures 1B and 1C). In this screen, the different pv backbones were examined as MuLV pseudotypes to analyze the inhibitory activity of ARVs. As only the Env molecule is derived from MuLV, all steps of the replication cycle, except for entry, are the same as with HIV-Env pseudoviruses. Therefore, the MuLV screening detects inhibition by most ARVs except HIV entry inhibitors. As representative drugs for the different inhibitor classes, the following ARVs were included in the screening: efavirenz (EFV) for NNRTI,[53,54] emtricitabine (FTC) for NRTI,[55,56] DTG as INSTI,[57,58] and darunavir (DRV) as PI.[59,60] Inhibitor dosing in these *in vitro* tests was adjusted to reflect the range of clinical drug levels reported in the literature (Figure 1B).[61–64]

The PI darunavir was included as non-active control in the HIV^{pv} system. It showed the expected lack of inhibitory activity against HIV^{pv}-WT over the full concentration range tested (Figures 1C, S1C, and S1D). Emtricitabine did not affect HIV^{pv} infectivity in dose ranges that are relevant for plasma neutralization assays (Figures 1C and S1C) and reached >50% inhibition of HIV^{pv}-WT only at concentrations >0.25 μM (Figure S1D). Resistance to EFV was achieved through mutations in the RT with K101P alone or in combination with Y181C. As described for *in vitro* DTG susceptibility,[65] R263K paired with Q148H partially reduced the inhibitory activity of DTG at clinically relevant doses (Figures 1C and S1C).

To mimic combination ART, we generated a drug cocktail containing all four drugs and probed it against the MDR viruses (Figures 1C and S1C). Among the designed HIV^{pv}-MDRs, HIV^{pv}-MDR8, carrying two RT mutations (K101P and Y181C) and two IN mutations (R263K and Q184H), was the best in conferring resistance to the cocktail. Notably, the clinical isolate-derived HIV^{pv}-MDR13 reached complete resistance against DTG and the highest overall resistance to the drug mix. Based on this, HIV^{pv}-MDR8 and -MDR13 were rated as the most promising candidates to follow up as ART-resistant pseudotyping vectors.

To probe the utility of HIV^{pv}-MDR8 and MDR13 for neutralization assays, we verified if their infectivity is sufficient for TZM-bl-based neutralization tests. For this, we generated pseudotypes carrying Env of MuLV and diverse HIV-1 strains (N = 13) and compared their infectivity on TZM-bl cells with HIV^{pv}-WT (Figure 1D; Table S1). Across all viruses, HIV^{pv}-MDR8 and -MDR13 pseudoviruses showed 1.8 log (SD 0.22) and 1.6 log (SD 0.15) reduced infectivity, respectively. Although low, all pseudoviruses except one, the CAP45 pseudovirus with the HIV^{pv}-MDR8 backbone, resulted in luciferase reporter production greater than 10-fold above background as recommended by the standard TZM-bl neutralization protocol (Table S1).[32]

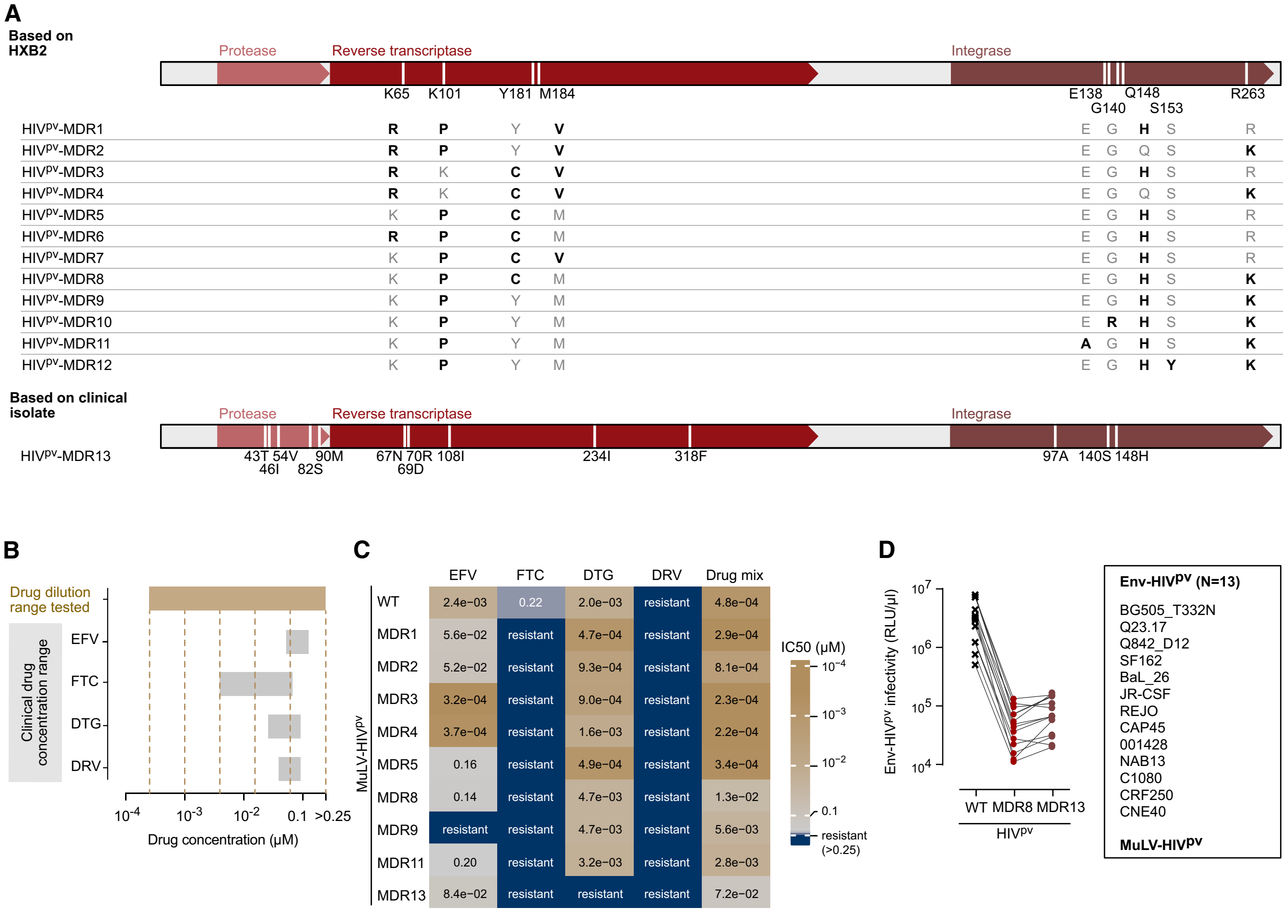

Figure 1. Creating ART-resistant HIV pseudovirus vectors

(A) Schematic of the HIV-1 *pol* gene and the combinations of mutations (bold) inserted into the HIV^{pv} backbone to achieve multidrug-resistant (MDR) variants.

(B) Schematic overview of plasma drug concentrations tested in (C) and published reference values (see Table S4). Values are adapted to a 1:100 dilution of plasma as used in the neutralization assay.

(C) Inhibition of MuLV-pseudotyped HIV^{pv}-WT and HIV^{pv}-MDR viruses by different ARVs at concentrations depicted in (B). Heatmap depicts mean inhibitory concentration 50 (IC50) values from two independent experiments. Drug mix: efavirenz (EFV), emtricitabine (FTC), dolutegravir (DTG), and darunavir (DRV) were combined and titrated.

(D) Infection of TZM-bl cells with serial dilutions of HIV^{pv}-WT, HIV^{pv}-MDR8, or HIV^{pv}-MDR13 pseudotyped with HIV-1 Env (*N* = 13) or MuLV. Infectivity is recorded as RLU and normalized to input of each virus stock. Mean RLU/μL titers from two independent experiments are shown. See also Figure S1.

CAP45 pseudovirus achieved luciferase levels only 9-fold above background but provided consistent NT50 data across duplicate measurements and was therefore included in the analysis.

Overall, utilizing ART-resistant HIV pseudovectors for neutralization assays proved feasible with some limitations, as fitness losses, in particular for multidrug resistance, can be substantial and none of the constructs reached full resistance.

Limitations of assessing HIV-1 antibody neutralization with the VSV pseudovirus system

We next considered pseudoviruses based on vesicular stomatitis virus (VSV) as a tool to prevent interference of ART in neutralization assays. VSV-based pseudoviruses have been used to study the entry and neutralization of various viruses, most recently severe acute respiratory syndrome coronavirus 2.[66–70] VSV, an enveloped single-stranded RNA virus of the *Rhabdoviridae* family, is completely distinct from HIV, both genetically and in terms of its replication cycle, rendering VSV intrinsically resistant to HIV-1 ARVs. As described previously, to generate entry-competent HIV-1 Env VSV pseudoviruses (VSV^{pv}), the cytoplasmic tail (CT) of Env must be deleted to allow Env incorporation into VSV particles (Figure 2A).[71] In analogy, the R peptide of MuLV Env[72,73] was deleted to produce infectious viral particles. Probing a panel of Env$^{\Delta CT}$ VSV^{pv} (HIV-1 Env$^{\Delta CT}$ (*N* = 13) and MuLV Env$^{\Delta R}$; Table S1), we confirmed that VSV pseudoviruses show no sensitivity against the tested ARVs (Figures 2B and S2A).

Considering that incorporation into a different virus particle and/or CT truncation may affect HIV-1 Env trimer conformation[74–76] and presentation of neutralizing epitopes, we next examined the sensitivity of the VSV^{pv} panel (*N* = 13) against a range of neutralizing monoclonal antibodies (mAbs; *N* = 30) and compared the sensitivity to matched HIV^{pv}-WT data (Figures

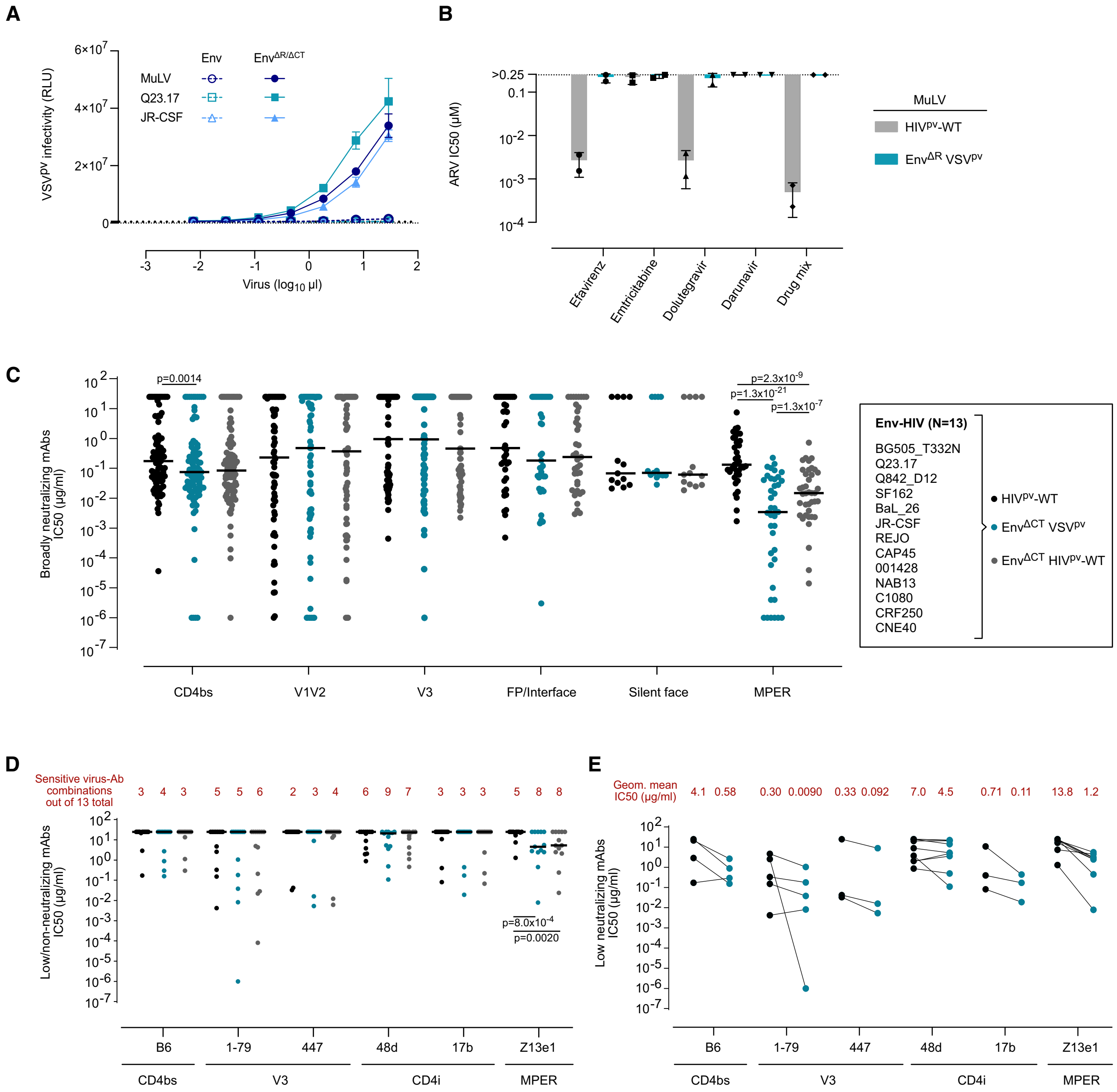

Figure 2. Differential sensitivity of VSV-based pseudoviruses to antibody neutralization

(A) Infectivity of VSV^{pv} viruses pseudotyped with full-length (open symbols) or CT-truncated (ΔCT) HIV-1 Env/R peptide-deleted (ΔR) MuLV (closed symbols). Infectivity of virus titrations was measured on TZM-bl cells and recorded as RLUs. Error bars indicate the standard deviation.

(B) Sensitivity of MuLV-pseudotyped VSV^{pv} viruses (blue) to ARVs with ARV concentrations as shown in Figure 1B. IC50 values of two independent experiments in a TZM-bl-based neutralization assay are shown. Error bars indicate the standard deviation. Data for HIV^{pv}-WT (black) from Figure 1C are depicted for comparison.

(C) Neutralization of a 13-virus panel in the context of HIV^{pv}-WT (black), Env$^{\Delta CT}$ VSV^{pv} (blue), and Env$^{\Delta CT}$ HIV^{pv}-WT (gray) by a panel of 30 bnAbs. Mean IC50 values of two independent experiments are shown. Significance thresholds are adjusted for multiple testing and indicated as follows: *$p < 0.05/18$, **$p < 0.01/18$, ***$p < 0.001/18$.

(D) Sensitivity of the different pseudoviruses to six low/non-neutralizing mAbs. Mean IC50 values of two independent experiments are shown. Significance thresholds are adjusted on multiple testing and indicated as follows: *$p < 0.05/12$, **$p < 0.01/12$, ***$p < 0.001/12$.

(E) IC50 comparison of VSV^{pv}-sensitive low/non-neutralizing mAb/virus combinations with HIV^{pv}-WT as depicted in (D). See also Figure S2 and Table S3.

2C and S2B–S2D; Tables S2 and S3). For most mAbs, neutralization activity was similar in both virus systems, but some mAb-VSV^{pv} combinations stood out as more sensitive. We noted a generally higher sensitivity of VSV^{pv} for mAbs targeting the CD4-binding site (CD4bs) and the membrane proximal external region (MPER) (Figures 2C and S2B–S2D). The most pronounced increase in sensitivity was observed for the tested MPER bnAbs 10E8, 4E10, and DH511.11P with 10E8 reaching a median 1,698-fold lower IC50 against the VSV^{pv} panel (Figure S2C). CD4bs bnAbs VRC01, b12, and CH235.12 but not N6, 1–18, PG04, and N49P7 were also highly affected by an increased sensitivity to VSV^{pv} (Figure S2D).

An increased neutralization sensitivity inflicted by the assay system is evidently of concern. We thus investigated if probing neutralization activity with VSV^{pv} would extend to classify low or non-neutralizing mAbs falsely as neutralizing. Comparing HIV^{pv}-WT with VSV^{pv} for six low/non-neutralizing mAbs (Table S2), we noted indeed some shifts toward sensitivity (Figure 2D). Overall, 8/54 virus-antibody combinations shifted from resistance to detectable neutralization when tested as HIV^{pv}-WT (IC50 > 25 μg/mL; Table S3). Additionally, some virus-antibody combinations that were already sensitive as HIV^{pv}-WT showed increased sensitivity in the context of VSV^{pv} (Figure 2E). Except for mAb 1–79 in combination with virus NAB13, the gain in potency was the highest for the MPER mAb Z13e1, mirroring what we observed for bnAbs and suggesting that MPER mAbs benefit from improved access to their epitope on Env$^{\Delta CT}$ VSV^{pv}. To evaluate if the increased sensitivity was caused by the truncation of the CT or the VSV-based vector itself, we probed Env$^{\Delta CT}$-pseudotyped viruses in the context of HIV^{pv}-WT (Figures 2C, 2D, and S2B–S2D). Overall, Env$^{\Delta CT}$ HIV^{pv}-WT showed the same trend toward increased neutralization sensitivity confirming the CT as a critical component in steering neutralization sensitivity for certain epitopes.[74–76] Taken together, our analysis suggests that VSV pseudoviruses may overestimate the neutralizing activity of certain antibody types and should be used with caution.

Low recovery of immunoglobulins after protein A/G bead separation

We next evaluated the purification of plasma immunoglobulins (Ig) using protein A/G[77–79] to separate antibodies from ARVs. For this, antibodies need to be separated with minimal loss to allow an accurate assessment of plasma neutralization activity. We therefore first assessed and optimized the Ig isolation procedure (Figures 3A and S3A–S3C). To this end, we prepared healthy donor plasma spiked with the CD4bs bnAb VRC01 (250 μg/mL in plasma) and tested its neutralization activity against Q23.17 HIV^{pv}-WT before and after Ig purification with protein A/G magnetic beads. For this, plasma and purified polyclonal antibodies were reconstituted to the same volume to allow direct comparison. Using a bead dose of 100 μL beads (50 μL protein A and 50 μL protein G) per 10 μL plasma and an incubation time of 30 min at room temperature, we observed a drastic loss in VRC01 neutralization activity (76.0% loss in neutralization activity [SD 18.4%] compared to untreated sample; Figure 3A). By elongating the incubation time to 24 h and increasing the amount of magnetic beads to 200 μL beads per 10 μL plasma (Figure 3A), the loss of neutralization activity was reduced from 76.0% (SD 18.4%) to 4.5% (SD 9.2%). However, while we found that protein A/G purification completely removes ARVs (Figure S3D), we consider the approach challenging for most high-throughput screens due to the hands-on time required, the large amount of beads, and their significant cost. Additionally, application of the method for clinical monitoring of antibody activity may require measuring of Ig yields as recovery across Ig subtypes may differ.

ART-DEX efficiently removes antiretrovirals from plasma

We next sought to implement a strategy that removes ARVs from plasma, rather than purifying antibodies. Current routinely used ARVs are small molecules with molecular weight (MW) of around 200–800 Da. Considering the larger size of Ig (MW~150 kDa), a straightforward separation by size exclusion would be in principle possible for ARVs except for antibody- or peptide-based inhibitors (e.g., ibalizumab or enfuvirtide as entry inhibitors).[11,80] However, most ARVs, in particular NNRTIs and PIs, are highly bound to plasma proteins and thus may not separate well by size exclusion (Table S4).[81–83] We probed this by spiking ARVs (EFV, FTC, DTG, and DRV) and VRC01 (individually or in combination with ARVs) into healthy donor plasma and performing size exclusion using commercial 96-well spin plates with a MW cutoff of 40 kDa. To monitor solely the effects of ARV inhibition, MuLV HIV^{pv}-WT was investigated, and to monitor effects on antibodies, VRC01 activity against Q23.17 HIV^{pv}-WT was measured. Although ARV activity in the spiked plasma against MuLV was reduced by 1.76 log (SD 0.043) after size exclusion, the residual drug activity was still substantial (Figure 3B).

We next made use of the fact that protein binding of drugs can be pH dependent.[84] We first investigated the effect of acidic pH in releasing ARVs from plasma proteins. Plasma samples were incubated in buffer solutions with pH ranging from 3.6 to 6.0 for 1 h followed by size-exclusion separation (Figure 4A). Incubation at pH to 3.6 for 1 h reduced ARV activity by an additional 0.51 log (SD 0.27) compared to size exclusion alone. Notably, the inhibitory activity of VRC01 was not affected by the pH treatment as the plasma spiked solely with VRC01 yielded similar neutralization activity against Q23.17 as after acid treatment and size exclusion (Figure 4A). Longer incubation at pH 3.6 did not further reduce the inhibitory activity of ARVs (Figure 4B).

To combat the residual ART inhibition after acidic treatment and size exclusion, we next considered an additional alkaline treatment as pH dependency may vary dependent on the plasma protein bound. Initial experiments showed that an alkaline step prior to the addition of plasma to TZM-bl cells should be avoided, as it may, at least at high doses of the buffer, impact neutralization assays and cell viability (Figures S3E and S3F). We therefore opted to first incubate plasma in alkaline pH followed by a size-exclusion step and then proceed with incubation at pH 3.6, again followed by size exclusion. Adding an alkaline treatment for 2 h at pH 10.0 was the most effective and led to a further decrease of ARV activity by 0.45 log (SD 0.018) compared to the acid treatment and size exclusion (Figure 4C). Neutralization capacity of VRC01 against Q23.17 was not affected. Incubation for more than 2 h at pH 10.0 did not further reduce the inhibitory activity of ARVs (Figure 4D).

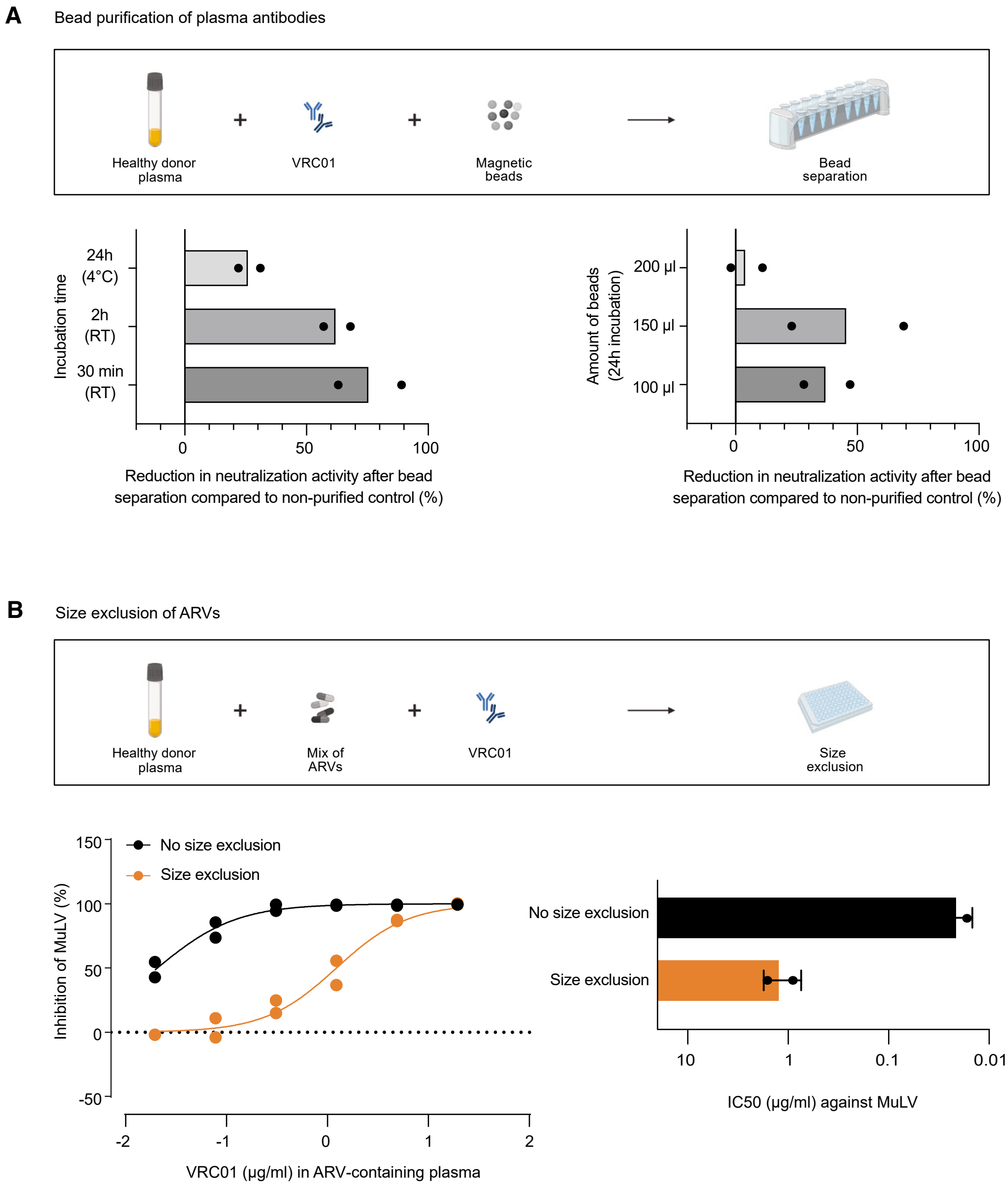

Figure 3. Strategies to separate ARVs from plasma antibodies

(A) Antibody separation by protein A/G beads: VRC01 (250 µg/mL) spiked into 10 µL healthy donor plasma was incubated with protein A/G magnetic beads before antibody elution. Neutralization activity against Q23.17 of the antibody-containing fraction was assessed in a HIV^{pv}-WT neutralization assay, and reduction of neutralization activity compared to untreated VRC01-spiked plasma was calculated. A fixed amount of beads (100 µL corresponding to 3,000 µg) was incubated at different times and temperatures (left) or varying amounts of beads were incubated for 24 h at 4°C before antibody elution (right).

(B) Size exclusion to separate ARVs from plasma: VRC01 (500 µg/mL) and the drug mix (EFV/FTC/DTG/DRV) were spiked into healthy donor plasma and size exclusion was performed. Inhibition of MuLV-pseudotyped HIV^{pv}-WT to reflect inhibition by ARVs was analyzed before and after size exclusion. MuLV inhibition curve (left) and corresponding IC50 values relating to VRC01 content (right) from two independent experiments are depicted. Error bars indicate the standard deviation. See also Figure S3.

We refer to this combined approach as ART dissociation and size exclusion (ART-DEX). We found that the final ART-DEX protocol—with combined alkaline (2 h at pH 10) and acid (1 h at pH 3.6) treatment with size-exclusion steps after each pH treatment—reduced the inhibitory activity of the tested mix of ARVs by 2.54 log (SD 0.17) resulting only in low-level residual ART inhibition of MuLV (IC50 = 17.22 µg/mL [SD 2.14 µg/mL], cutoff = 25 µg/mL). Considering that we probed comparatively high levels of drugs that represent maximal plasma concentrations in clinical use, the majority of ARV influence in plasma samples can be eliminated with this approach. As the procedure requires comparatively little handling and allows quantitative

CellPress
OPEN ACCESS

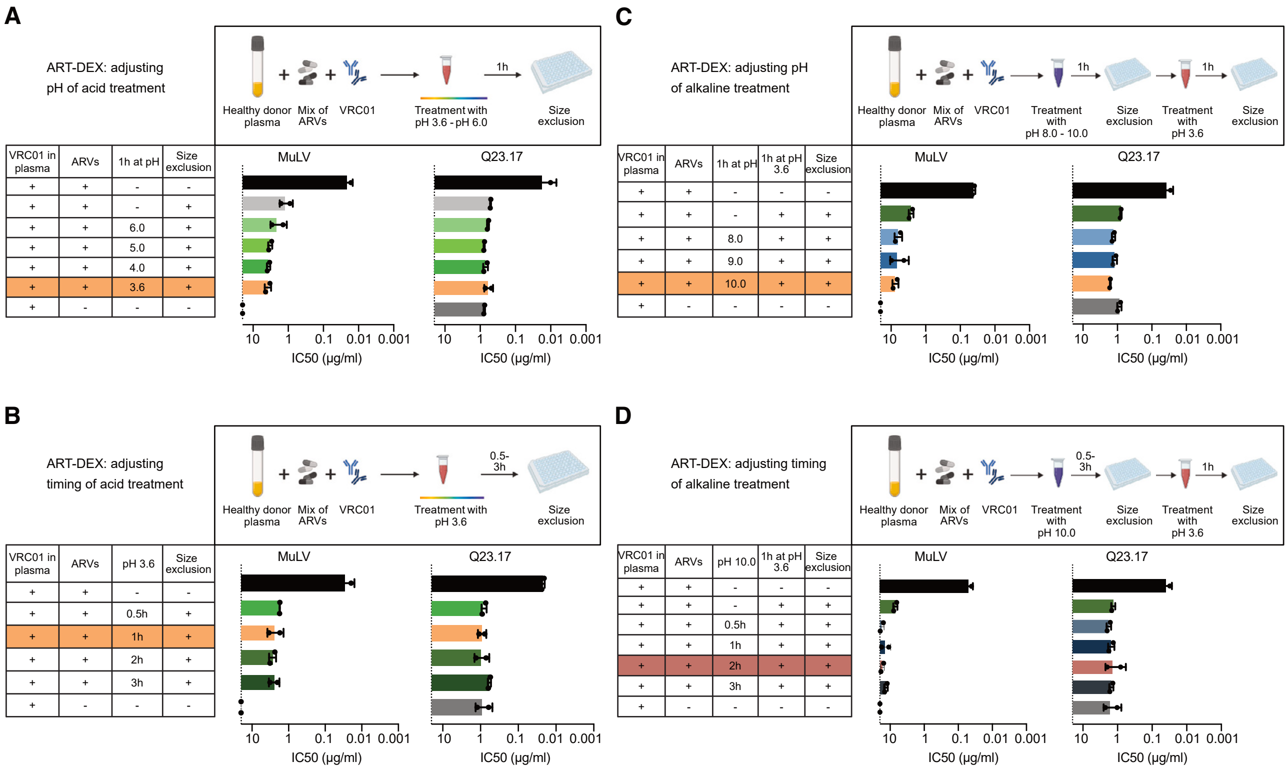

Figure 4. Dissociation and size exclusion to release and separate ARVs from plasma proteins

(A–D) Optimizing conditions for pH-dependent ARV dissociation from plasma proteins prior to size exclusion. Healthy donor plasma spiked with VRC01 (500 μg/mL) and the drug mix (EFV/FTC/DTG/DRV) was subjected to acidic (A and B) or combined alkaline/acidic (C and D) treatment before proceeding with size exclusion using spin plates. Optimal pH (A and C) and incubation times (B and D) were defined by testing separated antibody-containing plasma fractions for inhibition of MuLV and Q23.17-pseudotyped HIV^{pv}-WT. IC50 titers relate to VRC01 content. Data are from two independent experiments for each condition. Error bars indicate the standard deviation. In each optimization round shown in (A–C), the condition that was selected to proceed is highlighted in orange. The red shaded box in (D) indicates the final conditions of dissociation and size exclusion (ART-DEX) that were used thereafter (pH 10 for 2 h/size exclusion/pH 3.6 for 1 h/size exclusion). See also Figure S3.

retrieval of antibody activity at lower costs than Ig purification, it also holds promise for high-throughput applications.

Combining ART-DEX with ART-resistant HIV pseudovirus

In total, we established four different ART-free neutralization protocols to eliminate the influence of ARVs on antibody neutralization, two protocols based on resistant pseudovectors and two methods that attempt to separate ARVs from antibodies. We next compared the four approaches on plasma of PWH (*N* = 23) who were treated with different ARV combinations and for whom plasma drug level measurements were available through the biobank of the Zurich Primary HIV Infection study (ZPHI; Table S5).[85] In selecting these plasma samples, we aimed to investigate common drug combinations and sought to include also those that contained a second-generation INSTI in the drug regimen. The initial plasma dilution was set to 1:100. Neutralization titers against MuLV above 100 were defined as residual inhibitory activity of ARVs. Without treatment, 82.6% (19/23) of the samples inhibited MuLV in the standard TZM-bl/HIV^{pv}-WT assay at a median NT50 titer of 6,291 (Figure 5A). Both, protein A/G purification and VSV-based pseudoviruses, completely abolished ARV activity against MuLV as expected. HIV^{pv}-MDR8 pseudoviruses reduced activity against MuLV, but 39.1% (9/23) of the samples still showed residual MuLV inhibition. Analysis of the drug combinations with and without activity against MuLV HIV^{pv}-MDR8 indicated a full resistance to EFV (Figure 5B, left panel). Activity of ARV combinations containing second-generation INSTIs, DTG, bictegravir, or long-acting cabotegravir showed reduced activity compared to HIV^{pv}-WT, but still retained considerable residual inhibitory activity with a median NT50 titer of 1,209 (Figure 5B, middle panel). None of the other tested drug combinations inhibited MuLV HIV^{pv}-MDR8 (Figure 5B, right panel) consistent with the results obtained with single drugs spiked into healthy donor plasma (Figure 1C). HIV^{pv}-MDR13 outperformed -MDR8, with residual MuLV inhibition in 34.8% (8/23) samples with a median NT50 of 280 (Figure 5A). HIV^{pv}-MDR13 substantially reduced NT50 titers for ARV combinations containing EFV (median NT50 = 393) but also for second-generation INSTIs (Figure 5C). Using ART-DEX, 47.8% (11/23) of samples retained residual inhibition of MuLV, albeit at low NT50 titers with a median of 322 (Figure 5A). Analysis of drug combinations with residual MuLV inhibition after ART-DEX (Figure 5D) suggested that EFV, DTG, bictegravir, and elvitegravir were not fully removed.

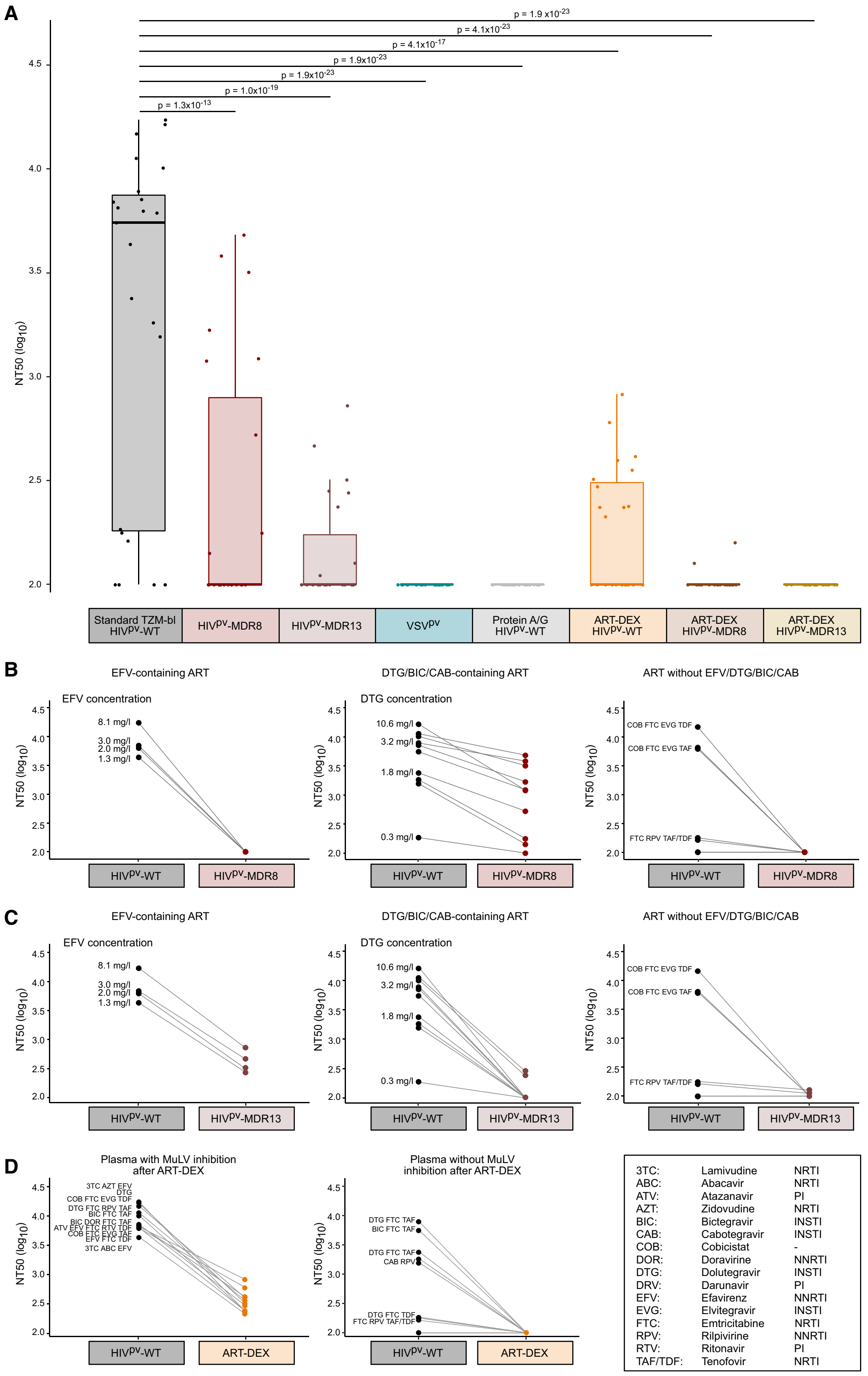

(legend on next page)

To resolve this residual ARV inhibition, we next combined ART-DEX with HIV^{pv}-MDR8 and HIV^{pv}-MDR13. The combination with HIV^{pv}-MDR13 completely eliminated the inhibitory activity of ARVs in all tested ZPHI plasma samples (*N* = 23) including those that contained high concentrations of second-generation INSTIs (Figure 5A). For an in-depth evaluation of the ability of the ART-DEX method to remove ARVs, we expanded the analysis to a total of 563 plasma samples from PWH on ART (Figure S4A; Table S5). We typically see a range of activity against MuLV in the standard TZM-bl assay with HIV^{pv}-WT, reaching from no inhibition at a plasma dilution of 1:100 to relatively high titers (>10,000). Among the probed plasma samples, 359/563 (63.8%) recorded with MuLV HIV^{pv}-WT inhibition. We subjected 108 of these samples with MuLV inhibition to ART-DEX in combination with HIV^{pv}-MDR13 (ART-DEX/HIV^{pv}-MDR13; Figure S4B). For 91.7% (99/108), ARV removal by ART-DEX with HIV^{pv}-MDR13 was successful. Neutralization activity measured against SF162 for plasma samples without MuLV inhibition after ART-DEX/HIV^{pv}-MDR13 (*N* = 80) showed activity in the range expected for this tier 1A HIV-1 strain (median NT50 = 321; Figure S4C).

Of note, during our study, the manufacturer released an updated version of the size-exclusion plates that required some protocol adaptations. We therefore verified the ART-DEX protocol for the updated plate version and confirmed the performance of ART removal from the ZPHI plasmas (Figure S4D). Results with the updated plate version yielded essentially the same results as the original plates, with 30.4% (7/23) of samples showing residual MuLV inhibition after ART-DEX at a median NT50 titer of 794 and the combination of ART-DEX and HIV^{pv}-MDR13 resulting in complete elimination of ARV inhibitory activity. In conclusion, we rate the combination of ART-DEX with HIV^{pv}-MDR13 as the most effective approach to distinguish antibody-based neutralization from drug inhibition.

ART-DEX combined with HIV^{pv}-MDR13 accurately captures plasma antibody neutralization

We next explored which of the ART-free neutralization methods most accurately reflects plasma neutralization activity measured in the standard TZM-bl/HIV^{pv}-WT assay. To this end, we utilized longitudinal plasma samples from two bnAb inducers, S5206-G5 and S51517, identified in the Swiss 4.5K screen[86,87] (Figure 6A) and assessed their neutralization capacity against a 14-virus panel (Table S1) with the different ART-free neutralization methods using the original version of the spin plates (Figure 6B; Table S6). For each bnAb inducer, six plasma samples were tested, two from off-ART and four from on-ART time points. Off-ART samples from S5206-G5 were from an ART interruption phase. Having first established the ability of the different methods to remove ARVs from plasma (Figure 5), we next focused on examining the accuracy of the methods in capturing plasma neutralization capacity equivalent to the standard TZM-bl assay. For this purpose, we selected plasma samples in which the influence of ARVs on the standard TZM-bl neutralization assay was low: MuLV HIV^{pv}-WT was inhibited by only a fraction of the longitudinal on-ART time points tested for donor S5206-G5 (1/4) and donor S51517 (2/4), and then at low titers. This overall low effect of ARVs over the longitudinal plasma time course allowed direct comparison of the ART-free neutralization methods with the neutralization titers obtained by the standard TZM-bl neutralization assay, providing a means to assess the quality of the evaluated ART-free techniques for accurately recording bnAb activity. All seven strategies (the five individual methods and the combined ART-DEX with HIV^{pv}-MDR8 or HIV^{pv}-MDR13) eliminated the ART influence. Despite using the optimized bead protocol, protein A/G purification was surprisingly poor and led to a massive loss in neutralization activity. All other methods captured the neutralization activity well (Figure 6B; Table S6). Notably, VSV^{pv} showed an enhanced neutralization sensitivity, which was particularly striking for JR-CSF (Figure S5A). Modest increases in neutralization sensitivity were also notable for HIV^{pv}-MDR8 alone or in combination with ART-DEX, but these were not uniform and restricted to few plasma-virus combinations. Overall, the variation in neutralization titers compared to the standard TZM-bl assay was within the expected range of assay variation and only showed notable differences for protein A/G purification and VSV^{pv}-based assays (Figure S5B).

Linear regression analysis considering only samples off-ART underlined the differential performance of the ART-free methods and signified ART-DEX as the method most accurately capturing neutralization activity as recorded by the standard method reaching $R^2 = 0.89$ (Figures 6C and S5C). Both, protein A/G purification ($R^2 = 0.13$) due to the massive loss in activity and VSV^{pv} ($R^2 = 0.53$) due to the potential shifts in neutralization for certain antibody types, need to be used with caution. Approaches with HIV^{pv}-MDR8 or HIV^{pv}-MDR13 alone ($R^2 = 0.67$ and $R^2 = 0.64$, respectively) or ART-DEX/HIV^{pv}-MDR8 ($R^2 = 0.53$) performed well but did not reach the level of agreement seen with ART-DEX alone or ART-DEX/HIV^{pv}-MDR13 ($R^2 = 0.84$). We again verified that the updated version of the spin plates showed the same performance (Figures S5D and S5E).

Due to the high correlation with the standard TZM-bl assay (Figures 6C and S5C) as well as the complete removal of inhibitory activity of ARVs (Figure 5A), we ranked ART-DEX/HIV^{pv}-MDR13 as the best-performing ART-free method that

Figure 5. Verifying the capacity of ARV removal strategies in plasma of ART-treated PWH

(A) Plasma samples from PWH (*N* = 23) with different ART combination regimen were tested for MuLV inhibition using the standard TZM-bl assay or the indicated ART-free strategies. The effective primary plasma dilution during culture was 1:100 (after combination of plasma, pseudovirus, and TZM-bl cells). NT50 titer against MuLV derived from two independent experiments is shown. Boxplots represent median with the middle line, upper and lower quartiles with the box limits, and 1.5 x interquartile ranges with the whiskers. Significance thresholds are adjusted for multiple testing and indicated as follows: *$p < 0.05/7$, **$p < 0.01/7$, ***$p < 0.001/7$.

(B and C) Dissecting resistance of HIV^{pv}-MDR8 (B) and HIV^{pv}-MDR13 (C) to different ART regimen in NT50 data depicted in (A). Clinically determined drug concentrations for efavirenz and dolutegravir are indicated.

(D) Comparison of drug regimens with and without residual ARV inhibition following ART-DEX as depicted in (A). See also Figure S4, Table S5.

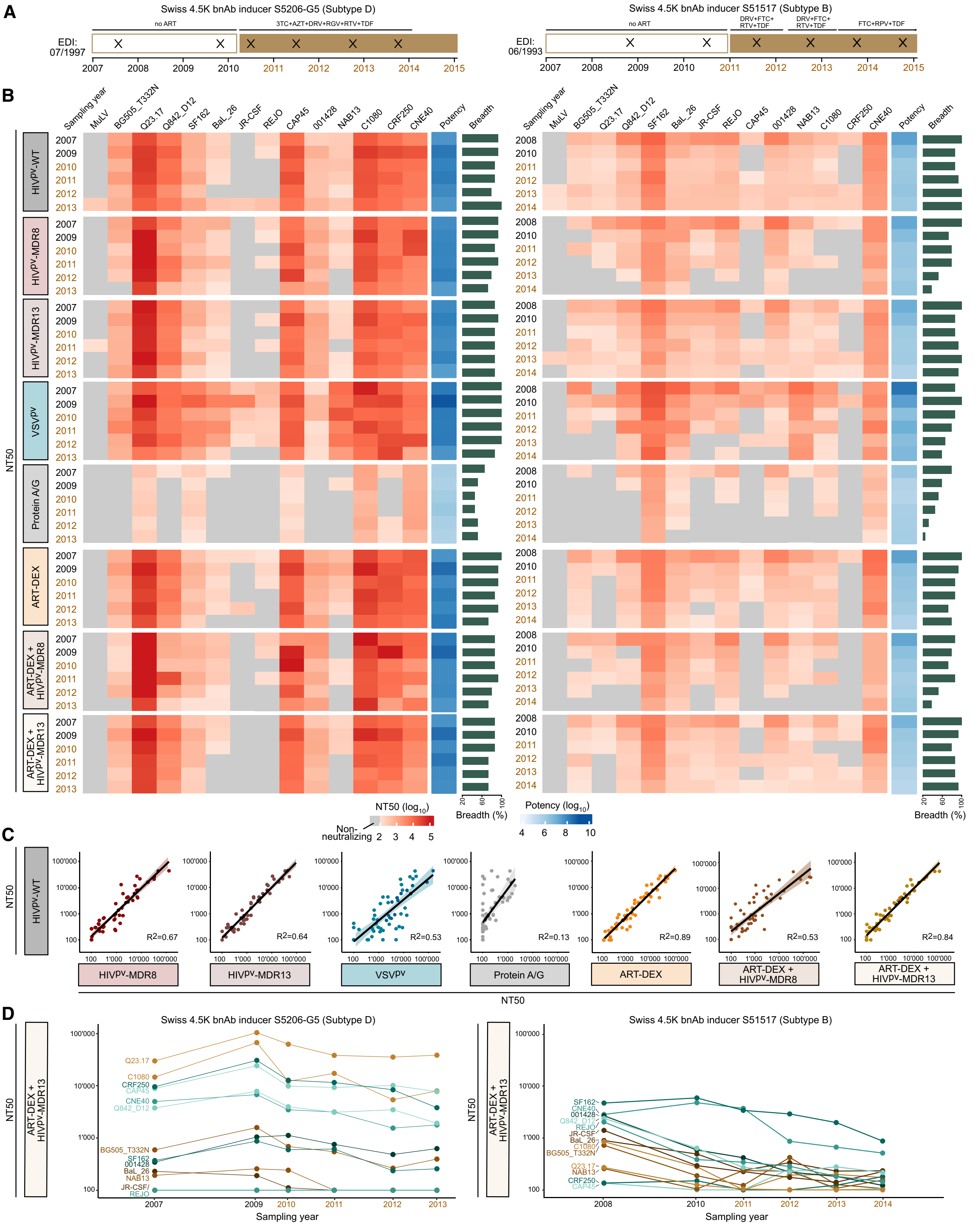

(legend on next page)

allows accurate longitudinal assessment of antibody-based neutralization in PWH on ART (Figure 6D).

DISCUSSION

Technical solutions that permit monitoring of antibody neutralization in ART-containing plasma will be a critical component to move bnAb therapeutics forward and establish their clinical use beyond carefully controlled experimental studies with analytical treatment interruption (ATI).[88–90] Measuring bnAb activity in HIV cure applications will be particularly important to ensure that only individuals who have responded sufficiently to bnAb treatment or vaccination undergo ATI.

Here, we aimed to develop strategies to monitor antibody-based inhibition in plasma with HIV enzyme-targeting ARVs. We sought methods that reliably quantify HIV antibody neutralization, are scalable for higher throughput, and cost-effective. We pursued four strategies, focusing on ART-resistant viral vectors or separating ARVs from plasma.

Establishing ARV-resistant viruses for neutralizing assays is attractive because, if successful, this approach will allow direct testing of ARV-containing plasma for neutralizing antibody activity using the conventional TZM-bl assay with minimal modification and no additional plasma processing. However, both pseudovirus systems we studied, the ART-resistant HIV^{pv} and VSV^{pv}, showed limitations. Achieving full resistance to HIV enzyme-targeting ARVs while maintaining sufficient infection capacity of *pol*-mutated HIV^{pv} proved to be challenging. In creating suitable multidrug-resistant HIV^{pv}, we thus aimed for a compromise between sufficient infectivity at the expense of residual ART sensitivity. This balance was best achieved for two constructs, one engineered to harbor selected drug resistance mutations and the other containing the entire *pol* gene of a multidrug-resistant HIV isolate discovered in routine resistance testing. Both showed good but not complete resistance to all categories of ARVs tested. Therefore, their use should be determined based on the specific ART context of the study population. With increasing options of more potent drugs arising, multi-resistant HIV pseudovectors will need continuous adaptation. Realistically, given the decrease in infectivity observed with each added resistance mutation, the chances of creating a single multi-resistant HIV backbone that confers resistance to all major ARVs must be considered limited.

While VSV^{pv} is inherently resistant to HIV-specific ARVs, the drawback of this system lies in alterations in HIV-1 Env conformation in the context of VSV particles that induced shifts in antibody-based neutralization particularly noticeable for MPER- and CD4bs-directed mAbs. These shifts were linked to truncation of the Env CT needed for sufficient HIV Env incorporation into VSV. This truncation can reduce trimer stability, alternate epitope exposure, and/or induce changes in fusion kinetics,[91–93] which may be particularly advantageous for epitopes such as the MPER domain, which has known low accessibility.[94,95] Unilateral shifts in neutralization activity, as we observed for VSV pseudovirus neutralization with plasma samples from bnAb inducers, are particularly problematic to control for when assessing polyclonal responses. Therefore, VSV-based systems should be used with caution.

To separate ARVs from antibodies in plasma, we first assessed Ig purification using protein A/G separation, a method that has been widely used.[77–79] As others, we found protein A/G purification highly efficient in separating antibodies from ARVs.[77,96,97] In our setup, this was however accompanied by a marked loss in antibody recovery. As we showed, increasing protein A/G bead concentration can improve antibody recovery from plasma but will increase costs substantially. Protein A/G protocols with higher antibody binding capacity than we observed here can probably be developed. However, given the results of the present study, the potential for low recovery needs to be controlled. This requires that Ig concentrations before and after the purification be measured, and fixed antibody doses be used for neutralization assays.[96] Overall, we consider antibody purification as highly effective in separating antibodies from ARVs but challenging for high-throughput testing and clinical monitoring due to sample handling and the need to control for Ig recovery.

When purifying Ig from plasma, it is important to consider that the recovery of antibody subtypes may vary. Purified Ig may have a different composition than unprocessed plasma, which could affect the measurement of neutralization activity. In the final approach, ART-DEX, we therefore developed a strategy to remove ARVs from plasma while leaving the plasma antibodies untouched. This approach required the dissociation of ARVs from plasma proteins to allow the removal of the free drugs by size-exclusion separation. ARVs, especially NNRTIs and PIs, are highly bound to plasma proteins such as albumin or α1-acid glycoprotein.[81,98,99] Notably, drug-protein binding has been shown to be pH dependent[84] and may be caused by differential ionization of the drugs affecting binding,[100] pH-dependent changes in affinity,[101,102] or pH-dependent conformational changes of the plasma protein affecting the accessibility of binding sites for the drugs.[103–105] We exploited this pH dependence to release drugs from plasma proteins by subjecting plasma samples to alkaline and acidic pH. Size-exclusion separation

Figure 6. ART-DEX combined with HIV^{pv}-MDR13 ranks best in monitoring *in vivo* neutralization capacity

(A) Two bnAb inducers were assessed for neutralization activity over several years of follow-up, each encompassing two time points without ART (clear boxes) and four time points with ART (brown boxes). EDIs (estimated date of infection) and specific ART combinations at sampling time points (crosses) are listed.

(B) Plasma neutralization activity of bnAb inducer S5206-G5 (left) and S51517 (right) was assessed with a 13-virus panel and MuLV as control using the different ART-free neutralization procedures and the standard TZM-bl assay. The effective primary plasma dilution during culture was 1:100 (after combination of plasma, pseudovirus, and TZM-bl cells). Heatmaps of the respective NT50 titers for each virus/plasma/method combination with potency (geometric mean NT50) and breadth (%) across HIV pseudoviruses are depicted.

(C) Correlation analysis of NT50 titers based on HIV^{pv}-WT and the ART-free neutralization methods using solely data from the two sampling time points before ART initiation.

(D) NT50 titers against the 13-virus panel for ART-DEX/HIV^{pv}-MDR13 (see B) are depicted as line plots over time. See also Figure S5 and Table S6.

following pH treatment successfully reduced the ARV burden, with only the most potent drugs showing residual inhibition. Given the effect of both alkaline and acidic pH treatment on ARV release from plasma proteins observed during ART-DEX, a systematic evaluation of which drugs are preferentially released at low or high pH would be of interest for future ART-DEX studies. Acid treatment, which is part of ART-DEX, is a common strategy to dissociate immune complexes.[106–108] We envision the utility of ART-DEX primarily for specimen on ART where, in the absence of viral replication, immune complex formation with Env antigens will not occur. However, HIV antibodies are known to cross-react with a number of autoantigens, and the effect of ART-DEX on these immune complexes must be considered. Importantly, ART-DEX processing did not affect neutralizing antibody activity. This was demonstrated for neutralization by VRC01 and four polyclonal plasma samples from bnAb inducers off-ART analyzed with and without the ART-DEX protocol. Therefore, while we cannot rule out the possibility that some neutralization enhancing or reducing effects of ART-DEX may be seen when larger numbers of samples or mAbs are analyzed, the overall change in antibody neutralization would be expected to be small. Comparing all methods on the same set of plasma samples collected at time points without ART, ART-DEX treatment yielded the highest agreement with the standard TZM-bl neutralization assay, underlining the validity of using ART-DEX for monitoring neutralization activity. We rate ART-DEX as the most straightforward approach we tested, as neither antibody loss, antibody inactivation, nor distortion of neutralization patterns occur (Table S7).

Collectively, all ART-free strategies we explored in the present study can be used to eliminate ARV influence in HIV neutralization tests when tightly controlled. Their utility will however differ depending on sample size, ARV combination, dosing, and research question studied. Overall, we consider the combined use of drug-resistant HIV pseudoviruses, such as HIV^{pv}-MDR13, with ART-DEX of the highest utility for monitoring of HIV antibody neutralization under ART (Table S7). As we demonstrate, the residual ARV activity that may occur in both systems individually is low and can be eliminated when the two approaches are combined (Figure 5; Figure S4). We noted a low-level residual inhibitory activity in some EFV-containing samples after ART-DEX/HIV^{pv}-MDR13 (Figure S4B). As this occurred in samples with high ARV inhibition before ART-DEX clean-up, additional clean-up rounds or use of the EFV-resistant HIV^{pv}-MDR8 can be considered to resolve this. Overall, ART-DEX/HIV^{pv}-MDR13 has a high potential to support clinical monitoring needed to advance therapeutic bnAb applications and vaccines from experimental studies to clinical use. More specifically, ART-DEX protocols can be used not only to screen for bnAb activity in natural HIV infection but also to monitor bnAb activity during therapeutic interventions, including bnAb therapy, cure approaches, or vaccination while on ART. Using the ART-free strategy, bnAb activity can be analyzed more easily prior to ATI and help guide further interventions.

While our study focused on the application of ART-DEX in the context of HIV, ART-DEX is not an HIV-specific approach and can be applied in the assessment of antibody responses concurrent with antiviral treatment in other viral diseases or for other biological tests that require the removal of small drugs from biological fluids.

Limitations of the study

Our study presents ART-DEX as an effective strategy to overcome antiretroviral interference in measuring HIV-neutralizing antibody activity in the plasma of PWH, but ART-DEX also has limitations that need to be considered. A primary challenge remains that ART-DEX does not fully remove ARVs. Some highly potent ARVs can therefore show residual activity, requiring the combined use of ART-DEX with ART-resistant virus backbones. Future studies should aim to resolve this limitation by installing a second ART-DEX clean-up round. Although we did not observe changes in antibody neutralization after treatment with ART-DEX, it is possible that neutralization enhancing or neutralization reducing effects may only become evident when analyzing larger numbers of samples.

STAR★METHODS

Detailed methods are provided in the online version of this paper and include the following:

- KEY RESOURCES TABLE
- RESOURCE AVAILABILITY
  - Lead contact
  - Materials availability
  - Data and code availability
- EXPERIMENTAL MODEL AND STUDY PARTICIPANT DETAILS
  - Clinical specimen
  - Cell lines
- METHOD DETAILS
  - Antiretrovirals
  - Antibodies
  - Virus envelope proteins
  - HIV-1 ART-resistant pseudovector
  - HIV-based Env-pseudotype viruses
  - VSV-based Env-pseudotype viruses
  - Neutralization assay
  - Purification of antibodies from plasma
  - ART-DEX: Dissociation and size EXclusion
  - Cell viability assay
- QUANTIFICATION AND STATISTICAL ANALYSIS
  - Statistical analyses
  - Programs

SUPPLEMENTAL INFORMATION

Supplemental information can be found online at https://doi.org/10.1016/j.xcrm.2024.101702.

CONSORTIA

Members of the Swiss HIV Cohort Study are: Irene Alma Abela, Karoline Aebi-Popp, Alexia Anagnostopoulos, Manuel Battegay, Enos Bernasconi, Dominique Laurent Braun, Heiner Bucher, Alexandra Calmy, Matthias Cavassini, Angela Ciuffi, Günter Dollenmaier, Matthias Egger, Luisa Elzi, Jan Fehr, Jacques Fellay, Hansjakob Furrer, Christoph Fux, Günthard Huldrych Fritz, Anna Hachfeld, David Haerry, Barbara Hasse, Hans Hirsch, Matthias Hoffmann, Irene Hösli, Michael Huber, David Jackson-Perry, Christian Kahlert, Laurent Kaiser, Olivia Keiser, Thomas Klimkait, Roger Dimitri Kouyos, Helen Kovari, Katharina Kusejko, Niklaus Labhardt, Karoline Leuzinger, Begogna Martinez de Tejada Catja Marzolini, Karin Jutta Metzner, Nicolas Müller, Johannes Nemeth, Dunja Nicca, Julia Notter, Paolo Paioni, Giuseppe Pantaleo,

Matthieu Perreau, Andri Rauch, Luisa Salazar-Vizcaya, Patrick Schmid, Roberto Speck, Marcel Stöckle, Philip Tarr, Alexandra Trkola, Gilles Wandeler, Maja Weisser, and Sabine Yerly.

ACKNOWLEDGMENTS

This was work was funded by Swiss National Science Foundation (SNSF) grant SNF 3147308_201266 (A.T.) and a Gilead Sciences Swiss Fellowship 2023 (A.T.). We thank David Pedítto, Jacqueline Weber, Michèle Sickmann, Cyrille Niklaus, and Gabriela Ziltener for technical assistance. We thank the participants of the ZPHI and the SHCS for their commitment and Dominique L. Braun, Christina Grube, and clinical staff of the ZPHI and SHCS for patient care.

AUTHOR CONTRIBUTIONS

M. Schwarzmüller and A.T. conceived and designed the study. M. Schwarzmüller, M. Schanz, I.A.A., and P.R. designed the experiments and analyzed the data. M. Schwarzmüller, C.L., S.E., S.G.-H., M.C., and J.G. performed the experiments. R.D.K. and H.F.G. managed patient cohorts, contributed patient samples, and analyzed patient-related data. M.H. analyzed *pol* resistance. M. Schwarzmüller and A.T. wrote the paper, which all co-authors commented on.

DECLARATION OF INTERESTS

A.T. has received unrelated unrestricted research grants from the SNSF, Bill and Melinda Gates Foundation, Gilead Sciences, Novartis Biomedical Research Foundation, University of Zurich (UZH) Foundation, UZH Clinical Research Priority Program, the SHCS, honoraria from Roche Diagnostics and the Institute for biomedical research Bellinzona for consultant and scientific board activity, and directs the Swiss National Reference Center for Retroviruses together with M.H. H.F.G. has received unrelated unrestricted research grants from the SNSF, the SHCS, Yvonne Jacob Foundation, University of Zurich Clinical Research Priority Program, Systems.X, the National Institutes of Health, Gilead Sciences, and Roche. H.F.G. has further received personal fees from Merck, Gilead Sciences, ViiV, Janssen, GSK, Johnson and Johnson, and Novartis for consultancy or DSMB membership and a travel grant from Gilead. M.H. directs the Swiss National Reference Center for Retroviruses and has received unrelated unrestricted research grant from the UZH Clinical Research Priority Program, the SNSF, the SHCS, and the ETH PHRT. I.A.A. has received honoraria from MSD and Sanofi, a travel grant from Gilead Sciences, a grant from ProMedica Foundation, and she is member of the EKIF (Eidgenössische Kommission für Impffragen) of the Federal Office of Public Health. R.D.K. has received grants from SNSF, the National Institutes of Health, and Gilead Sciences.

DECLARATION OF GENERATIVE AI AND AI-ASSISTED TECHNOLOGIES IN THE WRITING PROCESS

During the preparation of this work, the authors used DeepL Translate, DeepL Write, and ChatGPT 3.5 for language editing. After using these tools, the authors reviewed and edited the content as needed and take full responsibility for the content of the publication.

Received: February 26, 2024
Revised: May 2, 2024
Accepted: August 6, 2024
Published: August 30, 2024

REFERENCES

1. Scherrer, A.U., Traytel, A., Braun, D.L., Calmy, A., Battegay, M., Cavassini, M., Furrer, H., Schmid, P., Bernasconi, E., Stoeckle, M., et al. (2022). Cohort Profile Update: The Swiss HIV Cohort Study (SHCS). Int. J. Epidemiol. *51*, 33–34j. https://doi.org/10.1093/ije/dyab141.

2. Gandhi, R.T., Bedimo, R., Hoy, J.F., Landovitz, R.J., Smith, D.M., Eaton, E.F., Lehmann, C., Springer, S.A., Sax, P.E., Thompson, M.A., et al. (2023). Antiretroviral Drugs for Treatment and Prevention of HIV Infection in Adults: 2022 Recommendations of the International Antiviral Society-USA Panel. JAMA *329*, 63–84. https://doi.org/10.1001/jama.2022.22246.

3. Guihot, A., Bourgarit, A., Carcelain, G., and Autran, B. (2011). Immune reconstitution after a decade of combined antiretroviral therapies for human immunodeficiency virus. Trends Immunol. *32*, 131–137. https://doi.org/10.1016/j.it.2010.12.002.

4. Le, T., Wright, E.J., Smith, D.M., He, W., Catano, G., Okulicz, J.F., Young, J.A., Clark, R.A., Richman, D.D., Little, S.J., and Ahuja, S.K. (2013). Enhanced CD4+ T-cell recovery with earlier HIV-1 antiretroviral therapy. N. Engl. J. Med. *368*, 218–230. https://doi.org/10.1056/NEJMoa1110187.

5. Palella, F.J., Jr., Baker, R.K., Moorman, A.C., Chmiel, J.S., Wood, K.C., Brooks, J.T., and Holmberg, S.D.; HIV Outpatient Study Investigators (2006). Mortality in the highly active antiretroviral therapy era: changing causes of death and disease in the HIV outpatient study. J. Acquir. Immune Defic. Syndr. *43*, 27–34. https://doi.org/10.1097/01.qai.0000233310.90484.16.

6. d'Arminio Monforte, A., Sabin, C.A., Phillips, A., Sterne, J., May, M., Justice, A., Dabis, F., Grabar, S., Ledergerber, B., Gill, J., et al. (2005). The changing incidence of AIDS events in patients receiving highly active antiretroviral therapy. Arch. Intern. Med. *165*, 416–423. https://doi.org/10.1001/archinte.165.4.416.

7. HIV-CAUSAL Collaboration; Ray, M., Logan, R., Sterne, J.A.C., Hernández-Díaz, S., Robins, J.M., Sabin, C., Bansi, L., van Sighem, A., de Wolf, F., et al. (2010). The effect of combined antiretroviral therapy on the overall mortality of HIV-infected individuals. AIDS *24*, 123–137. https://doi.org/10.1097/QAD.0b013e3283324283.

8. Gueler, A., Moser, A., Calmy, A., Günthard, H.F., Bernasconi, E., Furrer, H., Fux, C.A., Battegay, M., Cavassini, M., Vernazza, P., et al. (2017). Life expectancy in HIV-positive persons in Switzerland: matched comparison with general population. AIDS *31*, 427–436. https://doi.org/10.1097/QAD.0000000000001335.

9. May, M.T., Gompels, M., Delpech, V., Porter, K., Orkin, C., Kegg, S., Hay, P., Johnson, M., Palfreeman, A., Gilson, R., et al. (2014). Impact on life expectancy of HIV-1 positive individuals of CD4+ cell count and viral load response to antiretroviral therapy. AIDS *28*, 1193–1202. https://doi.org/10.1097/QAD.0000000000000243.

10. Samji, H., Cescon, A., Hogg, R.S., Modur, S.P., Althoff, K.N., Buchacz, K., Burchell, A.N., Cohen, M., Gebo, K.A., Gill, M.J., et al. (2013). Closing the gap: increases in life expectancy among treated HIV-positive individuals in the United States and Canada. PLoS One *8*, e81355. https://doi.org/10.1371/journal.pone.0081355.

11. Emu, B., Fessel, J., Schrader, S., Kumar, P., Richmond, G., Win, S., Weinheimer, S., Marsolais, C., and Lewis, S. (2018). Phase 3 Study of Ibalizumab for Multidrug-Resistant HIV-1. N. Engl. J. Med. *379*, 645–654. https://doi.org/10.1056/NEJMoa1711460.

12. Segal-Maurer, S., DeJesus, E., Stellbrink, H.J., Castagna, A., Richmond, G.J., Sinclair, G.I., Siripassorn, K., Ruane, P.J., Berhe, M., Wang, H., et al. (2022). Capsid Inhibition with Lenacapavir in Multidrug-Resistant HIV-1 Infection. N. Engl. J. Med. *386*, 1793–1803. https://doi.org/10.1056/NEJMoa2115542.

13. Luo, R., Piovoso, M.J., Martinez-Picado, J., and Zurakowski, R. (2011). Optimal antiviral switching to minimize resistance risk in HIV therapy. PLoS One *6*, e27047. https://doi.org/10.1371/journal.pone.0027047.

14. Drechsler, H., and Powderly, W.G. (2002). Switching effective antiretroviral therapy: a review. Clin. Infect. Dis. *35*, 1219–1230. https://doi.org/10.1086/343050.

15. Cahn, P., Madero, J.S., Arribas, J.R., Antinori, A., Ortiz, R., Clarke, A.E., Hung, C.C., Rockstroh, J.K., Girard, P.M., Sievers, J., et al. (2020). Durable Efficacy of Dolutegravir Plus Lamivudine in Antiretroviral Treatment-Naive Adults With HIV-1 Infection: 96-Week Results From the GEMINI-1 and

GEMINI-2 Randomized Clinical Trials. J. Acquir. Immune Defic. Syndr. *83*, 310–318. https://doi.org/10.1097/QAI.0000000000002275.

16. Lombardi, F., Belmonti, S., Quiros-Roldan, E., Latini, A., Castagna, A., D'Ettorre, G., Gagliardini, R., Fabbiani, M., Cauda, R., De Luca, A., et al. (2017). Evolution of blood-associated HIV-1 DNA levels after 48 weeks of switching to atazanavir/ritonavir+lamivudine dual therapy versus continuing triple therapy in the randomized AtLaS-M trial. J. Antimicrob. Chemother. *72*, 2055–2059. https://doi.org/10.1093/jac/dkx068.
17. Fournier, A.L., Hocqueloux, L., Braun, D.L., Metzner, K.J., Kouyos, R.D., Raffi, F., Briant, A.R., Martinez, E., De Lazzari, E., Negredo, E., et al. (2022). Dolutegravir Monotherapy as Maintenance Strategy: A Meta-Analysis of Individual Participant Data From Randomized Controlled Trials. Open Forum Infect. Dis. *9*, ofac107. https://doi.org/10.1093/ofid/ofac107.
18. Braun, D.L., Turk, T., Tschumi, F., Grube, C., Hampel, B., Depmeier, C., Schreiber, P.W., Brugger, S.D., Greiner, M., Steffens, D., et al. (2019). Noninferiority of Simplified Dolutegravir Monotherapy Compared to Continued Combination Antiretroviral Therapy That Was Initiated During Primary Human Immunodeficiency Virus Infection: A Randomized, Controlled, Multisite, Open-label, Noninferiority Trial. Clin. Infect. Dis. *69*, 1489–1497. https://doi.org/10.1093/cid/ciy1131.
19. West, E., Zeeb, M., Grube, C., Kuster, H., Wanner, K., Scheier, T., Neumann, K., Jörimann, L., Hampel, B., Metzner, K.J., et al. (2023). Sustained Viral Suppression With Dolutegravir Monotherapy Over 192 Weeks in Patients Starting Combination Antiretroviral Therapy During Primary Human Immunodeficiency Virus Infection (EARLY-SIMPLIFIED): A Randomized, Controlled, Multi-site, Noninferiority Trial. Clin. Infect. Dis. *77*, 1012–1020. https://doi.org/10.1093/cid/ciad366.
20. Truong, W.R., Schafer, J.J., and Short, W.R. (2015). Once-Daily, Single-Tablet Regimens For the Treatment of HIV-1 Infection. P T. *40*, 44–55.
21. Thoueille, P., Choong, E., Cavassini, M., Buclin, T., and Decosterd, L.A. (2022). Long-acting antiretrovirals: a new era for the management and prevention of HIV infection. J. Antimicrob. Chemother. *77*, 290–302. https://doi.org/10.1093/jac/dkab324.
22. Swindells, S., Andrade-Villanueva, J.F., Richmond, G.J., Rizzardini, G., Baumgarten, A., Masiá, M., Latiff, G., Pokrovsky, V., Bredeek, F., Smith, G., et al. (2020). Long-Acting Cabotegravir and Rilpivirine for Maintenance of HIV-1 Suppression. N. Engl. J. Med. *382*, 1112–1123. https://doi.org/10.1056/NEJMoa1904398.
23. Haynes, B.F., Burton, D.R., and Mascola, J.R. (2019). Multiple roles for HIV broadly neutralizing antibodies. Sci. Transl. Med. *11*, eaaz2686. https://doi.org/10.1126/scitranslmed.aaz2686.
24. Caskey, M. (2020). Broadly neutralizing antibodies for the treatment and prevention of HIV infection. Curr. Opin. HIV AIDS *15*, 49–55. https://doi.org/10.1097/COH.0000000000000600.
25. Stephenson, K.E., and Barouch, D.H. (2016). Broadly Neutralizing Antibodies for HIV Eradication. Curr. HIV AIDS Rep. *13*, 31–37. https://doi.org/10.1007/s11904-016-0299-7.
26. Mylvaganam, G.H., Silvestri, G., and Amara, R.R. (2015). HIV therapeutic vaccines: moving towards a functional cure. Curr. Opin. Immunol. *35*, 1–8. https://doi.org/10.1016/j.coi.2015.05.001.
27. Van de Perre, P., Goga, A., Ngandu, N., Nagot, N., Moodley, D., King, R., Molès, J.P., Mosqueira, B., Chirinda, W., Scarlatti, G., et al. (2021). Eliminating postnatal HIV transmission in high incidence areas: need for complementary biomedical interventions. Lancet *397*, 1316–1324. https://doi.org/10.1016/S0140-6736(21)00570-5.
28. Trkola, A., and Moore, P.L. (2024). Vaccinating people living with HIV: a fast track to preventive and therapeutic HIV vaccines. Lancet Infect. Dis. *24*, e252–e255. https://doi.org/10.1016/S1473-3099(23)00481-4.
29. Back, D.J., Khoo, S.H., Gibbons, S.E., and Merry, C. (2001). The role of therapeutic drug monitoring in treatment of HIV infection. Br. J. Clin. Pharmacol. *52*, 89S–96S. https://doi.org/10.1046/j.1365-2125.2001.0520s1089.x.
30. Dosenovic, P., Pettersson, A.K., Wall, A., Thientosapol, E.S., Feng, J., Weidle, C., Bhullar, K., Kara, E.E., Hartweger, H., Pai, J.A., et al. (2019). Anti-idiotypic antibodies elicit anti-HIV-1-specific B cell responses. J. Exp. Med. *216*, 2316–2330. https://doi.org/10.1084/jem.20190446.
31. Montefiori, D.C. (2005). Evaluating neutralizing antibodies against HIV, SIV, and SHIV in luciferase reporter gene assays. Curr Protoc Immunol *Chapter*. Curr. Protoc. Immunol. *Chapter 12*, 12.11.1–12.11.17. https://doi.org/10.1002/0471142735.im1211s64.
32. Sarzotti-Kelsoe, M., Bailer, R.T., Turk, E., Lin, C.L., Bilska, M., Greene, K.M., Gao, H., Todd, C.A., Ozaki, D.A., Seaman, M.S., et al. (2014). Optimization and validation of the TZM-bl assay for standardized assessments of neutralizing antibodies against HIV-1. J. Immunol. Methods *409*, 131–146. https://doi.org/10.1016/j.jim.2013.11.022.
33. Mahomed, S., Garrett, N., Capparelli, E.V., Osman, F., Mkhize, N.N., Harkoo, I., Gengiah, T.N., Mansoor, L.E., Baxter, C., Archary, D., et al. (2023). Safety and pharmacokinetics of escalating doses of neutralising monoclonal antibody CAP256V2LS administered with and without VRC07-523LS in HIV-negative women in South Africa (CAPRISA 012B): a phase 1, dose-escalation, randomised controlled trial. Lancet. HIV *10*, e230–e243. https://doi.org/10.1016/S2352-3018(23)00003-6.
34. Caskey, M., Schoofs, T., Gruell, H., Settler, A., Karagounis, T., Kreider, E.F., Murrell, B., Pfeifer, N., Nogueira, L., Oliveira, T.Y., et al. (2017). Antibody 10-1074 suppresses viremia in HIV-1-infected individuals. Nat. Med. *23*, 185–191. https://doi.org/10.1038/nm.4268.
35. Gaebler, C., Nogueira, L., Stoffel, E., Oliveira, T.Y., Breton, G., Millard, K.G., Turroja, M., Butler, A., Ramos, V., Seaman, M.S., et al. (2022). Prolonged viral suppression with anti-HIV-1 antibody therapy. Nature *606*, 368–374. https://doi.org/10.1038/s41586-022-04597-1.
36. Pugach, P., Marozsan, A.J., Ketas, T.J., Landes, E.L., Moore, J.P., and Kuhmann, S.E. (2007). HIV-1 clones resistant to a small molecule CCR5 inhibitor use the inhibitor-bound form of CCR5 for entry. Virology *361*, 212–228. https://doi.org/10.1016/j.virol.2006.11.004.
37. Wensing, A., Calvez, V., Ceccherini-Silberstein, F., Charpentier, C., Günthard, H., Paredes, R., Shafer, R., and Richman, D. (2023). 2022 update of the drug resistance mutations in HIV-1. Top Antivir Med *30*, 559–574.
38. Martinez-Picado, J., and Martínez, M.A. (2008). HIV-1 reverse transcriptase inhibitor resistance mutations and fitness: a view from the clinic and ex vivo. Virus Res. *134*, 104–123. https://doi.org/10.1016/j.virusres.2007.12.021.
39. Weber, J., Chakraborty, B., Weberova, J., Miller, M.D., and Quiñones-Mateu, M.E. (2005). Diminished replicative fitness of primary human immunodeficiency virus type 1 isolates harboring the K65R mutation. J. Clin. Microbiol. *43*, 1395–1400. https://doi.org/10.1128/JCM.43.3.1395-1400.2005.
40. Turner, D., Brenner, B., and Wainberg, M.A. (2003). Multiple effects of the M184V resistance mutation in the reverse transcriptase of human immunodeficiency virus type 1. Clin. Diagn. Lab. Immunol. *10*, 979–981. https://doi.org/10.1128/cdli.10.6.979-981.2003.
41. Abram, M.E., Hluhanich, R.M., Goodman, D.D., Andreatta, K.N., Margot, N.A., Ye, L., Niedziela-Majka, A., Barnes, T.L., Novikov, N., Chen, X., et al. (2013). Impact of primary elvitegravir resistance-associated mutations in HIV-1 integrase on drug susceptibility and viral replication fitness. Antimicrob. Agents Chemother. *57*, 2654–2663. https://doi.org/10.1128/AAC.02568-12.
42. Gu, Z., Gao, Q., Fang, H., Salomon, H., Parniak, M.A., Goldberg, E., Cameron, J., and Wainberg, M.A. (1994). Identification of a mutation at codon 65 in the IKKK motif of reverse transcriptase that encodes human immunodeficiency virus resistance to 2',3'-dideoxycytidine and 2',3'-dideoxy-3'-thiacytidine. Antimicrob. Agents Chemother. *38*, 275–281. https://doi.org/10.1128/AAC.38.2.275.
43. Brenner, B.G., and Coutsinos, D. (2009). The K65R mutation in HIV-1 reverse transcriptase: genetic barriers, resistance profile and clinical implications. HIV Ther. *3*, 583–594. https://doi.org/10.2217/hiv.09.40.

CellPress
OPEN ACCESS

44. Wainberg, M.A. (2004). The impact of the M184V substitution on drug resistance and viral fitness. Expert Rev. Anti Infect. Ther. *2*, 147–151. https://doi.org/10.1586/14787210.2.1.147.

45. Basson, A.E., Rhee, S.Y., Parry, C.M., El-Khatib, Z., Charalambous, S., De Oliveira, T., Pillay, D., Hoffmann, C., Katzenstein, D., Shafer, R.W., and Morris, L. (2015). Impact of drug resistance-associated amino acid changes in HIV-1 subtype C on susceptibility to newer nonnucleoside reverse transcriptase inhibitors. Antimicrob. Agents Chemother. *59*, 960–971. https://doi.org/10.1128/AAC.04215-14.

46. Quashie, P.K., Mesplède, T., and Wainberg, M.A. (2013). Evolution of HIV integrase resistance mutations. Curr. Opin. Infect. Dis. *26*, 43–49. https://doi.org/10.1097/QCO.0b013e32835ba81c.

47. Metifiot, M., Marchand, C., Maddali, K., and Pommier, Y. (2010). Resistance to integrase inhibitors. Viruses *2*, 1347–1366. https://doi.org/10.3390/v2071347.

48. Quashie, P.K., Mesplède, T., Han, Y.S., Oliveira, M., Singhroy, D.N., Fujiwara, T., Underwood, M.R., and Wainberg, M.A. (2012). Characterization of the R263K mutation in HIV-1 integrase that confers low-level resistance to the second-generation integrase strand transfer inhibitor dolutegravir. J. Virol. *86*, 2696–2705. https://doi.org/10.1128/JVI.06591-11.

49. Goethals, O., Vos, A., Van Ginderen, M., Geluykens, P., Smits, V., Schols, D., Hertogs, K., and Clayton, R. (2010). Primary mutations selected in vitro with raltegravir confer large fold changes in susceptibility to first-generation integrase inhibitors, but minor fold changes to inhibitors with second-generation resistance profiles. Virology *402*, 338–346. https://doi.org/10.1016/j.virol.2010.03.034.

50. Goethals, O., Clayton, R., Van Ginderen, M., Vereycken, I., Wagemans, E., Geluykens, P., Dockx, K., Strijbos, R., Smits, V., Vos, A., et al. (2008). Resistance mutations in human immunodeficiency virus type 1 integrase selected with elvitegravir confer reduced susceptibility to a wide range of integrase inhibitors. J. Virol. *82*, 10366–10374. https://doi.org/10.1128/JVI.00470-08.

51. Oliveira, M., Ibanescu, R.I., Anstett, K., Mésplède, T., Routy, J.P., Robbins, M.A., and Brenner, B.G.; Montreal Primary HIV PHI Cohort Study Group (2018). Selective resistance profiles emerging in patient-derived clinical isolates with cabotegravir, bictegravir, dolutegravir, and elvitegravir. Retrovirology *15*, 56. https://doi.org/10.1186/s12977-018-0440-3.

52. Orkin, C., Arasteh, K., Górgolas Hernández-Mora, M., Pokrovsky, V., Overton, E.T., Girard, P.M., Oka, S., Walmsley, S., Bettacchi, C., Brinson, C., et al. (2020). Long-Acting Cabotegravir and Rilpivirine after Oral Induction for HIV-1 Infection. N. Engl. J. Med. *382*, 1124–1135. https://doi.org/10.1056/NEJMoa1909512.

53. Adkins, J.C., and Noble, S. (1998). Efavirenz. Drugs *56*, 1055–1066. https://doi.org/10.2165/00003495-199856060-00014.

54. Maggiolo, F. (2009). Efavirenz: a decade of clinical experience in the treatment of HIV. J. Antimicrob. Chemother. *64*, 910–928. https://doi.org/10.1093/jac/dkp334.

55. Saag, M.S. (2006). Emtricitabine, a new antiretroviral agent with activity against HIV and hepatitis B virus. Clin. Infect. Dis. *42*, 126–131. https://doi.org/10.1086/498348.

56. Bang, L.M., and Scott, L.J. (2003). Emtricitabine: an antiretroviral agent for HIV infection. Drugs *63*, 2413–2426, discussion 2425-2416. https://doi.org/10.2165/00003495-200363220-00003.

57. Kandel, C.E., and Walmsley, S.L. (2015). Dolutegravir - a review of the pharmacology, efficacy, and safety in the treatment of HIV. Drug Des. Devel. Ther. *9*, 3547–3555. https://doi.org/10.2147/DDDT.S84850.

58. Katlama, C., and Murphy, R. (2012). Dolutegravir for the treatment of HIV. Expert Opin. Investig. Drugs *21*, 523–530. https://doi.org/10.1517/13543784.2012.661713.

59. Ghosh, A.K., Dawson, Z.L., and Mitsuya, H. (2007). Darunavir, a conceptually new HIV-1 protease inhibitor for the treatment of drug-resistant HIV. Bioorg. Med. Chem. *15*, 7576–7580. https://doi.org/10.1016/j.bmc.2007.09.010.

60. Deeks, E.D. (2014). Darunavir: a review of its use in the management of HIV-1 infection. Drugs *74*, 99–125. https://doi.org/10.1007/s40265-013-0159-3.

61. Sax, P.E., Pozniak, A., Montes, M.L., Koenig, E., DeJesus, E., Stellbrink, H.-J., Antinori, A., Workowski, K., Slim, J., Reynes, J., et al. (2017). Coformulated bictegravir, emtricitabine, and tenofovir alafenamide versus dolutegravir with emtricitabine and tenofovir alafenamide, for initial treatment of HIV-1 infection (GS-US-380–1490): a randomised, double-blind, multicentre, phase 3, non-inferiority trial. Lancet *390*, 2073–2082.

62. Pretorius, E., Klinker, H., and Rosenkranz, B. (2011). The role of therapeutic drug monitoring in the management of patients with human immunodeficiency virus infection. Ther. Drug Monit. *33*, 265–274.

63. Elliot, E., Amara, A., Jackson, A., Moyle, G., Else, L., Khoo, S., Back, D., Owen, A., and Boffito, M. (2016). Dolutegravir and elvitegravir plasma concentrations following cessation of drug intake. J. Antimicrob. Chemother. *71*, 1031–1036. https://doi.org/10.1093/jac/dkv425.

64. Cattaneo, D., Minisci, D., Cozzi, V., Riva, A., Meraviglia, P., Clementi, E., Galli, M., and Gervasoni, C. (2017). Dolutegravir plasma concentrations according to companion antiretroviral drug: unwanted drug interaction or desirable boosting effect? Antivir. Ther. *22*, 353–356. https://doi.org/10.3851/IMP3119.

65. Rhee, S.Y., Grant, P.M., Tzou, P.L., Barrow, G., Harrigan, P.R., Ioannidis, J.P.A., and Shafer, R.W. (2019). A systematic review of the genetic mechanisms of dolutegravir resistance. J. Antimicrob. Chemother. *74*, 3135–3149. https://doi.org/10.1093/jac/dkz256.

66. Li, Q., Liu, Q., Huang, W., Li, X., and Wang, Y. (2018). Current status on the development of pseudoviruses for enveloped viruses. Rev. Med. Virol. *28*, e1963. https://doi.org/10.1002/rmv.1963.

67. Nie, J., Li, Q., Wu, J., Zhao, C., Hao, H., Liu, H., Zhang, L., Nie, L., Qin, H., Wang, M., et al. (2020). Quantification of SARS-CoV-2 neutralizing antibody by a pseudotyped virus-based assay. Nat. Protoc. *15*, 3699–3715. https://doi.org/10.1038/s41596-020-0394-5.

68. Zimmer, G., Locher, S., Berger Rentsch, M., and Halbherr, S.J. (2014). Pseudotyping of vesicular stomatitis virus with the envelope glycoproteins of highly pathogenic avian influenza viruses. J. Gen. Virol. *95*, 1634–1639. https://doi.org/10.1099/vir.0.065201-0.

69. Fukushi, S., Watanabe, R., and Taguchi, F. (2008). Pseudotyped vesicular stomatitis virus for analysis of virus entry mediated by SARS coronavirus spike proteins. Methods Mol. Biol. *454*, 331–338. https://doi.org/10.1007/978-1-59745-181-9_23.

70. Maruyama, J., Nao, N., Miyamoto, H., Maeda, K., Ogawa, H., Yoshida, R., Igarashi, M., and Takada, A. (2016). Characterization of the glycoproteins of bat-derived influenza viruses. Virology *488*, 43–50. https://doi.org/10.1016/j.virol.2015.11.002.

71. Johnson, J.E., Rodgers, W., and Rose, J.K. (1998). A plasma membrane localization signal in the HIV-1 envelope cytoplasmic domain prevents localization at sites of vesicular stomatitis virus budding and incorporation into VSV virions. Virology *251*, 244–252. https://doi.org/10.1006/viro.1998.9429.

72. Li, M., Yang, C., and Compans, R.W. (2001). Mutations in the cytoplasmic tail of murine leukemia virus envelope protein suppress fusion inhibition by R peptide. J. Virol. *75*, 2337–2344. https://doi.org/10.1128/JVI.75.5.2337-2344.2001.

73. Yang, C., and Compans, R.W. (1997). Analysis of the murine leukemia virus R peptide: delineation of the molecular determinants which are important for its fusion inhibition activity. J. Virol. *71*, 8490–8496. https://doi.org/10.1128/JVI.71.11.8490-8496.1997.

74. Durham, N.D., Yewdall, A.W., Chen, P., Lee, R., Zony, C., Robinson, J.E., and Chen, B.K. (2012). Neutralization resistance of virological synapse-mediated HIV-1 Infection is regulated by the gp41 cytoplasmic tail. J. Virol. *86*, 7484–7495. https://doi.org/10.1128/JVI.00230-12.

75. Chen, J., Kovacs, J.M., Peng, H., Rits-Volloch, S., Lu, J., Park, D., Zablowsky, E., Seaman, M.S., and Chen, B. (2015). Effect of the cytoplasmic domain on antigenic characteristics of HIV-1 envelope glycoprotein. Science *349*, 191–195. https://doi.org/10.1126/science.aaa9804.

76. Samal, S., Das, S., Boliar, S., Qureshi, H., Shrivastava, T., Kumar, N., Goswami, S., Bansal, M., and Chakrabarti, B.K. (2018). Cell surface ectodomain integrity of a subset of functional HIV-1 envelopes is dependent on a conserved hydrophilic domain containing region in their C-terminal tail. Retrovirology *15*, 50. https://doi.org/10.1186/s12977-018-0431-4.

77. Medina-Ramirez, M., Sanchez-Merino, V., Sanchez-Palomino, S., Merino-Mansilla, A., Ferreira, C.B., Perez, I., Gonzalez, N., Alvarez, A., Alcocer-Gonzalez, J.M., Garcia, F., et al. (2011). Broadly cross-neutralizing antibodies in HIV-1 patients with undetectable viremia. J. Virol. *85*, 5804–5813. https://doi.org/10.1128/JVI.02482-10.

78. Lorin, V., Fernandez, I., Masse-Ranson, G., Bouvin-Pley, M., Molinos-Albert, L.M., Planchais, C., Hieu, T., Pehau-Arnaudet, G., Hrebik, D., Girelli-Zubani, G., et al. (2022). Epitope convergence of broadly HIV-1 neutralizing IgA and IgG antibody lineages in a viremic controller. J. Exp. Med. *219*, e20212045. https://doi.org/10.1084/jem.20212045.

79. Andrabi, R., Makhdoomi, M.A., Kumar, R., Bala, M., Parray, H., Gupta, A., Kotnala, A., Thirumurthy, V., and Luthra, K. (2014). Highly efficient neutralization by plasma antibodies from human immunodeficiency virus type-1 infected individuals on antiretroviral drug therapy. J. Clin. Immunol. *34*, 504–513. https://doi.org/10.1007/s10875-014-0010-y.

80. Lalezari, J.P., Henry, K., O'Hearn, M., Montaner, J.S.G., Piliero, P.J., Trottier, B., Walmsley, S., Cohen, C., Kuritzkes, D.R., Eron, J.J., Jr., et al. (2003). Enfuvirtide, an HIV-1 fusion inhibitor, for drug-resistant HIV infection in North and South America. N. Engl. J. Med. *348*, 2175–2185.

81. Boffito, M., Back, D.J., Blaschke, T.F., Rowland, M., Bertz, R.J., Gerber, J.G., and Miller, V. (2003). Protein binding in antiretroviral therapies. AIDS Res. Hum. Retroviruses *19*, 825–835. https://doi.org/10.1089/088922203769232629.

82. Schon, A., del Mar Ingaramo, M., and Freire, E. (2003). The binding of HIV-1 protease inhibitors to human serum proteins. Biophys. Chem. *105*, 221–230. https://doi.org/10.1016/s0301-4622(03)00071-1.

83. Almond, L.M., Hoggard, P.G., Edirisinghe, D., Khoo, S.H., and Back, D.J. (2005). Intracellular and plasma pharmacokinetics of efavirenz in HIV-infected individuals. J. Antimicrob. Chemother. *56*, 738–744. https://doi.org/10.1093/jac/dki308.

84. Hinderling, P.H., and Hartmann, D. (2005). The pH dependency of the binding of drugs to plasma proteins in man. Ther. Drug Monit. *27*, 71–85. https://doi.org/10.1097/00007691-200502000-00014.

85. Freind, M.C., Tallón de Lara, C., Kouyos, R.D., Wimmersberger, D., Kuster, H., Aceto, L., Kovari, H., Flepp, M., Schibli, A., Hampel, B., et al. (2024). Cohort Profile: The Zurich Primary HIV Infection Study. Microorganisms *12*, 302. https://doi.org/10.3390/microorganisms12020302.

86. Rusert, P., Kouyos, R.D., Kadelka, C., Ebner, H., Schanz, M., Huber, M., Braun, D.L., Hozé, N., Scherrer, A., Magnus, C., et al. (2016). Determinants of HIV-1 broadly neutralizing antibody induction. Nat. Med. *22*, 1260–1267. https://doi.org/10.1038/nm.4187.

87. Kadelka, C., Liechti, T., Ebner, H., Schanz, M., Rusert, P., Friedrich, N., Stiegeler, E., Braun, D.L., Huber, M., Scherrer, A.U., et al. (2018). Distinct, IgG1-driven antibody response landscapes demarcate individuals with broadly HIV-1 neutralizing activity. J. Exp. Med. *215*, 1589–1608. https://doi.org/10.1084/jem.20180246.

88. Bar, K.J., Sneller, M.C., Harrison, L.J., Justement, J.S., Overton, E.T., Petrone, M.E., Salantes, D.B., Seamon, C.A., Scheinfeld, B., Kwan, R.W., et al. (2016). Effect of HIV antibody VRC01 on viral rebound after treatment interruption. N. Engl. J. Med. *375*, 2037–2050. https://doi.org/10.1056/NEJMoa1608243.

89. Scheid, J.F., Horwitz, J.A., Bar-On, Y., Kreider, E.F., Lu, C.L., Lorenzi, J.C.C., Feldmann, A., Braunschweig, M., Nogueira, L., Oliveira, T., et al. (2016). HIV-1 antibody 3BNC117 suppresses viral rebound in humans during treatment interruption. Nature *535*, 556–560. https://doi.org/10.1038/nature18929.

90. Sneller, M.C., Blazkova, J., Justement, J.S., Shi, V., Kennedy, B.D., Gittens, K., Tolstenko, J., McCormack, G., Whitehead, E.J., Schneck, R.F., et al. (2022). Combination anti-HIV antibodies provide sustained virological suppression. Nature *606*, 375–381. https://doi.org/10.1038/s41586-022-04797-9.

91. Affranchino, J.L., and González, S.A. (2006). Mutations at the C-terminus of the simian immunodeficiency virus envelope glycoprotein affect gp120-gp41 stability on virions. Virology *347*, 217–225. https://doi.org/10.1016/j.virol.2005.11.032.

92. Edwards, T.G., Wyss, S., Reeves, J.D., Zolla-Pazner, S., Hoxie, J.A., Doms, R.W., and Baribaud, F. (2002). Truncation of the cytoplasmic domain induces exposure of conserved regions in the ectodomain of human immunodeficiency virus type 1 envelope protein. J. Virol. *76*, 2683–2691. https://doi.org/10.1128/jvi.76.6.2683-2691.2002.

93. Abrahamyan, L.G., Mkrtchyan, S.R., Binley, J., Lu, M., Melikyan, G.B., and Cohen, F.S. (2005). The cytoplasmic tail slows the folding of human immunodeficiency virus type 1 Env from a late prebundle configuration into the six-helix bundle. J. Virol. *79*, 106–115. https://doi.org/10.1128/JVI.79.1.106-115.2005.

94. Frey, G., Peng, H., Rits-Volloch, S., Morelli, M., Cheng, Y., and Chen, B. (2008). A fusion-intermediate state of HIV-1 gp41 targeted by broadly neutralizing antibodies. Proc. Natl. Acad. Sci. USA *105*, 3739–3744. https://doi.org/10.1073/pnas.0800255105.

95. Alam, S.M., Morelli, M., Dennison, S.M., Liao, H.X., Zhang, R., Xia, S.M., Rits-Volloch, S., Sun, L., Harrison, S.C., Haynes, B.F., and Chen, B. (2009). Role of HIV membrane in neutralization by two broadly neutralizing antibodies. Proc. Natl. Acad. Sci. USA *106*, 20234–20239. https://doi.org/10.1073/pnas.0908713106.

96. Schommers, P., Kim, D.S., Schlotz, M., Kreer, C., Eggeling, R., Hake, A., Stecher, M., Park, J., Radford, C.E., Dingens, A.S., et al. (2023). Dynamics and durability of HIV-1 neutralization are determined by viral replication. Nat. Med. *29*, 2763–2774. https://doi.org/10.1038/s41591-023-02582-3.

97. Gach, J.S., Achenbach, C.J., Chromikova, V., Berzins, B., Lambert, N., Landucci, G., Forthal, D.N., Katlama, C., Jung, B.H., and Murphy, R.L. (2014). HIV-1 specific antibody titers and neutralization among chronically infected patients on long-term suppressive antiretroviral therapy (ART): a cross-sectional study. PLoS One *9*, e85371. https://doi.org/10.1371/journal.pone.0085371.

98. Koch-Weser, J., and Sellers, E.M. (1976). Binding of drugs to serum albumin (first of two parts). N. Engl. J. Med. *294*, 311–316. https://doi.org/10.1056/NEJM197602052940605.

99. Fournier, T., Medjoubi-N, N., and Porquet, D. (2000). Alpha-1-acid glycoprotein. Biochim. Biophys. Acta *1482*, 157–171. https://doi.org/10.1016/s0167-4838(00)00153-9.

100. Tillement, J.P., Lhoste, F., and Giudicelli, J.F. (1978). Diseases and drug protein binding. Clin. Pharmacokinet. *3*, 144–154. https://doi.org/10.2165/00003088-197803020-00004.

101. Denson, D., Coyle, D., Thompson, G., and Myers, J. (1984). Alpha 1-acid glycoprotein and albumin in human serum bupivacaine binding. Clin. Pharmacol. Ther. *35*, 409–415. https://doi.org/10.1038/clpt.1984.51.

102. Urien, S., Brée, F., Testa, B., and Tillement, J.P. (1993). pH-dependence of warfarin binding to alpha 1-acid glycoprotein (orosomucoid). Biochem. J. *289*, 767–770. https://doi.org/10.1042/bj2890767.

103. Leggio, C., Galantini, L., and Pavel, N.V. (2008). About the albumin structure in solution: cigar Expanded form versus heart Normal shape. Phys. Chem. Chem. Phys. *10*, 6741–6750. https://doi.org/10.1039/b808938h.

104. Leonard, W.J., Jr., Vijai, K.K., and Foster, J.F. (1963). A structural transformation in bovine and human plasma albumins in alkaline solution as revealed by rotatory dispersion studies. J. Biol. Chem. *238*, 1984–1988. https://doi.org/10.1016/S0021-9258(18)67930-X.

CellPress
OPEN ACCESS

105. Wanwimolruk, S., and Birkett, D.J. (1982). The effects of N-B transition of human serum albumin on the specific drug-binding sites. Biochim. Biophys. Acta *709*, 247–255. https://doi.org/10.1016/0167-4838(82)90467-8.

106. Wang, T., Zhang, M., Zhou, H., Cui, D., Xu, X., Sun, C., Dai, Y., and Cheng, J. (2019). Establishment and evaluation of a general dissociation technique for antibodies in circulating immune complexes. Clin. Exp. Med. *19*, 65–75. https://doi.org/10.1007/s10238-018-0523-4.

107. Pokriefka, R.A., Manzor, O., Markowitz, N.P., Saravolatz, L.D., Kvale, P., and Donovan, R.M. (1993). Increased detection of human immunodeficiency virus antigenemia after dissociation of immune complexes at low pH. J. Clin. Microbiol. *31*, 1656–1658. https://doi.org/10.1128/jcm.31.6.1656-1658.1993.

108. Miles, S.A., Balden, E., Magpantay, L., Wei, L., Leiblein, A., Hofheinz, D., Toedter, G., Stiehm, E.R., and Bryson, Y. (1993). Rapid serologic testing with immune-complex-dissociated HIV p24 antigen for early detection of HIV infection in neonates. Southern California Pediatric AIDS Consortium. N. Engl. J. Med. *328*, 297–302. https://doi.org/10.1056/NEJM199302043280501.

109. Swiss HIV Cohort Study; Schoeni-Affolter, F., Ledergerber, B., Rickenbach, M., Rudin, C., Günthard, H.F., Telenti, A., Furrer, H., Yerly, S., Francioli, P., and Francioli, P. (2010). Cohort profile: the Swiss HIV Cohort study. Int. J. Epidemiol. *39*, 1179–1189. https://doi.org/10.1093/ije/dyp321.

110. Huber, M., Metzner, K.J., Geissberger, F.D., Shah, C., Leemann, C., Klimkait, T., Böni, J., Trkola, A., and Zagordi, O. (2017). MinVar: A rapid and versatile tool for HIV-1 drug resistance genotyping by deep sequencing. J. Virol. Methods *240*, 7–13. https://doi.org/10.1016/j.jviromet.2016.11.008.

111. Rhee, S.-Y., Gonzales, M.J., Kantor, R., Betts, B.J., Ravela, J., and Shafer, R.W. (2003). Human immunodeficiency virus reverse transcriptase and protease sequence database. Nucleic Acids Res *31*, 298–303. https://doi.org/10.1093/nar/gkg100.

112. Whitt, M.A. (2010). Generation of VSV pseudotypes using recombinant DeltaG-VSV for studies on virus entry, identification of entry inhibitors, and immune responses to vaccines. J. Virol. Methods *169*, 365–374. https://doi.org/10.1016/j.jviromet.2010.08.006.

113. Bates, D., Mächler, M., Bolker, B., and Walker, S. (2015). Fitting Linear Mixed-Effects Models Using lme4. J. Stat. Software *67*, 1–48. https://doi.org/10.18637/jss.v067.i01.

114. Wickham, H. (2016). ggplot2: Elegant Graphics for Data Analysis.

115. Gu, Z., Eils, R., and Schlesner, M. (2016). Complex heatmaps reveal patterns and correlations in multidimensional genomic data. Bioinformatics *32*, 2847–2849. https://doi.org/10.1093/bioinformatics/btw313.

STAR★METHODS

KEY RESOURCES TABLE

REAGENT or RESOURCE	SOURCE	IDENTIFIER
Antibodies		
Monoclonal antibodies, see Table S2	N/A	N/A
Bacterial and virus strains		
VSVΔG*/rLUC	Kerafast	EH1020-PM
Biological samples		
Human plasma samples	Swiss HIV Cohort Study, Zurich Primary HIV Infection Study	N/A
Chemicals, peptides, and recombinant proteins		
Efavirenz	MedChemExpress	HY-10572
Emtricitabine	MedChemExpress	HY-17427
Dolutegravir	MedChemExpress	HY-13238
Darunavir	MedChemExpress	HY-17040
Dynabeads Protein G for Immunoprecipitation	ThermoFisher Scientific	10003D
Dynabeads Protein A for Immunoprecipitation	ThermoFisher Scientific	10001D
Critical commercial assays		
Zeba Spin Desalting Plates, 40K MWCO	ThermoFisher Scientific	A57767
Bright-Glo Luciferase Assay System	Promega	E2650
CellTiter-Glo Luminescent Cell Viability Assay	Promega	G7570
QuikChange II Site-directed mutagenesis kit	Agilent	200523
QuikChange Lightning multi-site directed mutagenesis kit	Agilent	210513
In-Fusion Snap Assembly Master Mix	Takara Bio Inc.	638949
Experimental models: Cell lines		
HEK 293-T cells	ATCC	CRL-3216, RRID: CVCL_0063
TZM-bl cells	NIH AIDS Reagent Program	ARP-8129, RRID: CVCL_B478
Expi293F cells	Thermo Fisher Scientific Inc.	A14528
Oligonucleotides		
Primers for site-directed mutagenesis, see Table S1	This paper	N/A
Recombinant DNA		
pCMV-rev	NIH AIDS Reagent Program	ARP-1443
pNLluc-AM	Provided by A. Marozsan and J. P. Moore (Pugach P. et al.[36])	N/A
HIV^{pv}-MDR13	This paper	N/A
Env plasmids for pseudotyping, see Table S1	N/A	N/A
Software and algorithms		
GraphPad Prism version 10.1.0	GraphPad Software	N/A
R version 4.1.0	www.r-project.org	N/A
Other		
EnVision 2104 Multilabel Reader	Perkin Elmer	N/A

RESOURCE AVAILABILITY

Lead contact

Further information and requests for resources and reagents should be directed to and will be fulfilled by the lead contact, Alexandra Trkola (trkola.alexandra@virology.uzh.ch).

Materials availability

The HIV^{pv}-MDR13 vector is available through a standard MTA from the lead contact upon request. The MDR13 *pol* gene is accessible in GenBank (GenBank: PQ066382).

Data and code availability

- All data reported in this paper will be shared by the lead contact upon request.
- This paper does not report original code.
- Any additional information required to reanalyze the data reported in this paper is available from the lead contact upon request.

EXPERIMENTAL MODEL AND STUDY PARTICIPANT DETAILS

Clinical specimen

Plasma samples from people with HIV (PWH) analyzed in this study were provided by the biobanks of the Swiss HIV Cohort study (SHCS)[1,109] and the Zurich Primary HIV Infection Study (ZPHI).[85] The SHCS is registered under the Swiss National Science longitudinal platform (http://www.snf.ch/en/funding/programmes/longitudinal-studies/Pages/default.aspx#Currently%20supported%20longitudinal%20studies). The ZPHI is an ongoing, observational, non-randomized, single center cohort founded in 2002 that specifically enrolls patients with documented acute or recent primary HIV-1 infection (clinicaltrials.gov: NCT00537966). The SHCS and the ZPHI were approved by the ethics committees of the participating institutions (Kantonale Ethikkommission Bern, Ethikkommission des Kantons St. Gallen, Comité Departemental d'Éthique des Spécialités Médicales et de Médicine Communautaire et de Premier Recours, Kantonale Ethikkommission Zürich, Repubblica et Cantone Ticino–Comitato Ethico Cantonale, Commission Cantonale d'Éthique de la Recherche sur l'Être Humain, Ethikkommission beider Basel for the SHCS and Kantonale Ethikkommission Zürich for the ZPHI) and written informed consent was obtained from all participants. Plasma drug concentration data for ZPHI plasma probed in Figure 5 and reference values were available from clinical routine measurements conducted by the clinical chemistry laboratory at the University Hospital Zurich.

Cell lines

HEK 293-T cells were obtained from the American Type Culture Collection (ATCC) and TZM-bl reporter cells through the NIH AIDS Reagent Program. Both cell lines were cultured in DMEM containing 10% FCS and antibiotics (Penicillin and Streptomycin), and regularly checked for the absence of mycoplasma. Expi293F cells (Thermo Fisher Scientific Inc., Waltham, USA) were cultured in Expi293 Expression Media (Thermo Fisher Scientific Inc., Waltham, USA).

METHOD DETAILS

Antiretrovirals

Efavirenz, emtricitabine, dolutegravir, and darunavir were obtained from MedChemExpress, Monmouth Junction, USA and working stocks were diluted in PBS. To create the drug mix, 0.25 μM of each, efavirenz (EFV), emtricitabine (FTC), dolutegravir (DTG) and darunavir (DRV) were combined.

Antibodies

A full list of mAb sources is provided in Table S2. mAbs 2G12, 4E10, and 447 were obtained from Polymun Scientific GmbH, Austria. All other mAbs were expressed in Expi293F cells by transient transfection using TransIT-PRO transfection reagent (Mirus Bio LLC, Madison, USA) according to the manufacturer's instructions. Seven days after transfection, supernatants were harvested, centrifuged for 45 min at 2,500 x *g* and sterile filtered. Monoclonal antibodies were purified from supernatants using AmMag Protein A Magnetic Beads (GenScript, Piscataway, USA) according to the manufacturer's instructions and eluted using glycine (0.1 M, pH 2.7). For buffer exchange, samples were loaded on a Slide-A-Lyzer Dialysis Cassette G2 10K MWCO (Thermo Fisher Scientific Inc., Waltham, USA) according to the manufacturer's instructions and incubated for 12 h in the desired buffer (10% (w/v) Maltose, 10 mM Tris, 2 mM Acetic acid, pH 4.0 or PBS, pH 7.0). After repetition of the buffer exchange, antibodies were sterile filtered and stored at 4°C.

Virus envelope proteins

A full list of wildtype Env proteins used for pseudotyping and their corresponding Genebank entry, clade, and neutralization tier information is provided in Table S1. Cytoplasmic tail-deleted (ΔCT) Env mutants[71] and MuLV ΔR[72,73] were generated by site-directed

mutagenesis (QuikChange II Site-directed mutagenesis kit, Agilent, Santa Clara, USA) according to the manufacturer's instructions using primers listed in Table S1.

HIV-1 ART-resistant pseudovector

We routinely use the luciferase reporter HIV vector pNLluc-AM[36] in TZM-bl-based neutralization assays.[86] We refer to this vector in the present study as HIV^{pv}-WT. Drug-resistant vectors HIV^{pv}-MDR1 to 12 (Figure 1A) carrying mutations associated with resistance to NRTIs (K65R, M184V), NNRTIs (K101P, Y181C), and INSTIs (Q148H, R263K, G140R, E138A, S153Y) were generated by cloning the *pol* gene of HXB2 into pUC19 vector (Takara Bio Inc., Japan) using the In-Fusion system (Takara Bio Inc., Japan) according to the manufacturer's instructions. Mutations were introduced by site-directed mutagenesis (QuikChange Lightning multi-site directed mutagenesis kit, Agilent, Santa Clara, USA) according to the manufacturer's instructions. Constructs were digested with ApaI and EcoRI (Thermo Fisher Scientific Inc., Waltham, USA) and ligated into NLluc-AM using T4 ligase (Thermo Fisher Scientific Inc., Waltham, USA). All mutations were confirmed by Sanger sequencing (Microsynth AG, Switzerland).

For the clinical isolate-based pseudovirus vector HIV^{pv}-MDR13, a plasma sample of a participant of the SHCS[1] with chronic subtype B HIV infection (>28 years infected) who experienced several drug failures, drug intolerance, and drug toxicities requiring multiple treatment changes over nearly three decades was used. The treatment history comprised the following drugs: nucleos(t)ide treatments (zidovudine, lamivudine, didanosine, zalcitabine, abacavir, tenofovir disoproxil fumarate, tenofovir alafenamide), protease inhibitors (indinavir, nelfinavir, lopinavir/r, atazanavir/r, darunavir/r), non-nucleoside reverse transcriptase inhibitors (delavirdine, efavirenz, rilpivirine, doravirine) and integrase inhibitors (raltegravir, dolutegravir, elvitegravir/c, bictegravir). At the latest treatment failure under bictegravir and doravirine, extensive drug resistance in the protease, reverse transcriptase, and integrase gene was detected by routine genetic resistance testing done by full-length *pol* sequencing. Resistance mutations were detected based on the MinVar pipeline[110] that uses next-generation sequencing data and information from the Stanford HIV database[111] to detect drug resistance mutations. RNA was extracted from plasma samples using EMAG (bioMérieux SA, France) and reverse transcribed using the PrimeScript OneStep RT-PCR Kit (Takara Bio Inc., Japan) according to the manufacturer's instructions. The *pol* gene was amplified using PrimeScript OneStep RT-PCR Kit (Takara Bio Inc., Japan) and the following primers: GCTACAYTAGAAGAAATGATGACA GCAT (forward), GGGGCTTGTTCCATCTATCCTCT/GGGGCTTGTTCCATCTGTCTTCT (reverse). To further amplify the target gene, a nested PCR was performed using Phusion HotStart II DNA Polymerase (Thermo Fisher Scientific Inc., Waltham, USA) and the following primers: GAAGAAATGATGACAGCATGTCAGGGAGT (forward), CCTACCTTGTTATGTCCTGCTTGATA (reverse). The *pol* gene was cloned into HIV^{pv}-WT using the In-Fusion system (Takara Bio Inc., Japan) according to the manufacturer's instructions. Successful cloning was confirmed by Sanger sequencing.

HIV-based Env-pseudotype viruses

HIV^{pv}-WT and HIV^{pv}-MDR Env pseudoviruses were generated by co-transfection of HEK 293-T cells with plasmids encoding the corresponding Env protein and vector at a ratio of 1:3 using 60 μg polyethyleneimine MAX (Polysciences Inc., Warrington, USA). Fresh DMEM containing 10% FCS was added after 8 h of incubation at 37°C. Viral supernatants were harvested 72 h after transfection and the infectivity of the pseudotyped viruses was assessed on TZM-bl cells by measuring the relative light units (RLU) luminescence evoked by the luciferase reporter. If higher titers were needed, virus concentration using sucrose gradient was performed. For this, virus supernatant was sterile filtered and 5 mL sucrose (32% (w/v) in PBS) was added to 25 mL supernatant. Supernatants were centrifuged for 2 h at 28,000 rpm at 4°C and virus pellet was dissolved in 500 μL PBS for 1 h on ice.

VSV-based Env-pseudotype viruses

To produce VSV-based Env-pseudotyped viruses (VSV^{pv}), 4.5x10^{6} HEK 293-T cells were seeded in Poly-L-Lysine solution (Sigma-Aldrich, St. Louis, USA) treated T-75 flasks. The next day, HEK 293-T cells were transfected with 10 μg of plasmid encoding the corresponding Env protein and 10 μg pCMV-rev (NIH AIDS Reagent Program, Division of AIDS, NIAID, NIH) using Lipofectamine 3000 Transfection Reagent (Thermo Fisher Scientific Inc., Waltham, USA) according to the manufacturer's instructions. In brief, 750 μL Opti-MEM (Thermo Fisher Scientific Inc., Waltham, USA), plasmid DNA, and 40 μL P3000 Reagent were mixed and added to 28 μL Lipofectamine 3000 Reagent in 750 μL Opti-MEM. After 20 min of incubation, the transfection mix was added to HEK 293-T cells and fresh DMEM containing 5% FCS was added after 4 h of incubation at 37°C. 24 h post transfection, cells were washed with PBS and infected with a recombinant VSV devoid of the G protein and encoding firefly luciferase (VSVΔG*/rLUC Kerafast, Boston, USA) at a MOI of 3–5.[112] After 90 min of incubation at 37°C, cells were washed twice with PBS and fresh DMEM containing 5% FCS was added. Viral supernatants were harvested 24 h after infection, centrifuged at 500 x *g* for 10 min and supernatant was stored at −80°C. Infectivity of the VSV^{pv} was assessed on TZM-bl cells by measuring the relative light units (RLU) luminescence evoked by the luciferase reporter.

Neutralization assay

The neutralizing capacity of plasma, mAbs, and ARVs efavirenz, emtricitabine, dolutegravir, and darunavir against a panel of 14 viral strains was evaluated on TZM-bl cells using the indicated type of Env pseudotyped viruses in a 384-well format.[86] In brief, heat-inactivated plasma, mAbs, or ARVs were pre-incubated with the respective virus for 1 h before infection of TZM-bl cells. The input of pseudoviruses was adapted to correspond to 10,000 RLU per well as measured on a Dynex MLX luminescence reader (96-well

plates). If infectivity of pseudoviruses was too low to reach this level, undiluted virus was used. Infectivity measured as relative light units (RLU) luminescence evoked by the luciferase reporter was measured on an EnVision Multilabel Reader (PerkinElmer LAS, Germany). Neutralization activity was calculated as the reduction of infectivity compared to infectivity in the absence of plasma, mAbs, or ARVs. Plasma dilutions, mAb or ARV concentrations causing a 50% reduction in infectivity were calculated by fitting a sigmoid dose-response curve (variable slope) to the data using GraphPad Prism version 10 (GraphPad Software, San Diego, USA). If 50% inhibition was not achieved at the highest inhibitor/plasma concentration, a 'greater than' value was recorded.

Purification of antibodies from plasma

Dynabeads Protein A and Protein G magnetic beads (Thermo Fisher Scientific Inc., Waltham, USA) were resuspended and washed twice with binding buffer (0.1 M sodium phosphate, pH 8.0). In the initial setup, 50 μL of each, Protein A and G beads were incubated with 10 μL of plasma diluted 1:10 in binding buffer at 4°C for 30 min to allow antibody binding. Beads were washed three times with binding buffer and bound antibodies were eluted using 25 μL glycine (0.2 M, pH 2.5). After repetition of the elution step, eluates were neutralized using 10 μL Tris-HCl (1 M, pH 9.0) and the resulting 1:6 dilution of the plasma sample was considered in starting dilutions of downstream experiments. Increased bead concentrations and incubation times were probed as indicated and adjusted in the final protocol to 100 μL of each, Protein A and G beads per 10 μL of plasma diluted 1:10 in binding buffer at 4°C for 24 h. Buffer volumes were adjusted for higher plasma input.

ART-DEX: Dissociation and size EXclusion

Samples were purified using Zeba 96-well Spin Desalting Plates, 40K molecular weight cut-off (MWCO) (Thermo Fisher Scientific Inc., Waltham, USA) according to the manufacturer's instructions. For Figures 4, 5, and 6, plasma samples were purified using Zeba 96-well Spin Desalting Plates (Catalog number: 87774).

Plasma samples were either directly added to the spin plates, following acid treatment alone or after alkaline treatment followed by acid treatment, as indicated. The conditions in the individual treatments and the combined alkaline/acid treatment were identical. The combined alkaline/acid treatment was selected for the ART-DEX protocol. For the alkaline treatment, plasma samples were diluted 1:2.5 in phosphate buffer (0.1 M, pH 10) and incubated for 2 h at room temperature. Zeba 96-well Spin Desalting Plates, 40K MWCO were washed with phosphate buffer (0.1 M, pH 10). Samples were applied to the washed spin plates, centrifuged for 2 min at 1000 x *g*, and the eluted plasma was diluted 1:2 in acetate buffer (0.1 M, pH 3.6). The acidified plasma was then incubated for 1 h at room temperature. Zeba 96-well Spin Desalting Plates, 40K MWCO were washed with acetate buffer (0.1 M, pH 3.6) before a second spin serration of the acidified plasma samples (2 min at 1000 x *g*). The eluted plasma was adjusted to a final dilution of 1:25 in DMEM containing 10% FCS, 25 mM HEPES, and antibiotics.

Zeba 96-well Spin Desalting Plates (Catalog number: 87774) were discontinued close to finalization of our study and replaced by a follow-up product Zeba 96-well Spin Desalting Plates (Catalog number: A57767). To verify that the updated plates perform equally well, we repeated key experiments, namely the MuLV screening data (Figure S4D) and neutralization activity off ART (Figures S5D and S5E) with the updated plates. We adopted the ART-DEX protocol for this slightly according to the manufacturer's instructions and found it to perform equally well as the protocol with the prior version of the plates. The final ART-DEX protocol based on the Zeba 96-well Spin Desalting Plates (Catalog number: A57767) is as follows:

For the alkaline treatment, plasma samples were diluted 1:2.5 in phosphate buffer (0.1 M, pH 10) and incubated for 2 h at room temperature. Zeba 96-well Spin Desalting Plates, 40K MWCO were washed three times with phosphate buffer (0.1 M, pH 10). Samples were applied to the washed spin plates, centrifuged for 3 min at 700 x *g*, and the eluted plasma was diluted 1:2 in acetate buffer (0.1 M, pH 3.6). The acidified plasma was then incubated for 1 h at room temperature. Zeba 96-well Spin Desalting Plates, 40K MWCO were washed three times with acetate buffer (0.1 M, pH 3.6) before a second spin serration of the acidified plasma samples (3 min at 700 x *g*). The eluted plasma was adjusted to a final dilution of 1:25 in DMEM containing 10% FCS, 25 mM HEPES, and antibiotics.

Cell viability assay

Cell viability was assessed using the CellTiter-Glo Luminescent Cell Viability Assay (Promega Corporation, Madison, USA) according to the manufacturer's instructions. In brief, samples were serially diluted on TZM-bl cells in a 384-well format. After 72 h, CellTiter-Glo reagent was added to the wells and plates were shaken for 2 min. After incubation for 10 min at room temperature, light emission was measured on an EnVision Multilabel Reader (PerkinElmer LAS, Germany).

QUANTIFICATION AND STATISTICAL ANALYSIS

Statistical analyses

Statistical analyses were performed in R (Version 4.1.0). Linear mixed-effects models were fitted using the lme4 package in R.[113] Linear models were fitted using the stats package in R. Multiple testing was adjusted using Bonferroni correction for multiple comparisons. Statistical details of each experiment can be found in the corresponding figure legends.

Programs

Figures 1, 2, 3, and 4 were generated with GraphPad Prism version 9 (GraphPad Software, San Diego, USA). Figures 5 and 6 were produced using the ggplot2[114] and ComplexHeatmap[115] packages in R. Figures 3 and 4 were in part created using BioRender.com (Paid subscription, BioRender, Canada). Figures were assembled and finalized in Affinity Designer (Serif Europe Ltd, United Kingdom).

Cell Reports Medicine, Volume *5*

Supplemental information

Decoupling HIV-1 antiretroviral drug inhibition from plasma antibody activity to evaluate broadly neutralizing antibody therapeutics and vaccines

Magdalena Schwarzmüller, Cristina Lozano, Merle Schanz, Irene A. Abela, Silvan Grosse-Holz, Selina Epp, Martina Curcio, Jule Greshake, Peter Rusert, Michael Huber, Roger D. Kouyos, Huldrych F. Günthard, Alexandra Trkola, and the Swiss HIV Cohort Study

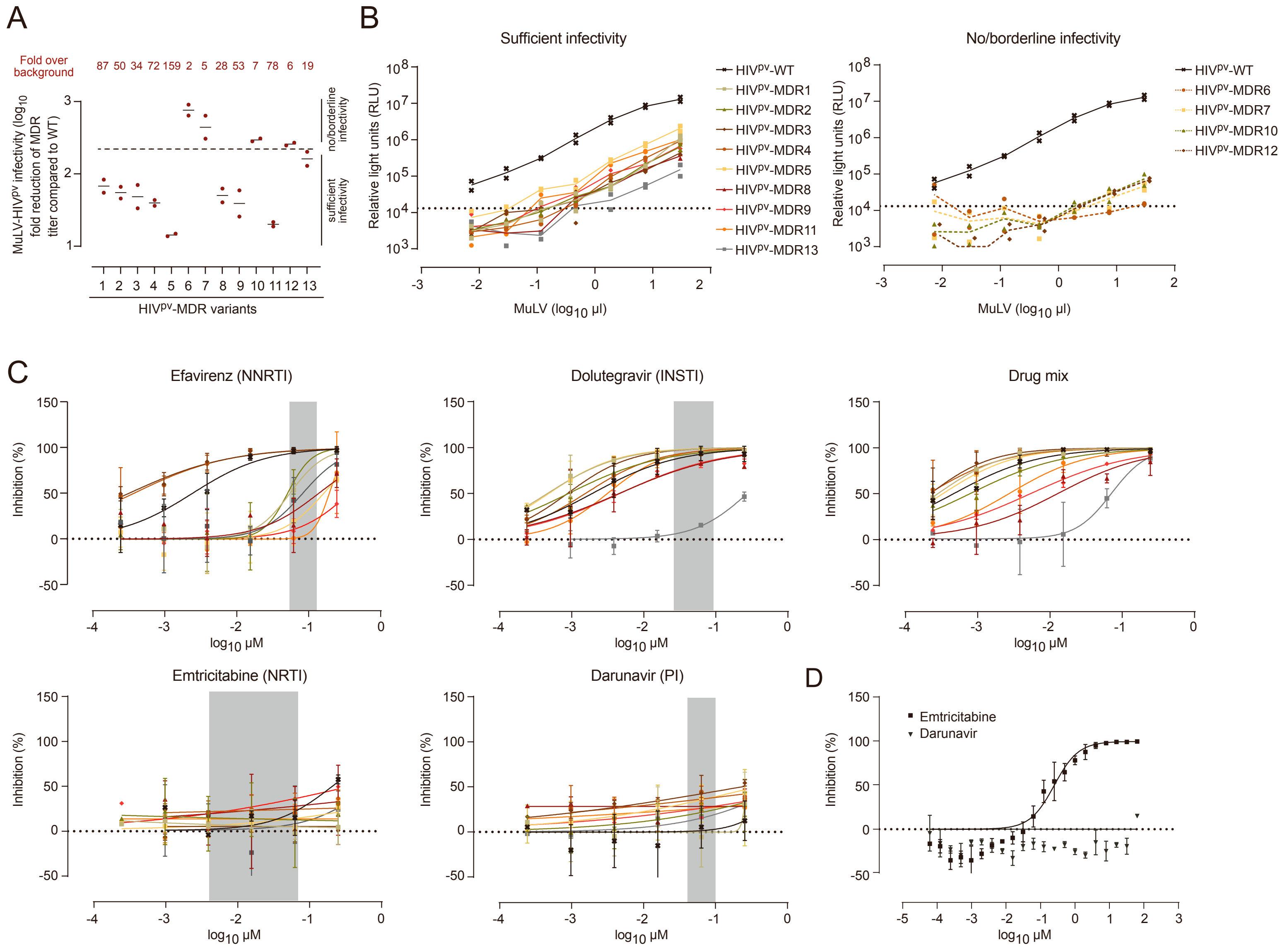

Figure S1. Screening combinations of ART-resistance mutations, related to Figure 1. (A) Infectivity assessment of HIV^{pv}-MDR1-13 pseudotyped with MuLV envelope. Infection of TZM-bl cells with serial dilutions of virus. Infectivity was recorded as relative light units (RLU) luminescence. Fold reduction in infectivity of variants in relation to HIV^{pv}-WT is depicted. Numbers indicate average fold over background values with undiluted virus input. HIV^{pv}-MDR6, 7, 10, and 12 recorded no/borderline infectivity and were not followed further. Data from two independent experiments are shown. (B) Infection of TZM-bl cells with serial dilutions of MuLV-pseudotyped viruses with the indicated resistance mutations. Infectivity was recorded as RLU luminescence and compared to infectivity of HIV^{pv}-WT pseudovirus. Data from two independent experiments are shown. Dotted line indicates mean background signal. Pseudotyped viruses with sufficient infectivity are shown on the left, pseudotyped viruses with no or borderline infectivity are shown on the right. (C) Inhibition of MuLV-pseudotyped viruses with sufficient infectivity by serially diluted antiretroviral drugs was compared to inhibition of HIV^{pv}-WT pseudoviruses on TZM-bl cells. Mean and standard deviation from two independent experiments are shown. Grey boxes indicate drug concentrations expected in plasma of PWH (see Table S4). Drug mix: 25 µM of each, efavirenz (EFV), emtricitabine (FTC), dolutegravir (DTG), and darunavir (DRV) were combined and titrated. (D) Inhibition of MuLV-pseudotyped HIV^{pv}-WT pseudoviruses by serial dilutions of emtricitabine and darunavir. Mean and standard deviation from two independent experiments are shown.

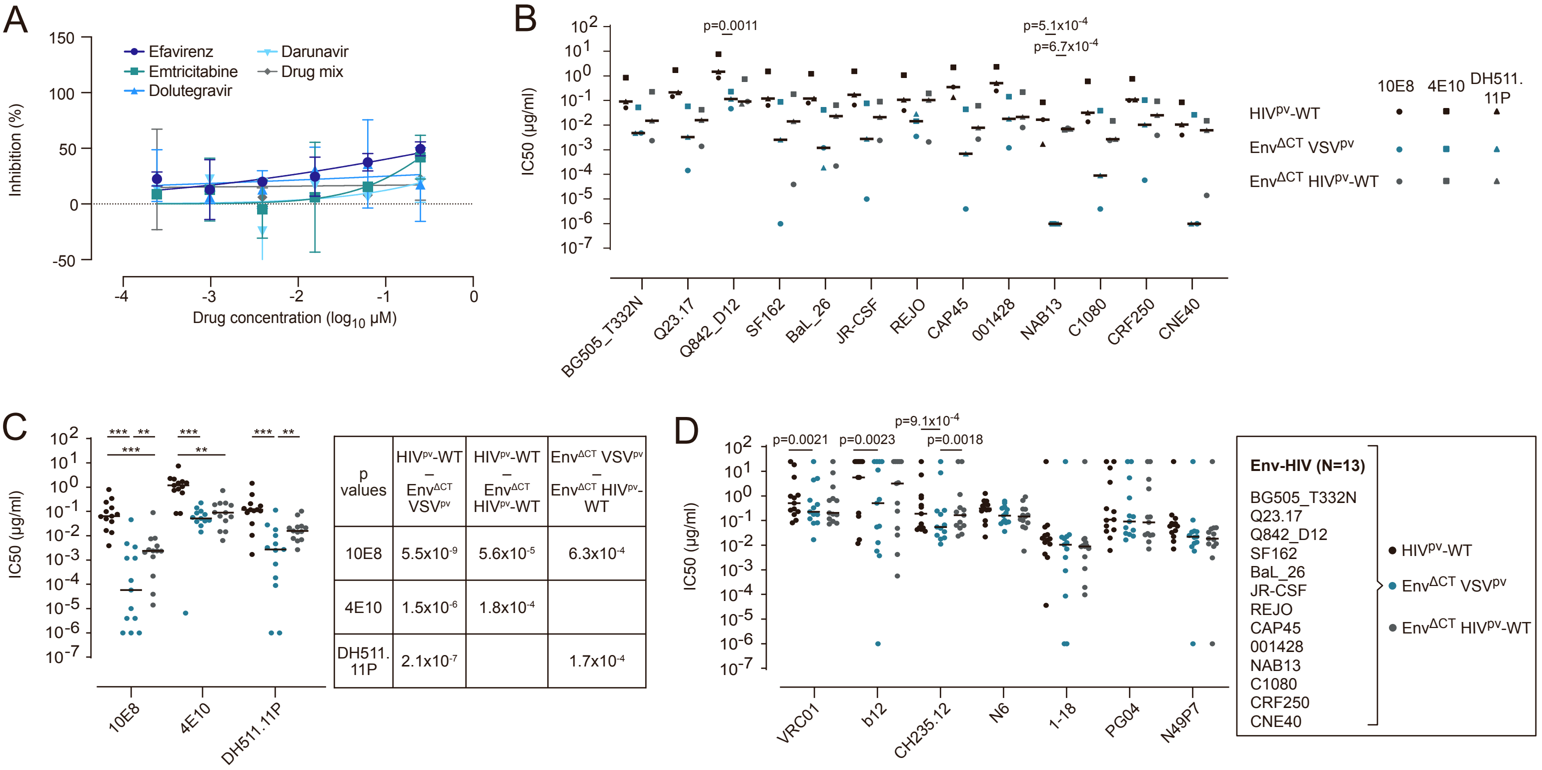

p values	HIV^{pv}-WT – Env$^{\Delta CT}$ VSV^{pv}	HIV^{pv}-WT – Env$^{\Delta CT}$ HIV^{pv}-WT	Env$^{\Delta CT}$ VSV^{pv} – Env$^{\Delta CT}$ HIV^{pv}-WT
10E8	5.5×10^{-9}	5.6×10^{-5}	6.3×10^{-4}
4E10	1.5×10^{-6}	1.8×10^{-4}	
DH511.11P	2.1×10^{-7}		1.7×10^{-4}

Figure S2. Complete ARV resistance but partially increased sensitivity to Env-directed mAbs of VSV-based pseudoviruses, related to Figure 2. (A) Inhibition of TZM-bl cell infection of MuLV-pseudotyped VSV^{pv} viruses by serially diluted antiretroviral drugs. Inhibition was assessed as the reduction of infectivity compared to untreated control. Mean and standard deviation of two independent experiments are shown. Drug mix: 25 µM of each, efavirenz, emtricitabine, dolutegravir, and darunavir were combined and titrated. (B)-(C) Comparison of MPER bnAbs for neutralization of 13 Env viruses in the context of HIV^{pv}-WT (black), Env$^{\Delta CT}$ VSV^{pv} (blue), and Env$^{\Delta CT}$ HIV^{pv}-WT (grey) pseudoviruses. Mean IC50 values of two independent experiments are shown. (B) Data depicted per individual Env virus. Significance thresholds between backbones are adjusted on multiple testing using Bonferroni correction and indicated as followed: * $p<0.05/39$, ** $p<0.01/39$, *** $p<0.001/39$. (C) Data are depicted per method across 13 Env virus panel. Significance thresholds between backbones are adjusted on multiple testing using Bonferroni correction and indicated as followed: * $p<0.05/9$, ** $p<0.01/9$, *** $p<0.001/9$. (D) Neutralization of the 13 virus panel by 7 CD4bs-directed mAbs as in (C). Data are depicted per method across 13 Env virus panel. Significance thresholds between backbones are adjusted on multiple testing using Bonferroni correction and indicated as followed: * $p<0.05/21$, ** $p<0.01/21$, *** $p<0.001/21$.

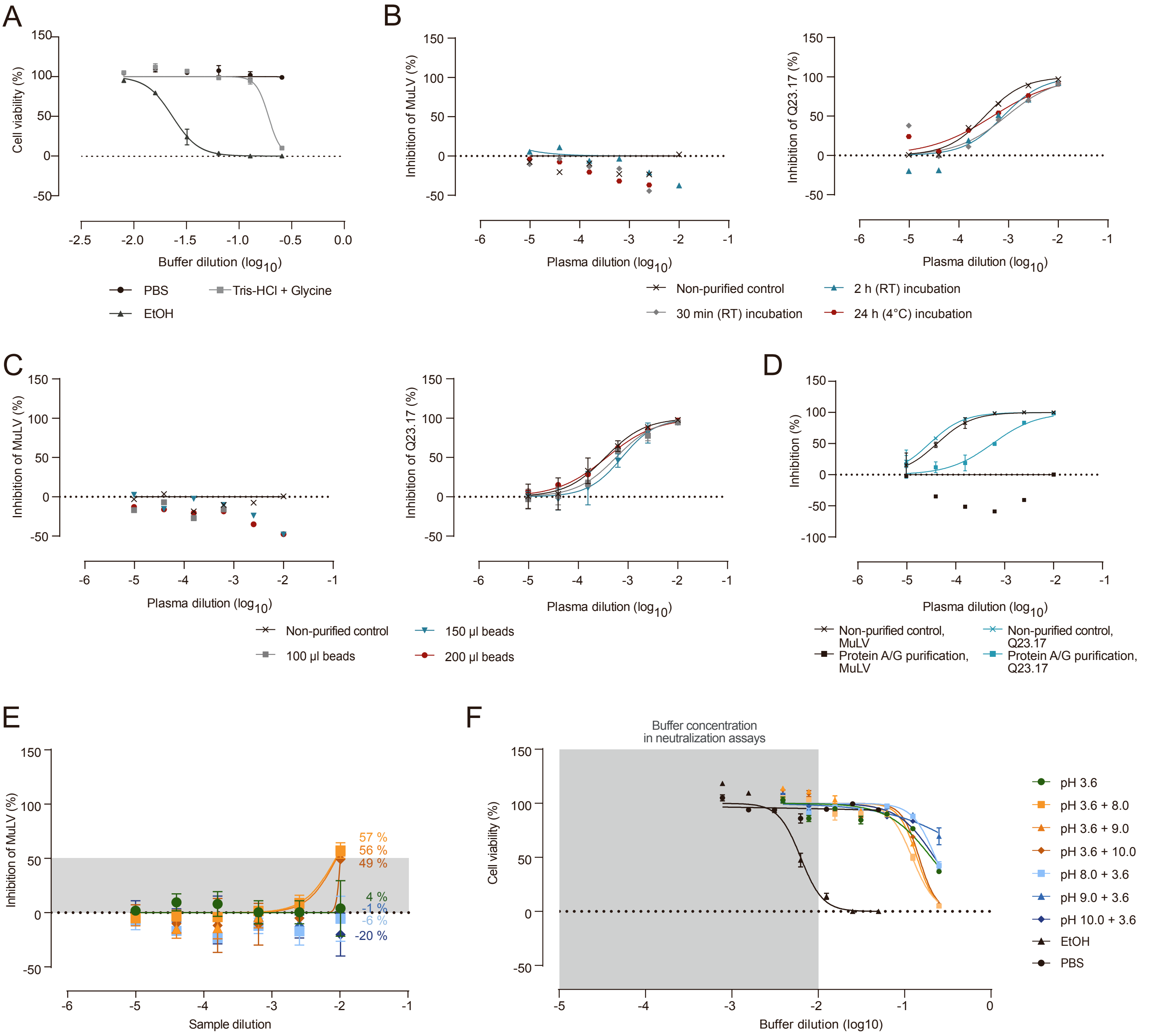

Figure S3. Strategies to separate ARVs from plasma antibodies, related to Figure 3 and 4. (A) Cytotoxicity of the bead elution buffer (100 µl Glycine (0.2 M, pH 2.5) and 20 µl Tris-HCl (1 M, pH 9.0)) compared to treatment with PBS or Ethanol (EtOH) was analyzed on TZM-bl cells. Cell viability was assessed in duplicates and normalized to untreated cells. (B)-(C): Data related to Figure 3. (B) VRC01 (250 µg/ml) spiked into 10 µl healthy donor plasma was added to 100 µl Protein A and G magnetic beads and incubated for the indicated time before antibodies were eluted from the magnetic beads. Inhibition of MuLV (left) and Q23.17 (right) by purified antibody preparations was analyzed in an HIV^{pv}-WT neutralization assay. (C) VRC01 (250 µg/ml) spiked into 10 µl healthy donor plasma was added to indicated amounts of Protein A and G magnetic beads and incubated for 24 h at 4°C before antibodies were eluted from the magnetic beads. Inhibition of MuLV (left) and Q23.17 (right) by purified antibodies was analyzed in an HIV^{pv}-WT neutralization assay. (D) VRC01 (500 µg/ml) and ARVs (25 µM) spiked into 10 µl healthy donor plasma were added to 200 µl Protein A and G magnetic beads and incubated for 24 h at 4°C before antibodies were eluted from the magnetic beads. Inhibition of MuLV and Q23.17 was analyzed in an HIV^{pv}-WT neutralization assay. (E) TZM-bl culture media (without ARVs, mAbs, or plasma) was subjected to the indicated pH and was then used directly in an inhibition assay using MuLV (HIV^{pv}-WT) on TZM-bl cells without prior size-exclusion step. Conditions with acid pH treatment (1 h) are depicted in green, acid pH treatment (2 h) followed by alkaline pH (1 h) in yellow and alkaline pH (2 h) followed by acid pH (1 h) in blue. Percent inhibition at the lowest dilution (= highest concentration) is indicated. Data from two independent experiments are shown. (F) Cytotoxicity of the buffers used for ART-DEX compared to treatment with PBS or Ethanol (EtOH) was analyzed on TZM-bl cells. Cell viability was assessed in duplicates and normalized to untreated cells. Conditions with acid pH treatment (1 h) are depicted in green, acid pH treatment (2 h) followed by alkaline pH (1 h) in yellow and alkaline pH (2 h) followed by acid pH (1 h) in blue. The concentration range of ART-DEX buffers as used in the protocol is indicated with a grey shaded box.

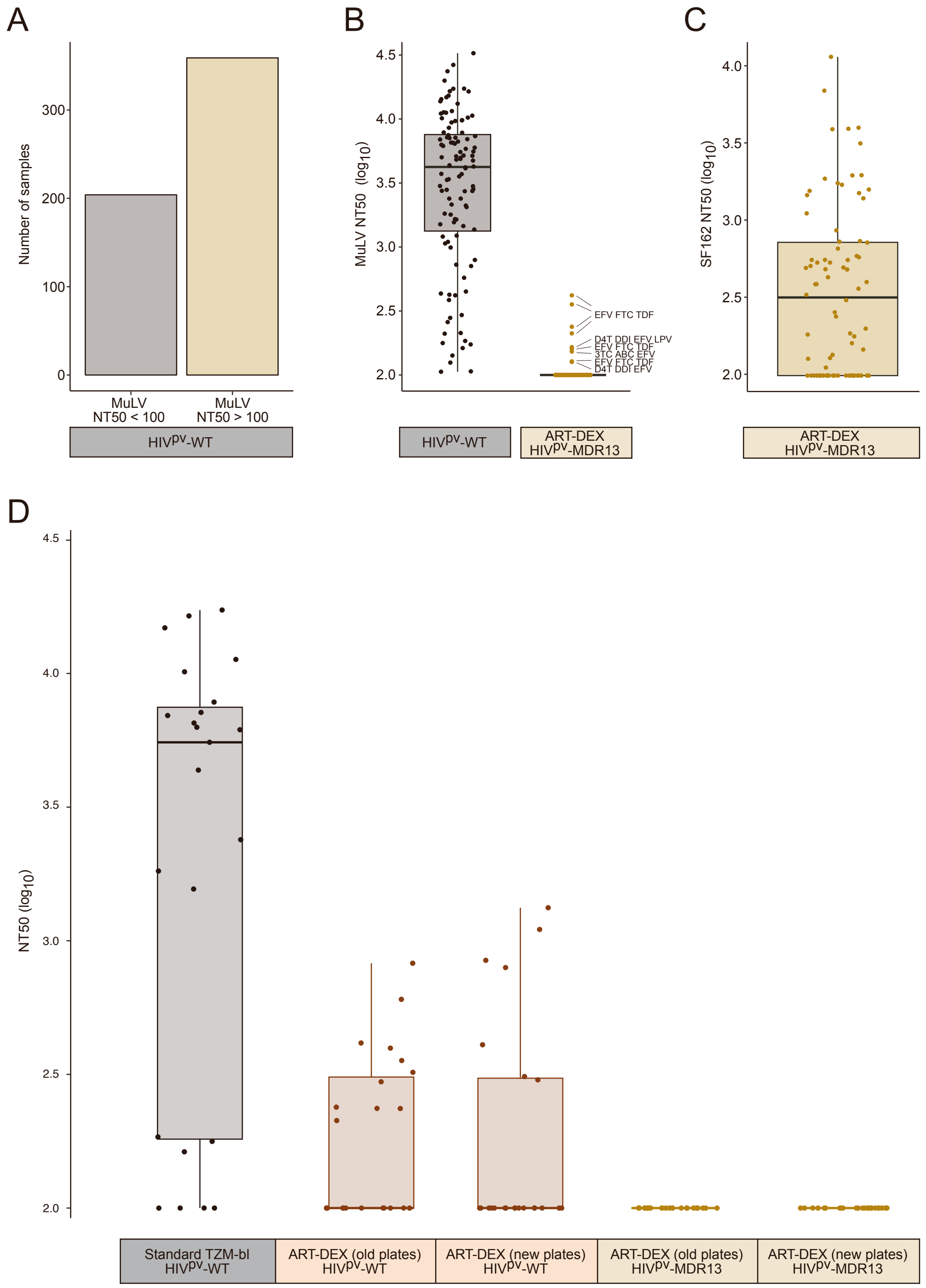

Figure S4. Verifying the capacity of ARV removal strategies in plasma of ART-treated PWH, related to Figure 5. (A) Plasma samples (N=563) from PWH with different ART combination regimen were tested for inhibitory activity against MuLV using the standard TZM-bl assay. Number of samples with (NT50>100) and without (NT50<100) MuLV inhibition are shown. (B) 108 plasma samples with MuLV inhibition in (A) were tested for the inhibitory activity against MuLV either using the standard TZM-bl assay or ART-DEX in combination with HIV^{pv}-MDR13. NT50 titers against MuLV are shown. Drug combinations with residual inhibitory activity are indicated. (C) 80 plasma samples without MuLV inhibition after ART-DEX/HIV^{pv}-MDR13 were tested for their neutralization activity against SF162. NT50 titers against SF162 are shown. (D) Zeba 96-well Spin Desalting Plates (Catalogue number: 87774) were discontinued close to finalization of our study and replaced by a follow-up product Zeba 96-well Spin Desalting Plates (Catalogue number: A57767). To verify that the new plates perform equally well we conducted a control experiment: Plasma samples from PWH (N = 23) with different ART combination regimen were tested for MuLV inhibition using the standard TZM-bl assay (untreated control) or ART-DEX alone or in combination with HIV^{pv}-MDR13. MuLV inhibition after ART-DEX was compared between old and updated spin plates. NT50 titers from two independent experiments are shown. Boxplots represent median with the middle line, upper and lower quartiles with the box limits, and 1.5 x interquartile ranges with the whiskers.

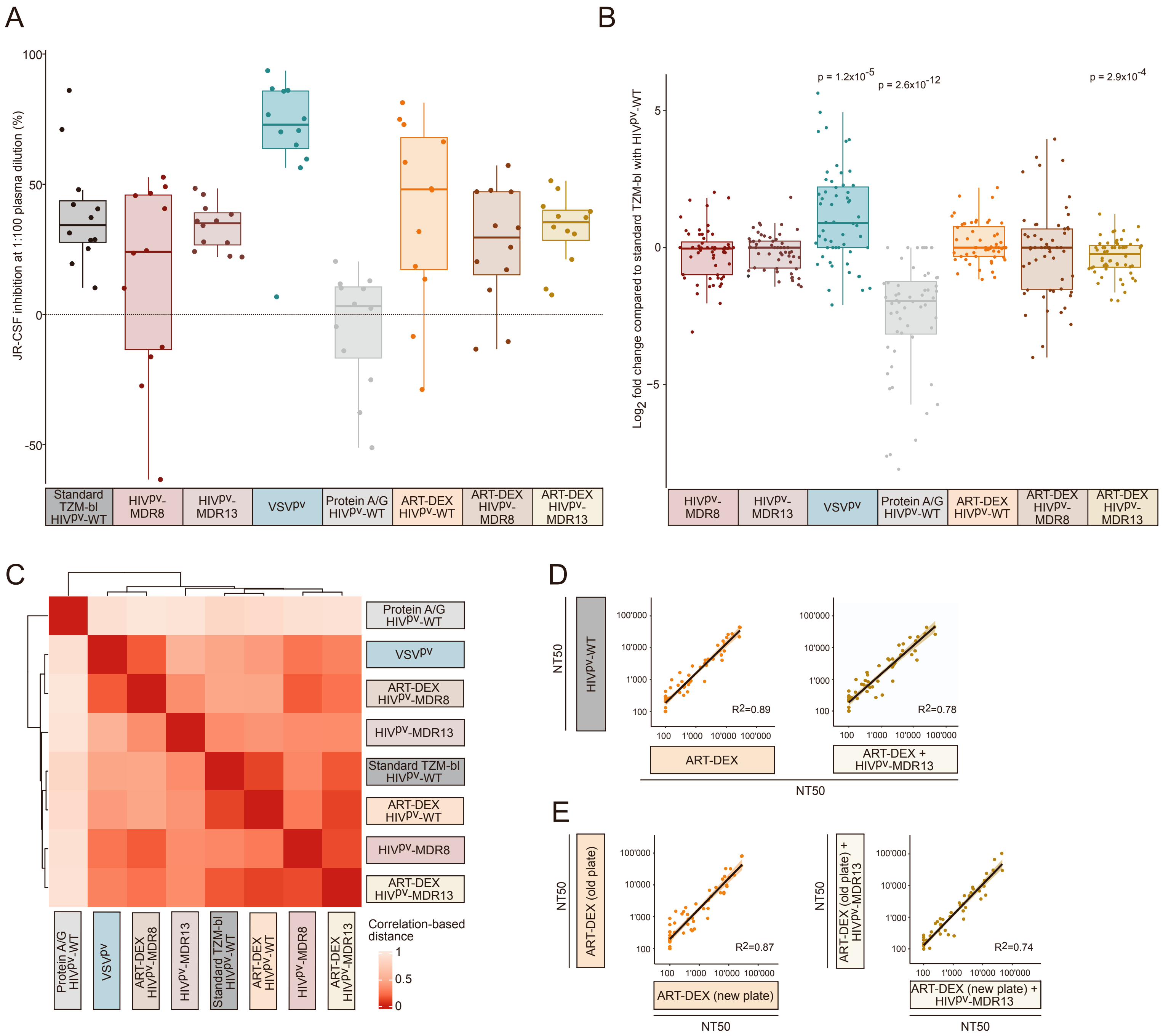

Figure S5. Assessing the accuracy of ART-free neutralization methods in monitoring *in vivo* neutralization capacity, related to Figure 6. Plasma samples of bnAb inducers S5206-G5 and S51517 before the initiation of ART were analyzed for their neutralization activity. (A) Plasma neutralization activity of bnAb inducer S5206-G5 against JR-CSF was compared across all probed ART-free methods. Percent inhibition of JR-CSF at the 1:100 plasma dilution from two independent experiments is shown. Boxplots represent median with the middle line, upper and lower quartiles with the box limits, and 1.5 x interquartile ranges with the whiskers. (B) Fold changes of NT50 titers compared to the standard TZM-bl assay with HIV^{pv}-WT are shown. Significance in variation was tested using one-sided t-test and significance thresholds are adjusted for multiple testing using Bonferroni correction and indicated as followed: * p<0.05/7, ** p<0.01/7, *** p<0.001/7. (C) Heatmap showing the correlation-based distance between the different ART-free neutralization methods and the standard TZM-bl-based neutralization assay. (D) Correlation analysis of NT50 titers based on HIV^{pv}-WT and the ART-DEX (performed with newly released 96-well Spin Desalting Plates (Catalogue number: A57767)) alone or in combination with HIV^{pv}-MDR13. (E) Correlation analysis of NT50 titers based on prior version of 96-well spin plates (Catalogue number: 87774) and updated 96-well spin plates (Catalogue number: A57767) either with ART-DEX alone or in combination with HIV^{pv}-MDR13 confirms equal performance of both plate versions.

Table S1: Virus screening panel, related to Figure 1, 2, 6, and STAR Methods. All viruses were generated as HIV^{pv}-WT, HIV^{pv}-MDR8, HIV^{pv}-MDR13, and VSV^{pv} pseudoviruses with full-length Env or Env$^{\Delta CT}$. Virus titers (RLU/µl) for HIV^{pv}-MDR8 and HIV^{pv}-MDR13 as well as average fold over background with undiluted virus input are listed.

**Virus (full name)**	**Virus (name used in study)**	**Clade**	**Neutralization tier**	**Genebank entry code**	**Infectivity (RLU/µl) with MDR8 (Fold over background)**	**Infectivity (RLU/µl) with MDR13 (Fold over background)**	**Forward primer ΔCT/ΔR**
BG505_ W6M_ENV_ A5_T332N	BG505_ T332N	A	2	DQ208456	11113 (11)	20115 (32)	GTAATACATAGAGTTAGG TAGGGATACTCACCTTTG TCG
Q23.17	Q23.17	A	1B	AF004885	132862 (106)	155205 (153)	CTGTAATAAATAGAGTTA GGTAGGGATACTCACCT TTGTC
Q842_d12_ PNS70d	Q842_D 12	A	2	AF407160	22289 (18)	32105 (35)	CTGTAATAAATAGAGTTA GGTAGGGATACTCACCT TTGTC
SF162	SF162	B	1A	EU123924	36338 (50)	67276 (54)	GTGAATAGAGTTAGGTA GGGATACTCACCATTATC
BaL_26	BaL_26	B	1B	DQ318211	110905 (109)	92817 (142)	GTGAATAGAGTTAGGTA GGGATACTCACCATTATC
JR-CSF	JR-CSF	B	2	AY669726	54638 (56)	169097 (166)	GTGAATAGAGTTAGGTA GGGATACTCACCCTTATC
REJO4541 clone 67	REJO	B	2	AY835449	73298 (79)	151816 (130)	CTATAATAAATAGAGTTA GGTAGGGATACTCACCA TTATCG
CAP45_2_ 00_G3	CAP45	C	2	DQ435682	15564 (9)	30374 (43)	GTGAAAAGAGTTAGGTA GGGATACTCACCTTTGTC
HIV_001428 _2_42	001428	C	2	EF117266	72372 (86)	147854 (170)	GTGAATAGAGTTAGGTA GGGATACTCACCTTTGTC
NAB13pre_ cl_9	NAB13	G	2	EU023937	95684 (98)	111330 (127)	CTATAATAAATAGAGTTA GGTAGGGATACTCACCC TTGTC
C1080_c03	C1080	AE	2	JN944660	43610 (55)	68127 (70)	GTAAATAGAGTTAGGTA GGGATACTCACCTTTGTC
CRF02_ cl_250	CRF250	AG	2	EU513189	49107 (46)	66399 (95)	GTAATAAATAGAGTTAGG TAGGGATACTCACCTTTG TC
CNE40	CNE40	BC	1B	HM215414	27660 (25)	21322 (34)	GTAAATAGAGTTAGGTA GGGATACTCACCTTTGTC
MuLV	MuLV				12212 (10)	57963 (19)	CAGTGGTCCAGGCTCTA TGATTGACTCAACAATAT CACC

Table S2: List of monoclonal antibodies used in this study, related to Figure 2.

Monoclonal antibody	Epitope region	Reference
1-18	CD4bs	Schommers 2020 [S1]
B6	CD4bs	Burton 1991 [S2]
b12	CD4bs	Burton 1991 [S2]
CH235.12	CD4bs	Bonsignori 2016 [S3]
N49P7	CD4bs	Sajadi 2018 [S4]
N6	CD4bs	Huang 2016 [S5]
VRC01	CD4bs	Wu 2010 [S6]
PG04	CD4bs	Wu 2011 [S7]
17b	CD4i	Thali 1993 [S8]
48d	CD4i	Thali 1993 [S8]
CH01	V2 glycan	Bonsignori 2011 [S9]
PG16	V2 glycan	Walker 2009 [S10]
PGDM1400	V2 glycan	Sok 2014 [S11]
PGT145	V2 glycan	Walker 2011 [S12]
VRC26.25	V2 glycan	Doria-Rose 2016 [S13]
1-79	V3 glycan	Scheid 2009 [S14]
2G12	V3 glycan	Buchacher 1994 [S15]
447	V3 glycan	Buchbinder 1992 [S16]
PGDM12	V3 glycan	Sok 2016 [S17]
PGDM21	V3 glycan	Sok 2016 [S17]
PGT121	V3 glycan	Walker 2011 [S12]
PGT128	V3 glycan	Walker 2011 [S12]
ACS202	Fusion peptide/ gp41-gp120 interface	Van Gils 2016 [S18]
PGT151	Fusion peptide/ gp41-gp120 interface	Falkowska 2014 [S19]
VRC34.01	Fusion peptide/ gp41-gp120 interface	Kong 2016 [S20]
SF12	Silent face	Schoofs 2019 [S21]
4E10	MPER	Buchacher 1994 [S15]
10E8	MPER	Huang 2012 [S22]
DH511.11P	MPER	Williams 2017 [S23]
Z13e1	MPER	Nelson 2007 [S24]

Table S3: High sensitivity of VSV-based pseudoviruses against certain mAbs, related to Figure 2. Neutralization of a 13 virus panel and MuLV as control by the indicated mAbs was compared between HIV^{pv}-WT, Env$^{\Delta CT}$ VSV^{pv} and Env$^{\Delta CT}$ HIV^{pv}-WT pseudoviruses. Mean IC50 values of two independent experiments are listed.

		IC50 (µg/ml)																													
		bnAbs																								Low/non-neutralizing mAbs					
		CD4bs							V1V2							V3			Fusion peptide/interface			Silent face	MPER			CD4bs	V3		CD4i		MPER
Backbone	Env	VRC01	b12	CH235.12	N6	1-18	PG04	N49P7	PG16	PGDM1400	PGT145	CH01	VRC26.25	PGT121	PGT128	2G12	PGDM12	PGDM21	PGT151	VRC34.01	ACS202	SF12	10E8	4E10	DH511.11P	B6	1-79	447	48d	17b	Z13e1
HIV^{pv}-WT	MuLV	25.0	25.0	25.0	25.0	25.0	25.0	25.0	25.0	25.0	25.0	25.0	25.0	25.0	25.0	25.0	25.0	25.0	25.0	25.0	25.0	25.0	25.0	25.0	25.0	25.0	25.0	25.0	25.0	25.0	25.0
	Bal26	0.17	0.02	0.04	0.25	0.00	0.30	0.03	9.44	6.05	5.66	25.0	25.0	0.03	0.04	0.72	0.04	0.13	0.04	7.99	25.0	0.18	0.08	1.19	0.12	21.5	0.15	0.04	25.0	10.9	25.0
	BG505_T332N	0.27	25.0	0.05	0.39	0.02	0.04	0.08	0.01	0.06	0.06	0.37	0.00	0.03	0.01	0.95	0.03	0.13	0.00	0.14	25.0	0.02	0.05	0.83	0.09	25.0	25.0	25.0	25.0	25.0	16.7
	C1080	5.78	5.65	1.03	1.26	0.07	3.61	0.17	0.00	0.01	0.06	1.76	0.16	25.0	0.24	25.0	19.2	25.0	13.8	3.51	7.82	0.03	0.01	0.59	0.03	25.0	25.0	25.0	20.4	25.0	7.52
	CAP45	18.6	0.20	0.35	0.49	25.0	13.7	0.12	0.00	0.01	0.00	0.06	0.00	5.12	25.0	25.0	25.0	25.0	0.00	0.09	25.0	25.0	0.34	2.15	0.13	25.0	25.0	25.0	2.07	25.0	25.0
	CNE40	1.07	5.64	0.07	0.31	0.03	0.23	0.07	25.0	6.71	0.73	25.0	0.60	0.94	25.0	25.0	23.6	25.0	2.76	0.17	25.0	25.0	0.00	0.08	0.01	0.17	0.33	25.0	25.0	0.08	25.0
	CRF250	25.0	25.0	25.0	0.27	0.02	25.0	25.0	0.00	0.00	0.00	0.11	0.00	0.00	0.01	13.3	0.01	25.0	0.00	0.48	25.0	25.0	0.10	0.75	0.11	25.0	25.0	25.0	8.89	25.0	22.0
	001428	0.08	25.0	0.04	0.07	0.01	0.01	0.01	0.00	1.02	0.15	21.9	3.95	0.05	0.06	25.0	0.11	25.0	0.01	0.16	25.0	0.07	0.24	2.28	0.50	25.0	25.0	25.0	25.0	25.0	25.0
	JRCSF	0.97	0.15	0.19	0.63	0.02	0.11	0.06	0.00	0.03	0.00	25.0	25.0	0.04	0.01	0.62	0.06	0.08	0.02	12.8	0.42	0.04	0.06	1.52	0.17	25.0	25.0	25.0	1.96	25.0	25.0
	NAB13	0.30	25.0	7.24	0.02	0.00	25.0	0.01	7.98	2.67	0.03	25.0	25.0	25.0	25.0	25.0	2.90	25.0	0.02	25.0	25.0	25.0	0.02	0.08	0.00	25.0	2.56	25.0	25.0	25.0	1.30
	Q23.17	0.23	25.0	0.05	0.43	0.02	0.04	0.07	0.00	0.02	1.75	0.01	0.00	0.00	0.03	25.0	0.02	0.02	0.01	0.25	25.0	0.04	0.14	1.70	0.21	25.0	25.0	25.0	25.0	25.0	25.0
	Q842_D12	0.10	25.0	0.04	0.10	0.00	0.02	0.02	0.01	0.01	0.32	9.26	0.21	0.02	0.07	25.0	25.0	25.0	0.00	0.29	25.0	0.03	0.80	7.41	1.45	25.0	25.0	25.0	25.0	25.0	25.0
	REJO	0.51	5.41	0.84	0.28	0.05	0.06	0.04	2.37	0.23	0.00	25.0	25.0	25.0	25.0	25.0	25.0	25.0	0.16	8.31	25.0	0.02	0.04	1.07	0.11	25.0	4.74	25.0	0.88	25.0	23.1
	SF162	0.83	0.01	0.42	0.62	0.01	0.10	0.06	25.0	25.0	25.0	25.0	25.0	0.01	0.02	3.00	0.02	0.01	0.05	0.59	0.14	0.13	0.06	1.49	0.12	2.91	0.00	0.03	3.78	0.40	25.0
Env$^{\Delta CT}$ VSV^{pv}	MuLV	25.0	25.0	25.0	25.0	25.0	25.0	25.0	25.0	25.0	25.0	25.0	25.0	25.0	25.0	25.0	25.0	25.0	25.0	25.0	25.0	25.0	25.0	25.0	25.0	25.0	25.0	25.0	25.0	25.0	25.0
	Bal26	0.08	0.00	0.02	0.10	0.00	0.15	0.02	25.0	23.6	18.2	25.0	25.0	0.00	0.00	0.67	0.08	0.07	0.00	5.13	25.0	0.08	0.00	0.04	0.00	2.70	0.04	0.02	14.3	0.45	2.79
	BG505_T332N	0.18	25.0	0.04	0.32	0.02	0.04	0.04	0.00	0.02	0.01	0.24	0.00	0.02	0.01	0.46	0.13	0.04	0.00	0.06	0.00	0.06	0.00	0.05	0.00	25.0	25.0	25.0	22.3	25.0	2.47
	C1080	5.11	0.72	0.18	0.52	0.02	0.83	0.10	0.00	0.00	0.00	3.80	5.62	25.0	0.06	25.0	25.0	25.0	25.0	3.16	25.0	0.05	0.00	0.04	0.00	25.0	25.0	25.0	20.7	25.0	4.54
	CAP45	4.51	0.06	0.26	0.09	25.0	5.39	0.04	0.00	0.00	0.00	0.02	0.00	2.90	25.0	25.0	25.0	25.0	0.00	0.12	25.0	25.0	0.00	0.04	0.00	25.0	25.0	25.0	3.63	25.0	25.0
	CNE40	0.23	0.01	0.02	0.04	0.00	0.14	0.01	15.2	10.9	0.58	25.0	0.48	0.10	25.0	25.0	25.0	25.0	0.05	0.06	25.0	25.0	0.00	0.03	0.00	0.30	0.18	8.94	25.0	0.02	25.0
	CRF250	25.0	25.0	8.79	0.20	0.01	25.0	25.0	0.00	0.00	0.00	0.12	0.00	0.00	0.00	9.43	0.05	25.0	0.00	0.05	25.0	25.0	0.00	0.10	0.01	25.0	25.0	25.0	5.32	25.0	5.59
	001428	0.08	0.51	0.05	0.07	0.01	0.02	0.01	0.00	3.33	0.48	25.0	9.81	0.00	0.10	25.0	0.30	25.0	0.31	0.10	25.0	0.08	0.00	0.14	0.02	25.0	25.0	25.0	22.0	25.0	25.0
	JRCSF	0.67	0.05	0.07	0.62	0.03	0.02	0.02	0.00	0.06	0.00	5.99	25.0	0.03	0.01	1.02	0.31	0.06	0.02	6.49	0.02	0.06	0.00	0.08	0.00	25.0	25.0	25.0	4.87	25.0	3.07
	NAB13	0.02	25.0	25.0	0.16	0.00	25.0	0.00	25.0	25.0	0.16	25.0	25.0	25.0	25.0	0.95	25.0	25.0	25.0	25.0	25.0	25.0	0.00	0.00	0.00	0.89	0.00	25.0	25.0	25.0	0.01
	Q23.17	0.12	25.0	0.01	0.08	0.01	0.05	0.01	0.00	0.03	0.78	0.00	0.00	0.00	0.02	25.0	0.28	0.01	0.06	0.18	25.0	0.07	0.00	0.06	0.00	25.0	25.0	25.0	25.0	25.09	25.0
	Q842_D12	0.18	11.4	0.03	0.09	0.00	0.03	0.03	0.00	0.00	0.28	1.88	0.00	0.00	0.02	25.0	25.0	25.0	0.02	0.11	25.0	0.06	0.05	0.23	0.11	25.0	25.0	25.0	25.0	25.0	25.0
	REJO	0.33	0.00	0.25	0.28	0.02	0.03	0.01	0.03	0.07	0.00	2.38	25.0	25.0	25.0	25.0	25.0	25.0	0.05	3.01	25.0	0.03	0.00	0.01	0.03	25.0	1.05	25.0	0.49	25.0	25.0
	SF162	0.46	0.01	0.05	0.36	0.00	0.09	0.02	25.0	25.0	13.5	25.0	25.0	0.00	0.01	1.27	0.21	0.01	0.02	0.28	0.08	0.06	0.00	0.09	0.00	0.16	0.01	0.01	0.11	0.17	0.45
Env$^{\Delta CT}$ HIV^{pv}-WT	MuLV	25.0	25.0	25.0	25.0	25.0	25.0	25.0	25.0	25.0	25.0	25.0	25.0	25.0	25.0	25.0	25.0	25.0	25.0	25.0	25.0	25.0	25.0	25.0	25.0	25.0	25.0	25.0	25.0	25.0	25.0
	Bal26	0.08	0.00	0.02	0.11	0.00	0.14	0.01	25.0	3.22	6.32	25.0	25.0	0.01	0.02	0.33	0.08	0.04	0.01	3.47	25.0	0.11	0.00	0.06	0.02	13.5	0.02	0.01	11.8	2.39	2.47
	BG505_T332N	0.18	25.0	0.09	0.29	0.01	0.03	0.05	0.01	0.02	0.03	0.47	0.00	0.02	0.01	0.21	0.09	0.08	0.00	0.07	0.65	0.04	0.00	0.22	0.01	25.0	25.0	25.0	25.0	25.0	5.39
	C1080	2.33	3.47	1.15	0.66	0.02	1.96	0.05	0.00	0.00	0.08	2.49	2.21	25.0	0.10	25.0	25.0	25.0	11.1	0.60	2.73	0.03	0.00	0.01	0.00	25.0	25.0	25.0	15.7	25.0	0.24
	CAP45	8.67	0.03	0.22	0.15	25.0	4.78	0.05	0.00	0.00	0.00	0.03	0.00	2.95	25.0	25.0	25.0	25.0	0.00	0.07	25.0	25.0	0.00	0.06	0.01	25.0	25.0	25.0	2.08	25.0	25.0
	CNE40	0.65	0.24	0.06	0.10	0.01	0.14	0.02	25.0	9.44	0.37	25.0	0.39	0.34	25.0	25.0	25.0	25.0	0.01	0.07	25.0	25.0	0.00	0.01	0.01	0.30	0.21	15.8	25.0	0.07	25.0
	CRF250	25.0	25.0	25.0	0.05	0.02	25.0	25.0	0.00	0.00	0.01	0.10	0.00	0.00	0.01	4.75	0.08	25.0	0.00	0.20	25.0	25.0	0.00	0.09	0.02	25.0	4.93	25.0	23.2	25.0	3.80
	001428	0.07	3.19	0.05	0.06	0.01	0.01	0.00	0.00	4.83	0.35	25.0	25.0	0.06	0.11	25.0	0.46	25.0	0.28	0.22	25.0	0.06	0.01	0.22	0.02	25.0	25.0	25.0	25.0	25.0	25.0
	JRCSF	0.79	0.05	0.16	0.93	0.01	0.09	0.03	0.00	0.02	0.00	25.0	25.0	0.06	0.01	0.46	0.21	0.04	0.02	4.75	0.04	0.03	0.00	0.09	0.02	25.0	25.0	13.0	4.23	25.0	5.26
	NAB13	0.12	25.0	25.0	0.01	0.00	25.0	0.00	25.0	21.5	0.13	25.0	25.0	25.0	25.0	6.09	25.0	25.0	0.24	8.08	13.7	25.0	0.01	0.01	0.01	25.0	0.03	25.0	25.0	25.0	0.02
	Q23.17	0.21	25.0	0.06	0.17	0.01	0.03	0.03	0.00	0.03	1.95	0.00	0.29	0.01	0.03	25.0	0.12	0.01	0.02	0.13	25.0	0.06	0.00	0.04	0.02	25.0	25.0	25.0	25.0	25.0	25.0
	Q842_D12	0.09	25.0	0.03	0.08	0.00	0.01	0.01	0.01	0.00	0.40	10.0	0.15	0.01	0.05	25.0	25.0	25.0	0.02	0.11	25.0	0.02	0.09	0.73	0.07	25.0	25.0	25.0	25.0	25.0	25.0
	REJO	0.17	0.51	0.27	0.17	0.01	0.03	0.01	2.54	0.07	0.00	13.0	25.0	24.7	25.0	25.0	25.0	25.0	0.10	1.95	25.0	0.02	0.00	0.19	0.10	25.0	4.26	25.0	1.11	25.0	9.67
	SF162	0.66	0.00	0.34	0.30	0.00	0.03	0.02	25.0	25.0	25.0	25.0	25.0	0.00	0.01	1.64	0.10	0.01	0.03	0.32	0.01	0.07	0.00	0.18	0.01	1.13	0.00	0.01	0.46	0.22	2.09

Table S4: Plasma concentrations, protein binding and molecular weight of selected antiretroviral drugs, related to Figure 1 and 4. NRTI: Nucleoside reverse transcriptase inhibitor, NNRTI: Non-nucleoside reverse transcriptase inhibitor, PI: Protease inhibitor, INSTI: Integrase strand transfer inhibitor.

Antiretroviral drug	Abbreviation	Drug class	Peak level (mg/l)	Trough level (mg/l)	Protein binding (%)	Molecular weight (g/mol) [S25]
Abacavir	ABC	NRTI	3.1 [S26]	0.006 [S27]	50 [S28]	286.3
Emtricitabine	FTC	NRTI	1.9 [S29]	0.098 [S29]	<5 [S28]	247.3
Lamivudine	3TC	NRTI	1.7 [S26]	0.09 [S30]	<35 [S28]	229.3
Tenofovir	TAF/TDF	NRTI	0.31 [S29]	0.05 [S31]	<10 [S32]	287.2
Zidovudine	AZT	NRTI	1.3 [S26]	0.04 [S33]	34-38 [S28]	267.2
Doravirine	DOR	NNRTI	1.2 [S34]	0.38 [S34]	75 [S35]	425.7
Efavirenz	EFV	NNRTI	4.0 [S28]	1.7 [S28]	>99 [S28]	314.7
Rilpivirine	RPV	NNRTI	0.14 [S36]	0.069 [S37]	>99 [S38]	366.4
Atazanavir	ATV	PI	5.0 [S28]	2.0 [S28]	87 [S28]	704.9
Darunavir	DRV	PI	5.2 [S28]	2.2 [S28]	95 [S28]	547.7
Ritonavir	RTV	PI	11.2 [S28]	3.7 [S28]	89-99 [S28]	720.9
Bictegravir	BIC	INSTI	7.3 [S29]	2.6 [S29]	>99 [S39]	449.4
Dolutegravir	DTG	INSTI	3.9 [S40]	1.1 [S41]	>99 [S42]	419.4
Elvitegravir	EVG	INSTI	1.7 [S40]	0.46 [S43]	98-99 [S44]	447.9

Table S5: Drug combinations of PWH included in the screening for inhibitory activity by ARVs, related to Figure 5. 3TC: Lamivudine, ABC: Abacavir, ATV: Atazanavir, AZT: Zidovudine, BIC: Bictegravir, CAB: Cabotegravir, COB: Cobicistat, D4T: Stavudine, DDI: Didanosine, DOR: Doravirine, DRV: Darunavir, DTG: Dolutegravir, EFV: Efavirenz, ETV: Entecavir, EVG: Elvitegravir, FAPV: Fosamprenavir, FTC: Emtricitabine, IDV: Indinavir, LPV: Lopinavir, MK8591: Islatravir, MRV: Maraviroc, NFV: Nelfinavir, NVP: Nevirapine, RGV: Raltegravir, RPV: Rilpivirine, RTV: Ritonavir, SQV: Saquinavir, TAF/TDF/TXF: Tenofovir.

Screen	Participant ID	Drug combination	Plasma samples (N)	Samples with MuLV inhibition in standard TZM-bl assay (N)	Days from estimated date of infection to first ART initiation	Weeks on treatment	Drug concentration in plasma	
ZPHI screen	Z23	FTC + RPV + TAF	1	1	3824	117		
	Z32	DTG + FTC + RPV + TAF	1	1	28	169		
	Z37	COB + FTC + EVG + TAF	1	1	353	106		
	Z41	BIC +FTC + TAF	1	1	56	132		
	Z44	FTC + RPV + TDF	1	1	23	74		
	Z52	DRV + FTC + RTV + TDF	1	0	25	179	DRV:	4.0 mg/l
	Z96	3TC + AZT + EFV	1	1	56	18	EFV:	8.1 mg/l
	Z97	3TC + ABC + EFV	1	1	44	34	EFV:	1.3 mg/l
	Z98	3TC + ABC + DRV + RTV	1	0	81	56	DRV:	6.5 mg/l
	Z99	EFV + FTC + TDF	1	1	47	197	EFV:	2.0 mg/l
	Z100	DRV + FTC + RTV + TDF	1	0	29	54	DRV:	0.9 mg/l
	Z101	ATV + EFV + FTC + RTV + TDF	1	1	35	131	EFV:	3.0 mg/l
	Z102	DRV + FTC + RTV + TDF	1	0	173	107	DRV:	2.5 mg/l
	Z103	DTG + FTC + TDF	1	1	145	29	DTG:	0.3 mg/l
	Z104	COB + FTC + EVG + TDF	1	1	59	73		
	Z105	DTG + FTC + TAF	1	1	29	56	DTG:	1.8 mg/l
	Z106	COB + FTC + EVG + TAF	1	1	34	105		
	Z107	DTG + FTC + TAF	1	1	24	136	DTG:	3.2 mg/l
	Z108	DTG	1	1	184	85	DTG:	10.6 mg/l
	Z109	BIC + FTC + TAF	1	1	47	94		
	Z110	BIC + DOR + FTC + TAF	1	1	34	73		
	Z263	CAB + RPV	1	1	48	14		
	Z264	CAB + RPV	1	1	103	14		
Extended screen		BIC + FTC + TAF	68	65				
		EFV + FTC + TDF	48	45				
		FTC + RPV + TXF	46	43				
		DRV + FTC + RTV + TDF	38	0				
		ATV + FTC + RTV + TDF	37	2				
		3TC + DTG	27	27				
		DTG + FTC + TXF	24	24				
		3TC + ABC + DTG	18	18				
		COB + FTC + EVG + TXF	17	16				
		3TC + AZT + LPV	14	2				

		FTC + LPV + TDF	14	1				
		FTC + NVP + TXF	13	12				
		FTC + RGV + TXF	16	5				
		DTG + FTC + RPV + TAF	10	10				
		3TC + ABC + ATV + RTV	8	0				
		3TC + ABC + EFV	8	8				
		3TC + ABC + RGV	8	4				
		3TC + AZT + LPV + RTV	8	0				
		3TC + AZT + EFV	7	7				
		3TC + AZT + DRV + RGV + RTV + TDF	6	1				
		BIC + DOR + FTC + TAF	6	6				
		3TC + ABC + DRV + RTV	5	0				
		3TC + AZT + NVP	5	3				
		COB + DRV + FTC + TAF	5	1				
		3TC + ABC + NVP	4	4				
		3TC + NVP	4	4				
		COB + DRV + FTC + EVG + TAF	4	4				
		3TC + ABC + LPV	3	0				
		3TC + AZT + NFV	3	0				
		ATV + COB + FTC + TDF	3	0				
		DRV + FTC + RGV + RTV + TXF	3	0				
		3TC + ABC + D4T	2	0				
		3TC + ABC + EFV + LPV	2	2				
		3TC + ABC + LPV + NVP + TDF	2	1				
		3TC + ABC + LPV + RTV	2	0				
		3TC + ATV + RTV + TDF	2	0				
		3TC + AZT	2	0				
		3TC + AZT + EFV + TDF	2	2				
		3TC + DOR + TDF	2	2				
		3TC + NFV + TDF	2	0				
		ATV + DDI + RTV + TDF	2	0				
		AZT + DRV + RGV + RTV	2	1				
		BIC + DRV + FTC + RTV + TAF	2	2				
		CAB + RPV	2	2				
		COB + DRV + DTG	2	2				
		D4T + DDI + EFV + LPV	2	2				
		D4T + DDI + NFV	2	0				
		DOR + DRV + RGV + RTV	2	2				
		DOR + DTG	2	2				
		DRV + ETV + RGV + RTV	2	2				
		DTG + FTC	2	2				
		DTG + FTC + ETV + TAF	2	2				

		DTG + RPV	2	2				
		EFV + LPV + RTV + TDF	2	2				
		ETV + RGV + TDF	2	2				
		FAPV + MRV + RGV + RTV	2	1				
		FTC + FAPV + RTV + TDF	2	0				
		FTC + LPV + RTV + TDF	2	0				
		FTC + MRV + RGV + TAF	2	0				
		FTC + NFV + TDF	2	0				
		3TC + ABC + ATV + RTV + TDF	1	0				
		3TC + ABC + AZT	1	0				
		3TC + ABC + DOR + DTG	1	1				
		3TC + ABC + DRV + RGV + RTV	1	1				
		3TC + AZT + FAPV + RTV + TDF	1	0				
		3TC + DDI + EFV	1	1				
		3TC + DDI + NVP	1	1				
		3TC + DOR + DTG	1	1				
		3TC + LPV + TDF	1	0				
		ABC + ATV + FTC + RTV + TDF	1	0				
		ABC + DDI + EFV	1	1				
		ATV + DDI + FTC + RTV + TDF	1	0				
		ATV + EFV + FTC + RTV + TDF	1	1				
		AZT	1	0				
		AZT + DDI	1	0				
		AZT + FAPV + LPV + TDF	1	0				
		AZT + IDV + NVP + TDF	1	1				
		COB + DRV + DTG + FTC + TAF	1	1				
		D4T + DDI + EFV	1	1				
		D4T + RTV + SQV	1	0				
		DRV + RGV + RTV	1	1				
		DTG	1	1				
		EFV + FAPV + RTV + TDF	1	1				
		FTC + TDF	1	0				
		MK8591	1	1				

Table S6: Neutralization titers (NT50) of longitudinal plasma samples from known bnAb inducers, related to Figure 6.

Subject	Group	Year	MuLV	BG505_T332N	Q23.17	Q842_D12	SF162	Bal26	JRCSF	REJO	CAP45	001428	NAB13	C1080	CRF250	CNE40	Potency (Geometric mean NT50)	Breadth (%)
S5206-G5	HIV^{pv}-WT	2007	100	1374	26238	5973	919	227	100	213	8060	248	293	16305	7893	4050	1944	92
S5206-G5	HIV^{pv}-WT	2009	100	2437	44446	13091	874	265	100	131	21911	870	320	43615	27296	21043	3699	92
S5206-G5	HIV^{pv}-WT	2010	100	859	46941	7559	731	159	100	100	13107	479	418	20554	8682	7452	2903	85
S5206-G5	HIV^{pv}-WT	2011	100	1867	38827	8671	1311	148	100	100	24004	412	284	22113	13672	6121	3368	85
S5206-G5	HIV^{pv}-WT	2012	100	653	20377	3108	436	100	100	100	10321	407	124	20962	7806	6100	2495	77
S5206-G5	HIV^{pv}-WT	2013	294	877	12815	5243	1315	364	322	470	7095	732	380	11188	7221	3866	1833	100
S5206-G5	HIV^{pv}-MDR8	2007	100	1210	33141	5234	859	921	100	211	7207	875	148	18269	6511	7114	2345	92
S5206-G5	HIV^{pv}-MDR8	2009	100	941	128320	1547	854	203	100	103	8731	1321	312	47060	6659	32367	2655	92
S5206-G5	HIV^{pv}-MDR8	2010	100	273	171406	2254	470	200	100	100	24817	695	148	18729	6032	27919	2791	85
S5206-G5	HIV^{pv}-MDR8	2011	100	530	192222	6998	1059	581	100	131	10284	1212	190	25984	10249	4107	2698	92
S5206-G5	HIV^{pv}-MDR8	2012	100	280	84621	5097	399	194	100	100	37759	539	100	24194	5846	3436	3138	77
S5206-G5	HIV^{pv}-MDR8	2013	100	100	37826	547	288	101	100	100	1819	1167	100	7466	4242	3198	1718	69
S5206-G5	HIV^{pv}-MDR13	2007	100	636	93764	8369	931	267	100	100	6412	445	150	19331	6770	3452	2502	85
S5206-G5	HIV^{pv}-MDR13	2009	100	1459	79085	6375	1076	236	100	143	27343	1449	181	34072	10644	12291	3120	92
S5206-G5	HIV^{pv}-MDR13	2010	100	751	70463	4748	392	137	100	100	7377	1235	185	14289	11447	6083	2487	85
S5206-G5	HIV^{pv}-MDR13	2011	131	810	69096	5446	244	223	100	132	8856	1053	100	8843	5977	7024	2253	85
S5206-G5	HIV^{pv}-MDR13	2012	100	816	126008	8045	888	129	100	100	12973	945	117	16559	8469	4949	2832	85
S5206-G5	HIV^{pv}-MDR13	2013	100	1275	46013	2149	208	106	100	100	5309	1115	102	8181	5670	2345	1605	85
S5206-G5	VSV^{pv}	2007	100	659	17994	7939	4438	1507	351	632	9026	162	8996	87160	13753	5629	3514	100
S5206-G5	VSV^{pv}	2009	100	1175	152845	8211	13414	5000	4990	535	23773	691	6659	28826	31972	7516	7402	100
S5206-G5	VSV^{pv}	2010	100	2008	42290	5944	1724	1199	332	241	10799	726	19805	10026	10872	6501	3433	100
S5206-G5	VSV^{pv}	2011	100	4043	21547	6106	3891	3158	216	201	15493	123	4396	32334	17084	5423	3366	100
S5206-G5	VSV^{pv}	2012	100	1047	37618	10943	3627	1471	125	242	16695	159	6670	10823	26281	24410	3414	100
S5206-G5	VSV^{pv}	2013	100	839	6389	3598	1035	213	100	100	4880	136	1869	7872	33053	1185	1959	85
S5206-G5	Protein A/G	2007	100	100	394	113	262	106	100	100	251	100	100	1311	229	1067	316	62
S5206-G5	Protein A/G	2009	100	100	226	100	135	100	100	100	116	100	131	1225	100	1029	289	46
S5206-G5	Protein A/G	2010	100	100	264	100	245	100	100	100	113	100	100	570	100	1247	350	38
S5206-G5	Protein A/G	2011	100	100	317	100	286	100	100	100	176	100	100	749	100	1338	437	38
S5206-G5	Protein A/G	2012	100	100	168	100	156	100	100	100	134	100	100	1057	154	1412	305	46
S5206-G5	Protein A/G	2013	100	100	255	100	104	100	100	100	186	100	100	604	142	671	257	46
S5206-G5	ART-DEX	2007	100	899	32051	5831	412	160	167	114	15219	886	560	32642	9870	18490	2023	100
S5206-G5	ART-DEX	2009	100	1787	79733	8857	889	260	100	184	40084	1937	1180	81359	20150	16817	4761	92
S5206-G5	ART-DEX	2010	100	711	47072	4634	674	150	100	126	13558	997	439	18538	24248	8900	2451	92
S5206-G5	ART-DEX	2011	100	523	35894	6560	372	208	100	100	13564	822	304	45063	21820	12295	3223	85
S5206-G5	ART-DEX	2012	100	1107	55318	4317	595	148	333	100	26650	531	162	34589	13380	12375	2614	92
S5206-G5	ART-DEX	2013	100	402	25857	2213	226	100	100	100	10990	1293	100	13952	10071	7767	3509	69
S5206-G5	ART-DEX + HIV^{pv}-MDR8	2007	100	247	210986	1156	672	398	100	306	2311	2444	100	148197	15697	11204	3410	85
S5206-G5	ART-DEX + HIV^{pv}-MDR8	2009	100	151	693244	1071	797	477	100	210	79321	2014	100	39330	185584	10249	5370	85
S5206-G5	ART-DEX + HIV^{pv}-MDR8	2010	100	166	389749	541	533	285	100	100	132605	592	141	22135	5593	2301	2422	85
S5206-G5	ART-DEX + HIV^{pv}-MDR8	2011	100	388	124297	24679	938	313	100	135	6147	1084	130	17748	8202	6075	2397	92
S5206-G5	ART-DEX + HIV^{pv}-MDR8	2012	100	744	164823	1453	453	110	100	100	19628	472	100	18510	7379	918	2521	77
S5206-G5	ART-DEX + HIV^{pv}-MDR8	2013	100	100	34228	231	117	117	100	100	3578	1526	100	59078	1668	5869	1913	69
S5206-G5	ART-DEX + HIV^{pv}-MDR13	2007	100	592	29927	3763	371	230	100	100	8870	345	193	14643	9585	4993	2025	85
S5206-G5	ART-DEX + HIV^{pv}-MDR13	2009	100	1586	103933	7837	873	191	100	100	24242	1047	257	67017	30654	6768	4600	85
S5206-G5	ART-DEX + HIV^{pv}-MDR13	2010	100	689	62486	3973	597	112	100	100	9854	1122	242	12227	12774	3474	2418	85
S5206-G5	ART-DEX + HIV^{pv}-MDR13	2011	100	547	38224	3109	615	100	100	100	9297	755	100	17283	11550	3146	3827	69
S5206-G5	ART-DEX + HIV^{pv}-MDR13	2012	100	265	35435	3476	236	100	100	100	10160	484	100	5399	8388	1548	2402	69
S5206-G5	ART-DEX + HIV^{pv}-MDR13	2013	100	396	38538	1920	257	100	100	100	7741	627	100	7970	3806	1779	2323	69
S51517	HIV^{pv}-WT	2008	100	666	427	4085	11986	1655	2671	3235	302	4377	984	555	294	4403	1495	100
S51517	HIV^{pv}-WT	2010	100	261	203	699	5898	843	411	577	100	624	393	266	100	2818	645	85
S51517	HIV^{pv}-WT	2011	100	182	132	299	3872	307	295	456	100	408	293	114	100	3048	405	85
S51517	HIV^{pv}-WT	2012	100	180	164	646	7947	349	357	601	101	308	284	185	100	2274	432	92
S51517	HIV^{pv}-WT	2013	142	399	215	529	4297	393	463	495	274	355	344	384	243	1921	494	100
S51517	HIV^{pv}-WT	2014	125	222	173	369	3032	461	449	444	232	370	226	247	249	1739	406	100
S51517	HIV^{pv}-MDR8	2008	100	302	1418	2598	5556	1663	2393	4543	290	6857	931	207	141	2913	1246	100
S51517	HIV^{pv}-MDR8	2010	100	100	286	318	1834	1833	191	810	100	873	197	100	100	2928	660	69
S51517	HIV^{pv}-MDR8	2011	100	451	122	429	2753	519	130	411	100	854	175	100	100	1320	452	77
S51517	HIV^{pv}-MDR8	2012	100	100	123	482	2700	676	289	464	100	432	241	152	100	1822	459	77
S51517	HIV^{pv}-MDR8	2013	100	100	100	110	2435	809	100	173	100	153	100	100	100	643	393	46
S51517	HIV^{pv}-MDR8	2014	100	100	100	100	993	232	100	100	100	203	100	100	100	1178	484	31
S51517	HIV^{pv}-MDR13	2008	100	743	572	1860	7053	1455	1448	2822	244	3036	366	718	164	1846	1053	100
S51517	HIV^{pv}-MDR13	2010	100	306	300	583	4330	758	763	798	100	991	170	283	100	3267	689	85
S51517	HIV^{pv}-MDR13	2011	100	109	119	398	2488	424	308	645	100	570	196	131	100	2083	391	85
S51517	HIV^{pv}-MDR13	2012	100	401	125	256	1482	1028	227	369	150	270	138	237	100	1971	357	92
S51517	HIV^{pv}-MDR13	2013	218	290	204	153	2485	401	276	480	236	280	209	314	256	2189	375	100
S51517	HIV^{pv}-MDR13	2014	100	155	128	240	1029	296	227	252	100	184	245	121	118	1256	252	92
S51517	VSV^{pv}	2008	100	3240	100	5462	22905	11457	2403	5948	960	1487	7849	2060	100	2342	3943	85
S51517	VSV^{pv}	2010	100	568	1134	2828	19403	2550	777	883	1477	623	2662	1221	387	3437	1527	100
S51517	VSV^{pv}	2011	100	196	104	618	12391	1340	831	353	100	131	2252	158	100	1372	604	85
S51517	VSV^{pv}	2012	100	295	100	4273	4699	3505	227	548	100	171	733	763	100	4757	1016	77

		2013	100	100	100	328	5963	1219	100	100	210	209	2005	142	100	858	632	62
		2014	100	100	100	325	12553	367	100	142	100	100	2331	130	100	2368	764	54
	Protein A/G	2008	100	189	100	690	1982	217	318	973	100	1224	136	334	100	458	462	77
		2010	100	100	100	132	1481	164	100	125	100	194	100	139	100	628	254	54
		2011	100	100	100	100	1649	111	100	111	100	121	100	140	100	372	225	46
		2012	100	100	100	100	1290	183	100	100	100	105	100	215	100	555	312	38
		2013	100	100	100	100	937	106	100	100	100	100	100	100	100	314	315	23
		2014	100	100	100	100	889	100	100	100	100	100	100	100	100	127	336	15
	ART-DEX	2008	100	1222	337	3274	5635	1450	2521	1822	328	4385	1481	633	217	8322	1448	100
		2010	100	239	179	360	5311	711	391	578	200	842	307	322	100	6789	581	92
		2011	100	230	100	317	5189	520	401	459	104	525	455	188	100	3023	502	85
		2012	100	172	100	452	4161	503	182	330	124	366	382	344	100	2737	452	85
		2013	100	159	100	251	1698	267	100	200	100	224	144	247	100	4535	370	69
		2014	100	103	100	168	1493	168	103	248	100	217	113	305	100	1307	253	77
	ART-DEX + HIV^{pv}-MDR8	2008	100	802	684	3133	3545	1604	578	4385	100	2939	345	918	100	883	1315	85
		2010	100	163	282	100	4725	1040	541	1031	100	604	176	289	100	911	568	77
		2011	100	100	224	158	4009	684	163	712	100	351	149	100	100	910	433	69
		2012	100	159	122	182	1577	510	229	401	100	289	170	489	100	852	332	85
		2013	100	134	100	100	2207	418	100	363	100	150	100	100	100	453	381	46
		2014	100	100	100	100	1089	257	100	145	100	100	100	100	100	1043	453	31
	ART-DEX + HIV^{pv}-MDR13	2008	100	724	270	2395	4719	893	1403	2032	134	2682	256	849	135	2843	872	100
		2010	100	187	125	637	5923	491	293	384	100	588	104	279	150	4793	445	92
		2011	100	122	104	270	3411	239	225	342	100	417	236	100	100	3721	389	77
		2012	100	421	100	224	2911	335	174	284	252	220	229	193	100	864	350	85
		2013	100	127	100	278	1985	230	141	183	100	182	107	199	123	666	237	85
		2014	100	106	100	217	874	234	225	143	105	122	154	173	180	513	203	92

Table S7: Summary of tested ART-free neutralization methods, related to Figure 5, 6, and Discussion.

**ART-free neutralization method**	**Advantages**	**Disadvantages**
HIV^{pv}-MDR8	No additional sample preparation, use with standard TZM-bl assay	Low infectivity, residual inhibitory activity (especially for second generation INSTIs)
HIV^{pv}-MDR13	No additional sample preparation, use with standard TZM-bl assay	Low infectivity, residual inhibitory activity (especially for EFV-containing regimen)
VSV^{pv}	Complete resistance against all groups of HIV enzyme-targeting ARVs	Increase in sensitivity compared to standard TZM-bl assay using HIV^{pv}-WT, possibly differences in entry process as based on VSV backbone
Protein A/G + HIV^{pv}-WT	Compatibility with all groups of ARVs except antibody-based entry inhibitors	Additional sample preparation needed, loss of antibodies, need for normalized antibody input
ART-DEX + HIV^{pv}-WT	Use with HIV^{pv}-WT, good correlation with titers obtained with standard TZM-bl assay	Incomplete removal of ARVs (especially EFV and second-generation INSTIs), sample preparation needed
ART-DEX + HIV^{pv}-MDR8	Removal of inhibitory activity of ARVs (especially EFV)	Low infectivity, sample preparation needed
ART-DEX + HIV^{pv}-MDR13	Complete removal of inhibitory activity of ARVs (especially second-generation INSTIs), good correlation with titers obtained with standard TZM-bl assay	Low infectivity, sample preparation needed

References

1. Schommers, P., Gruell, H., Abernathy, M.E., Tran, M.K., Dingens, A.S., Gristick, H.B., Barnes, C.O., Schoofs, T., Schlotz, M., Vanshylla, K., Kreer, C., Weiland, D., Holtick, U., Scheid, C., Valter, M.M., van Gils, M.J., Sanders, R.W., Vehreschild, J.J., Cornely, O.A., Lehmann, C., Fatkenheuer, G., Seaman, M.S., Bloom, J.D., Bjorkman, P.J., and Klein, F. (2020). Restriction of HIV-1 Escape by a Highly Broad and Potent Neutralizing Antibody. Cell *180*, 471-489 e422. 10.1016/j.cell.2020.01.010.
2. Burton, D.R., Barbas, C.F., 3rd, Persson, M.A., Koenig, S., Chanock, R.M., and Lerner, R.A. (1991). A large array of human monoclonal antibodies to type 1 human immunodeficiency virus from combinatorial libraries of asymptomatic seropositive individuals. Proc Natl Acad Sci U S A *88*, 10134-10137. 10.1073/pnas.88.22.10134.
3. Bonsignori, M., Zhou, T., Sheng, Z., Chen, L., Gao, F., Joyce, M.G., Ozorowski, G., Chuang, G.Y., Schramm, C.A., Wiehe, K., Alam, S.M., Bradley, T., Gladden, M.A., Hwang, K.K., Iyengar, S., Kumar, A., Lu, X., Luo, K., Mangiapani, M.C., Parks, R.J., Song, H., Acharya, P., Bailer, R.T., Cao, A., Druz, A., Georgiev, I.S., Kwon, Y.D., Louder, M.K., Zhang, B., Zheng, A., Hill, B.J., Kong, R., Soto, C., Program, N.C.S., Mullikin, J.C., Douek, D.C., Montefiori, D.C., Moody, M.A., Shaw, G.M., Hahn, B.H., Kelsoe, G., Hraber, P.T., Korber, B.T., Boyd, S.D., Fire, A.Z., Kepler, T.B., Shapiro, L., Ward, A.B., Mascola, J.R., Liao, H.X., Kwong, P.D., and Haynes, B.F. (2016). Maturation Pathway from Germline to Broad HIV-1 Neutralizer of a CD4-Mimic Antibody. Cell *165*, 449-463. 10.1016/j.cell.2016.02.022.
4. Sajadi, M.M., Dashti, A., Rikhtegaran Tehrani, Z., Tolbert, W.D., Seaman, M.S., Ouyang, X., Gohain, N., Pazgier, M., Kim, D., Cavet, G., Yared, J., Redfield, R.R., Lewis, G.K., and DeVico, A.L. (2018). Identification of Near-Pan-neutralizing Antibodies against HIV-1 by Deconvolution of Plasma Humoral Responses. Cell *173*, 1783-1795 e1714. 10.1016/j.cell.2018.03.061.
5. Huang, J., Kang, B.H., Ishida, E., Zhou, T., Griesman, T., Sheng, Z., Wu, F., Doria-Rose, N.A., Zhang, B., McKee, K., O'Dell, S., Chuang, G.Y., Druz, A., Georgiev, I.S., Schramm, C.A., Zheng, A., Joyce, M.G., Asokan, M., Ransier, A., Darko, S., Migueles, S.A., Bailer, R.T., Louder, M.K., Alam, S.M., Parks, R., Kelsoe, G., Von Holle, T., Haynes, B.F., Douek, D.C., Hirsch, V., Seaman, M.S., Shapiro, L., Mascola, J.R., Kwong, P.D., and Connors, M. (2016). Identification of a CD4-Binding-Site Antibody to HIV that Evolved Near-Pan Neutralization Breadth. Immunity *45*, 1108-1121. 10.1016/j.immuni.2016.10.027.
6. Wu, X., Yang, Z.Y., Li, Y., Hogerkorp, C.M., Schief, W.R., Seaman, M.S., Zhou, T., Schmidt, S.D., Wu, L., Xu, L., Longo, N.S., McKee, K., O'Dell, S., Louder, M.K., Wycuff, D.L., Feng, Y., Nason, M., Doria-Rose, N., Connors, M., Kwong, P.D., Roederer, M., Wyatt, R.T., Nabel, G.J., and Mascola, J.R. (2010). Rational design of envelope identifies broadly neutralizing human monoclonal antibodies to HIV-1. Science *329*, 856-861. 10.1126/science.1187659.
7. Wu, X., Zhou, T., Zhu, J., Zhang, B., Georgiev, I., Wang, C., Chen, X., Longo, N.S., Louder, M., McKee, K., O'Dell, S., Perfetto, S., Schmidt, S.D., Shi, W., Wu, L., Yang, Y., Yang, Z.Y., Yang, Z., Zhang, Z., Bonsignori, M., Crump, J.A., Kapiga, S.H., Sam, N.E., Haynes, B.F., Simek, M., Burton, D.R., Koff, W.C., Doria-Rose, N.A., Connors, M., Program, N.C.S., Mullikin, J.C., Nabel, G.J., Roederer, M., Shapiro, L., Kwong, P.D., and Mascola, J.R. (2011). Focused evolution of HIV-1 neutralizing antibodies revealed by structures and deep sequencing. Science *333*, 1593-1602. 10.1126/science.1207532.
8. Thali, M., Moore, J.P., Furman, C., Charles, M., Ho, D.D., Robinson, J., and Sodroski, J. (1993). Characterization of conserved human immunodeficiency virus type 1 gp120 neutralization epitopes exposed upon gp120-CD4 binding. J Virol *67*, 3978-3988. 10.1128/JVI.67.7.3978-3988.1993.
9. Bonsignori, M., Hwang, K.K., Chen, X., Tsao, C.Y., Morris, L., Gray, E., Marshall, D.J., Crump, J.A., Kapiga, S.H., Sam, N.E., Sinangil, F., Pancera, M., Yongping, Y., Zhang, B., Zhu, J., Kwong, P.D., O'Dell, S., Mascola, J.R., Wu, L., Nabel, G.J., Phogat, S., Seaman, M.S., Whitesides, J.F., Moody, M.A., Kelsoe, G., Yang, X., Sodroski, J., Shaw, G.M., Montefiori, D.C., Kepler, T.B., Tomaras, G.D., Alam, S.M., Liao, H.X., and Haynes, B.F. (2011). Analysis of a clonal lineage of HIV-1 envelope V2/V3 conformational epitope-specific broadly neutralizing antibodies and their inferred unmutated common ancestors. J Virol *85*, 9998-10009. 10.1128/JVI.05045-11.

10. Walker, L.M., Phogat, S.K., Chan-Hui, P.Y., Wagner, D., Phung, P., Goss, J.L., Wrin, T., Simek, M.D., Fling, S., Mitcham, J.L., Lehrman, J.K., Priddy, F.H., Olsen, O.A., Frey, S.M., Hammond, P.W., Protocol, G.P.I., Kaminsky, S., Zamb, T., Moyle, M., Koff, W.C., Poignard, P., and Burton, D.R. (2009). Broad and potent neutralizing antibodies from an African donor reveal a new HIV-1 vaccine target. Science *326*, 285-289. 10.1126/science.1178746.
11. Sok, D., van Gils, M.J., Pauthner, M., Julien, J.P., Saye-Francisco, K.L., Hsueh, J., Briney, B., Lee, J.H., Le, K.M., Lee, P.S., Hua, Y., Seaman, M.S., Moore, J.P., Ward, A.B., Wilson, I.A., Sanders, R.W., and Burton, D.R. (2014). Recombinant HIV envelope trimer selects for quaternary-dependent antibodies targeting the trimer apex. Proc Natl Acad Sci U S A *111*, 17624-17629. 10.1073/pnas.1415789111.
12. Walker, L.M., Huber, M., Doores, K.J., Falkowska, E., Pejchal, R., Julien, J.P., Wang, S.K., Ramos, A., Chan-Hui, P.Y., Moyle, M., Mitcham, J.L., Hammond, P.W., Olsen, O.A., Phung, P., Fling, S., Wong, C.H., Phogat, S., Wrin, T., Simek, M.D., Protocol, G.P.I., Koff, W.C., Wilson, I.A., Burton, D.R., and Poignard, P. (2011). Broad neutralization coverage of HIV by multiple highly potent antibodies. Nature *477*, 466-470. 10.1038/nature10373.
13. Doria-Rose, N.A., Bhiman, J.N., Roark, R.S., Schramm, C.A., Gorman, J., Chuang, G.Y., Pancera, M., Cale, E.M., Ernandes, M.J., Louder, M.K., Asokan, M., Bailer, R.T., Druz, A., Fraschilla, I.R., Garrett, N.J., Jarosinski, M., Lynch, R.M., McKee, K., O'Dell, S., Pegu, A., Schmidt, S.D., Staupe, R.P., Sutton, M.S., Wang, K., Wibmer, C.K., Haynes, B.F., Abdool-Karim, S., Shapiro, L., Kwong, P.D., Moore, P.L., Morris, L., and Mascola, J.R. (2016). New Member of the V1V2-Directed CAP256-VRC26 Lineage That Shows Increased Breadth and Exceptional Potency. J Virol *90*, 76-91. 10.1128/JVI.01791-15.
14. Scheid, J.F., Mouquet, H., Feldhahn, N., Seaman, M.S., Velinzon, K., Pietzsch, J., Ott, R.G., Anthony, R.M., Zebroski, H., Hurley, A., Phogat, A., Chakrabarti, B., Li, Y., Connors, M., Pereyra, F., Walker, B.D., Wardemann, H., Ho, D., Wyatt, R.T., Mascola, J.R., Ravetch, J.V., and Nussenzweig, M.C. (2009). Broad diversity of neutralizing antibodies isolated from memory B cells in HIV-infected individuals. Nature *458*, 636-640. 10.1038/nature07930.
15. Buchacher, A., Predl, R., Strutzenberger, K., Steinfellner, W., Trkola, A., Purtscher, M., Gruber, G., Tauer, C., Steindl, F., Jungbauer, A., and et al. (1994). Generation of human monoclonal antibodies against HIV-1 proteins; electrofusion and Epstein-Barr virus transformation for peripheral blood lymphocyte immortalization. AIDS Res Hum Retroviruses *10*, 359-369. 10.1089/aid.1994.10.359.
16. Buchbinder, A., Zolla-Pazner, S., Karwowska, S., Gorny, M.K., and Burda, S.T. (1992). Synergy between human monoclonal antibodies to HIV extends their effective biologic activity against homologous and divergent strains. AIDS Res Hum Retroviruses *8*, 1395. 10.1089/aid.1992.8.1395.
17. Sok, D., Pauthner, M., Briney, B., Lee, J.H., Saye-Francisco, K.L., Hsueh, J., Ramos, A., Le, K.M., Jones, M., Jardine, J.G., Bastidas, R., Sarkar, A., Liang, C.H., Shivatare, S.S., Wu, C.Y., Schief, W.R., Wong, C.H., Wilson, I.A., Ward, A.B., Zhu, J., Poignard, P., and Burton, D.R. (2016). A Prominent Site of Antibody Vulnerability on HIV Envelope Incorporates a Motif Associated with CCR5 Binding and Its Camouflaging Glycans. Immunity *45*, 31-45. 10.1016/j.immuni.2016.06.026.
18. van Gils, M.J., van den Kerkhof, T.L., Ozorowski, G., Cottrell, C.A., Sok, D., Pauthner, M., Pallesen, J., de Val, N., Yasmeen, A., de Taeye, S.W., Schorcht, A., Gumbs, S., Johanna, I., Saye-Francisco, K., Liang, C.H., Landais, E., Nie, X., Pritchard, L.K., Crispin, M., Kelsoe, G., Wilson, I.A., Schuitemaker, H., Klasse, P.J., Moore, J.P., Burton, D.R., Ward, A.B., and Sanders, R.W. (2016). An HIV-1 antibody from an elite neutralizer implicates the fusion peptide as a site of vulnerability. Nat Microbiol 2, 16199. 10.1038/nmicrobiol.2016.199.
19. Falkowska, E., Le, K.M., Ramos, A., Doores, K.J., Lee, J.H., Blattner, C., Ramirez, A., Derking, R., van Gils, M.J., Liang, C.H., McBride, R., von Bredow, B., Shivatare, S.S., Wu, C.Y., Chan-Hui, P.Y., Liu, Y., Feizi, T., Zwick, M.B., Koff, W.C., Seaman, M.S., Swiderek, K., Moore, J.P., Evans, D., Paulson, J.C., Wong, C.H., Ward, A.B., Wilson, I.A., Sanders, R.W., Poignard, P., and Burton, D.R. (2014). Broadly neutralizing HIV antibodies define a glycan-dependent epitope on the prefusion conformation of gp41 on cleaved envelope trimers. Immunity *40*, 657-668. 10.1016/j.immuni.2014.04.009.

20. Kong, R., Xu, K., Zhou, T., Acharya, P., Lemmin, T., Liu, K., Ozorowski, G., Soto, C., Taft, J.D., Bailer, R.T., Cale, E.M., Chen, L., Choi, C.W., Chuang, G.Y., Doria-Rose, N.A., Druz, A., Georgiev, I.S., Gorman, J., Huang, J., Joyce, M.G., Louder, M.K., Ma, X., McKee, K., O'Dell, S., Pancera, M., Yang, Y., Blanchard, S.C., Mothes, W., Burton, D.R., Koff, W.C., Connors, M., Ward, A.B., Kwong, P.D., and Mascola, J.R. (2016). Fusion peptide of HIV-1 as a site of vulnerability to neutralizing antibody. Science *352*, 828-833. 10.1126/science.aae0474.

21. Schoofs, T., Barnes, C.O., Suh-Toma, N., Golijanin, J., Schommers, P., Gruell, H., West, A.P., Jr., Bach, F., Lee, Y.E., Nogueira, L., Georgiev, I.S., Bailer, R.T., Czartoski, J., Mascola, J.R., Seaman, M.S., McElrath, M.J., Doria-Rose, N.A., Klein, F., Nussenzweig, M.C., and Bjorkman, P.J. (2019). Broad and Potent Neutralizing Antibodies Recognize the Silent Face of the HIV Envelope. Immunity *50*, 1513-1529. 10.1016/j.immuni.2019.04.014.

22. Huang, J., Ofek, G., Laub, L., Louder, M.K., Doria-Rose, N.A., Longo, N.S., Imamichi, H., Bailer, R.T., Chakrabarti, B., Sharma, S.K., Alam, S.M., Wang, T., Yang, Y., Zhang, B., Migueles, S.A., Wyatt, R., Haynes, B.F., Kwong, P.D., Mascola, J.R., and Connors, M. (2012). Broad and potent neutralization of HIV-1 by a gp41-specific human antibody. Nature *491*, 406-412. 10.1038/nature11544.

23. Williams, L.D., Ofek, G., Schatzle, S., McDaniel, J.R., Lu, X., Nicely, N.I., Wu, L., Lougheed, C.S., Bradley, T., Louder, M.K., McKee, K., Bailer, R.T., O'Dell, S., Georgiev, I.S., Seaman, M.S., Parks, R.J., Marshall, D.J., Anasti, K., Yang, G., Nie, X., Tumba, N.L., Wiehe, K., Wagh, K., Korber, B., Kepler, T.B., Munir Alam, S., Morris, L., Kamanga, G., Cohen, M.S., Bonsignori, M., Xia, S.M., Montefiori, D.C., Kelsoe, G., Gao, F., Mascola, J.R., Moody, M.A., Saunders, K.O., Liao, H.X., Tomaras, G.D., Georgiou, G., and Haynes, B.F. (2017). Potent and broad HIV-neutralizing antibodies in memory B cells and plasma. Sci Immunol *2*. 10.1126/sciimmunol.aal2200.

24. Nelson, J.D., Brunel, F.M., Jensen, R., Crooks, E.T., Cardoso, R.M., Wang, M., Hessell, A., Wilson, I.A., Binley, J.M., Dawson, P.E., Burton, D.R., and Zwick, M.B. (2007). An affinity-enhanced neutralizing antibody against the membrane-proximal external region of human immunodeficiency virus type 1 gp41 recognizes an epitope between those of 2F5 and 4E10. J Virol *81*, 4033-4043. 10.1128/JVI.02588-06.

25. Kim, S., Chen, J., Cheng, T., Gindulyte, A., He, J., He, S., Li, Q., Shoemaker, B.A., Thiessen, P.A., and Yu, B. (2021). PubChem in 2021: new data content and improved web interfaces. Nucleic acids research *49*, D1388-D1395.

26. Yuen, G.J., Lou, Y., Thompson, N.F., Otto, V.R., Allsup, T.L., Mahony, W.B., and Hutman, H.W. (2001). Abacavir/Lamivudine/Zidovudin as a combined formulation tablet: bioequivalence compared with each component administered concurrently and the effect of food on absorption. The Journal of Clinical Pharmacology *41*, 277-288.

27. DiCenzo, R., Forrest, A., Squires, K.E., Hammer, S.M., Fischl, M.A., Wu, H., Cha, R., Morse, G.D., and Adult, A.C.T.G.P.S.T. (2003). Indinavir, efavirenz, and abacavir pharmacokinetics in human immunodeficiency virus-infected subjects. Antimicrob Agents Chemother *47*, 1929-1935. 10.1128/AAC.47.6.1929-1935.2003.

28. Pretorius, E., Klinker, H., and Rosenkranz, B. (2011). The role of therapeutic drug monitoring in the management of patients with human immunodeficiency virus infection. Therapeutic drug monitoring *33*, 265-274.

29. Sax, P.E., Pozniak, A., Montes, M.L., Koenig, E., DeJesus, E., Stellbrink, H.-J., Antinori, A., Workowski, K., Slim, J., and Reynes, J. (2017). Coformulated bictegravir, emtricitabine, and tenofovir alafenamide versus dolutegravir with emtricitabine and tenofovir alafenamide, for initial treatment of HIV-1 infection (GS-US-380–1490): a randomised, double-blind, multicentre, phase 3, non-inferiority trial. Lancet *390*, 2073-2082.

30. Moore, K.H., Yuen, G.J., Raasch, R.H., Eron, J.J., Martin, D., Mydlow, P.K., and Hussey, E.K. (1996). Pharmacokinetics of lamivudine administered alone and with trimethoprim-sulfamethoxazole. Clin Pharmacol Ther *59*, 550-558. 10.1016/S0009-9236(96)90183-6.

31. Calcagno, A., Gonzalez de Requena, D., Simiele, M., D'Avolio, A., Tettoni, M.C., Salassa, B., Orofino, G., Bramato, C., Libanore, V., Motta, I., Bigliano, P., Orsucci, E., Di Perri, G., and Bonora, S. (2013). Tenofovir plasma concentrations according to companion drugs: a cross-sectional study of HIV-positive patients with normal renal function. Antimicrob Agents Chemother *57*, 1840-1843. 10.1128/AAC.02434-12.

32. Boffito, M., Back, D.J., Blaschke, T.F., Rowland, M., Bertz, R.J., Gerber, J.G., and Miller, V. (2003). Protein binding in antiretroviral therapies. AIDS Res Hum Retroviruses *19*, 825-835. 10.1089/088922203769232629.
33. Ramachandran, G., Hemanthkumar, A.K., Kumaraswami, V., and Swaminathan, S. (2006). A simple and rapid liquid chromatography method for simultaneous determination of zidovudine and nevirapine in plasma. J Chromatogr B Analyt Technol Biomed Life Sci *843*, 339-344. 10.1016/j.jchromb.2006.06.014.
34. Yee, K.L., Sanchez, R.I., Auger, P., Liu, R., Fan, L., Triantafyllou, I., Lai, M.T., Di Spirito, M., Iwamoto, M., and Khalilieh, S.G. (2017). Evaluation of Doravirine Pharmacokinetics When Switching from Efavirenz to Doravirine in Healthy Subjects. Antimicrob Agents Chemother *61*. 10.1128/AAC.01757-16.
35. Khalilieh, S., Yee, K.L., Liu, R., Fan, L., Sanchez, R.I., Auger, P., Triantafyllou, I., Stypinski, D., Lasseter, K.C., Marbury, T., and Iwamoto, M. (2017). Moderate Hepatic Impairment Does Not Affect Doravirine Pharmacokinetics. J Clin Pharmacol *57*, 777-783. 10.1002/jcph.857.
36. Foca, M., Yogev, R., Wiznia, A., Hazra, R., Jean-Philippe, P., Graham, B., Britto, P., Carey, V.J., King, J., Acosta, E.P., Cressey, T.R., and Team, I.P. (2016). Rilpivirine Pharmacokinetics Without and With Darunavir/Ritonavir Once Daily in Adolescents and Young Adults. Pediatr Infect Dis J *35*, e271-274. 10.1097/INF.0000000000001214.
37. Aouri, M., Barcelo, C., Guidi, M., Rotger, M., Cavassini, M., Hizrel, C., Buclin, T., Decosterd, L.A., Csajka, C., and Swiss, H.I.V.C.S. (2017). Population Pharmacokinetics and Pharmacogenetics Analysis of Rilpivirine in HIV-1-Infected Individuals. Antimicrob Agents Chemother *61*. 10.1128/AAC.00899-16.
38. Janssen, P.A., Lewi, P.J., Arnold, E., Daeyaert, F., De Jonge, M., Heeres, J., Koymans, L., Vinkers, M., Guillemont, J., and Pasquier, E. (2005). In search of a novel anti-HIV drug: multidisciplinary coordination in the discovery of 4-[[4-[[4-[(1 E)-2-cyanoethenyl]-2, 6-dimethylphenyl] amino]-2-pyrimidinyl] amino] benzonitrile (R278474, rilpivirine). J Med Chem *48*, 1901-1909.
39. Gallant, J.E., Thompson, M., DeJesus, E., Voskuhl, G.W., Wei, X., Zhang, H., White, K., Cheng, A., Quirk, E., and Martin, H. (2017). Antiviral Activity, Safety, and Pharmacokinetics of Bictegravir as 10-Day Monotherapy in HIV-1-Infected Adults. J Acquir Immune Defic Syndr *75*, 61-66. 10.1097/QAI.0000000000001306.
40. Elliot, E., Amara, A., Jackson, A., Moyle, G., Else, L., Khoo, S., Back, D., Owen, A., and Boffito, M. (2016). Dolutegravir and elvitegravir plasma concentrations following cessation of drug intake. J Antimicrob Chemother *71*, 1031-1036. 10.1093/jac/dkv425.
41. Cattaneo, D., Minisci, D., Cozzi, V., Riva, A., Meraviglia, P., Clementi, E., Galli, M., and Gervasoni, C. (2017). Dolutegravir plasma concentrations according to companion antiretroviral drug: unwanted drug interaction or desirable boosting effect? Antivir Ther *22*, 353-356. 10.3851/IMP3119.
42. Cottrell, M.L., Hadzic, T., and Kashuba, A.D. (2013). Clinical pharmacokinetic, pharmacodynamic and drug-interaction profile of the integrase inhibitor dolutegravir. Clin Pharmacokinet *52*, 981-994. 10.1007/s40262-013-0093-2.
43. Huhn, G.D., Tebas, P., Gallant, J., Wilkin, T., Cheng, A., Yan, M., Zhong, L., Callebaut, C., Custodio, J.M., Fordyce, M.W., Das, M., and McCallister, S. (2017). A Randomized, Open-Label Trial to Evaluate Switching to Elvitegravir/Cobicistat/Emtricitabine/Tenofovir Alafenamide Plus Darunavir in Treatment-Experienced HIV-1-Infected Adults. J Acquir Immune Defic Syndr *74*, 193-200. 10.1097/QAI.0000000000001193.
44. Calcagno, A., Simiele, M., Motta, I., Mornese Pinna, S., Bertucci, R., D'Avolio, A., Di Perri, G., and Bonora, S. (2016). Elvitegravir/Cobicistat/Tenofovir/Emtricitabine Penetration in the Cerebrospinal Fluid of Three HIV-Positive Patients. AIDS Res Hum Retroviruses *32*, 409-411. 10.1089/aid.2015.0337.
